# Supplementary material for: Dipterocarpoidae genomics reveal their demography and adaptations to Asian rainforests
Source: Nat Commun. 2024 Feb 23;15:1683. doi: 10.1038/s41467-024-45836-5 (PMC10891123; doi:10.1038/s41467-024-45836-5)
Supplement: Supplementary file 1 — Supplementary information [file 41467_2024_45836_MOESM1_ESM.pdf]

Supplemental information

Title:

Dipterocarpoidae genomics reveal their demography and adaptations to Asian rainforests

Author list:

Rong Wang, Chao-Nan Liu, Simon T Segar, Yu-Ting Jiang, Kai-Jian Zhang, Kai Jiang, Gang Wang, Jing Cai, Lu-Fan Chen, Shan Chen, Jing Cheng, Stephen G Compton, Jun-Yin Deng, Yuan-Yuan Ding, Fang K. Du, Xiao-Di Hu, Xing-Hua Hu, Ling Kang, Dong-Hai Li, Ling Lu, Yuan-Yuan Li, Liang Tang, Xin Tong, Zheng-Shi Wang, Wei-Wei Xu, Yang Yang, Run-Guo Zang, Zhuo-Xin Zu, Yuan-Ye Zhang & Xiao-Yong Chen

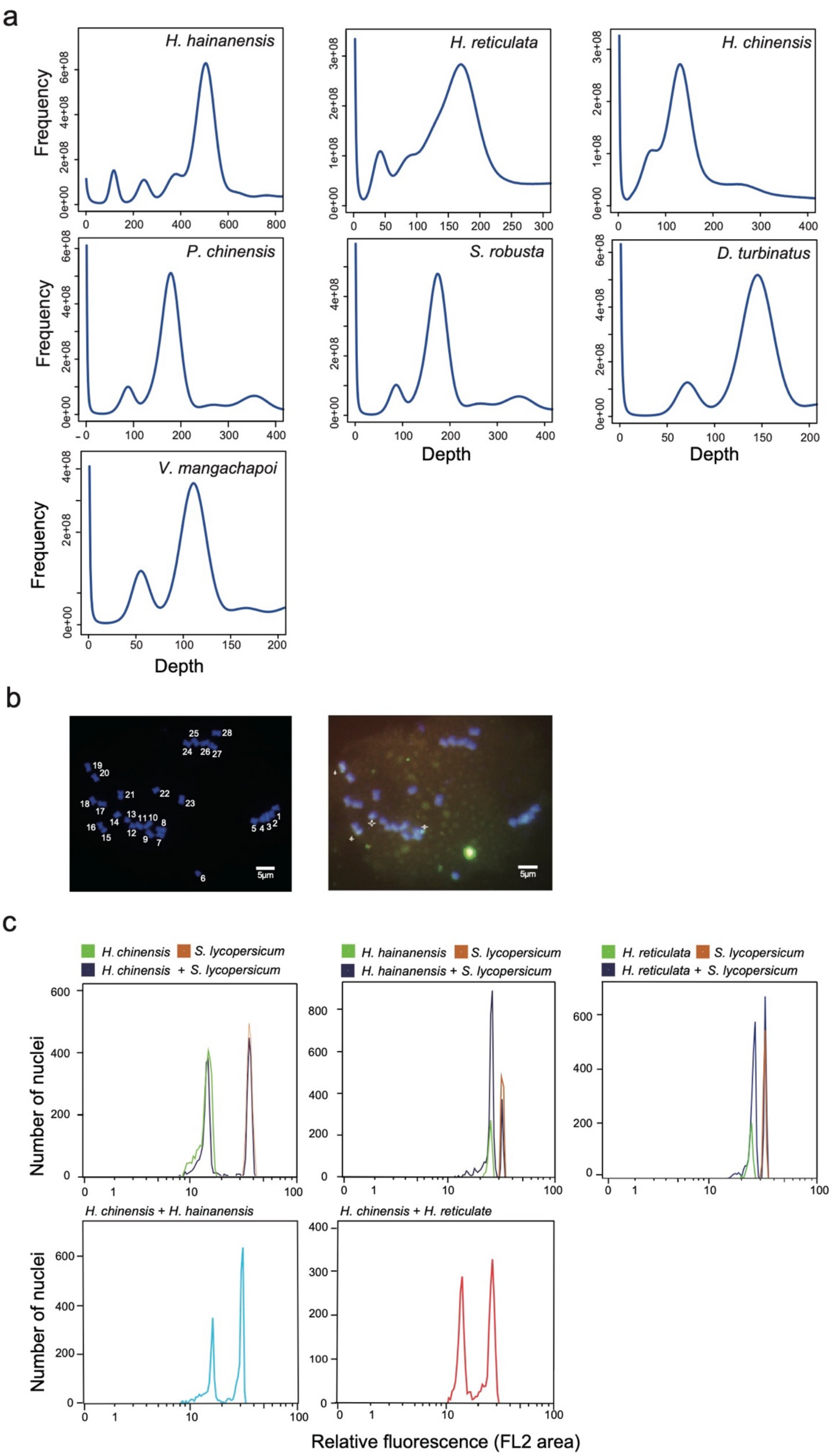

a
H. hainanensis
H. reticulata
H. chinensis
P. chinensis
S. robusta
D. turbinatus
V. mangachapoi
Frequency
Depth
b
5μm
c
H. chinensis
S. lycopersicum
H. chinensis + S. lycopersicum
H. hainanensis
H. hainanensis + S. lycopersicum
H. reticulata
H. reticulata + S. lycopersicum
H. chinensis + H. hainanensis
H. chinensis + H. reticulate
Number of nuclei
Relative fluorescence (FL2 area)

d

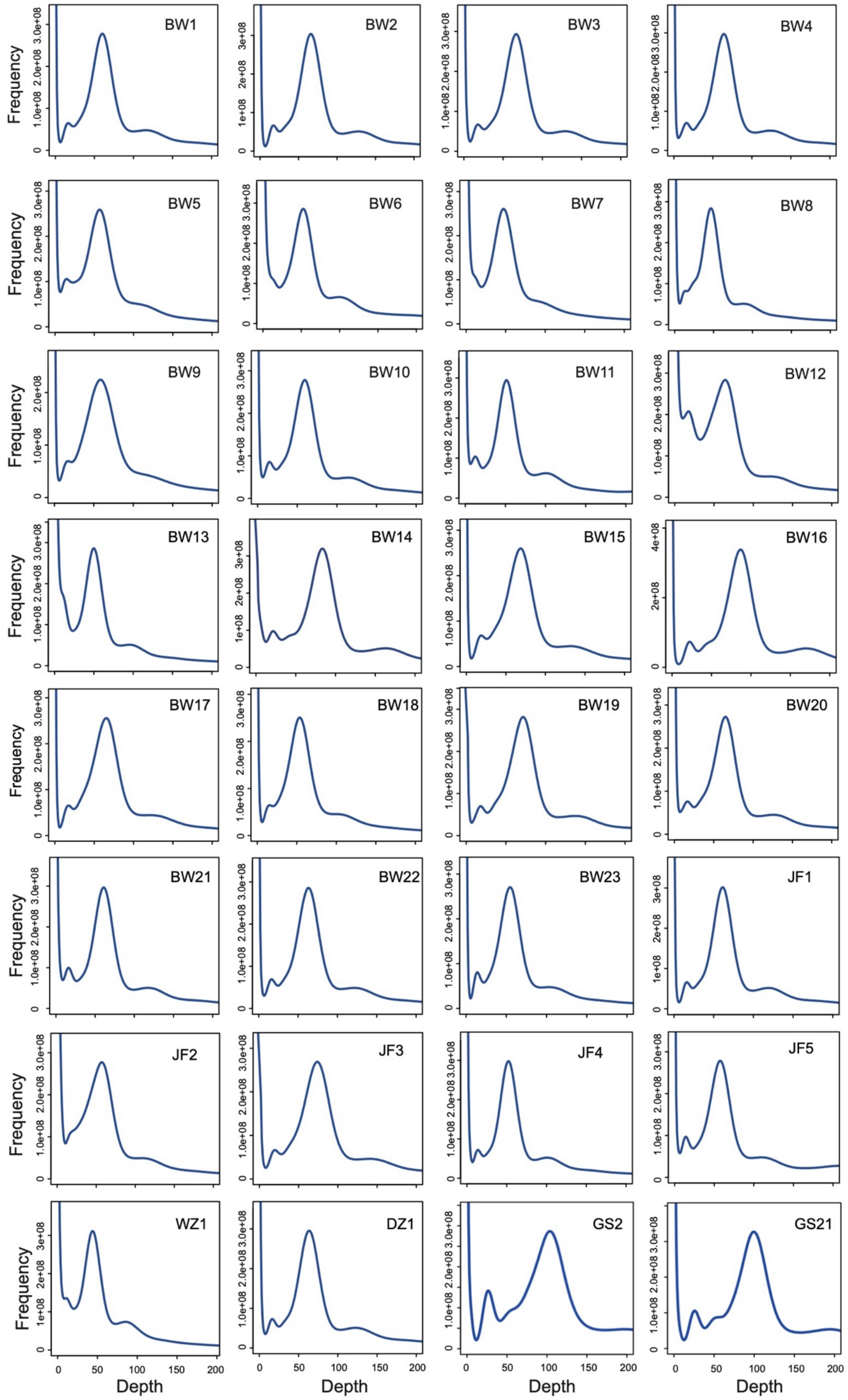
BW1
BW2
BW3
BW4
BW5
BW6
BW7
BW8
BW9
BW10
BW11
BW12
BW13
BW14
BW15
BW16
BW17
BW18
BW19
BW20
BW21
BW22
BW23
JF1
JF2
JF3
JF4
JF5
WZ1
DZ1
GS2
GS21
Frequency
Depth
0
50
100
150
200
1.0e+08
2.0e+08
3.0e+08

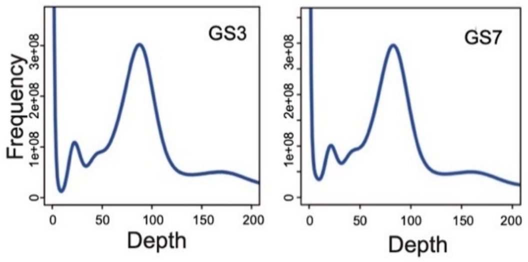

Supplementary Figure 1. ***k*****-mer distributions of the 7 Dipterocarpoideae genomes (a), the results of karyotype analysis of *H. hainanensis* using fluorescence *in situ* hybridization (b), the results of flow cytometry (c), and *k*-mer distributions of all sampled trees of *H. hainanensis* (n=30) and the selected four *H. reticulata* trees (d).** In the *k*-mer analysis, the ploidy of a species is assessed via the ratio of the depth of the leftmost peak to that of the rightmost peak, and the results suggest that *H. hainanensis* and *H. reticulata* are tetraploid species and the other species are diploids. The details of Illumina sequencing data used for the *k*-mer analysis are shown in Supplementary Tables 2 and 16. The chromosome number of *H. hainanensis* was identified as 28, which was four times as the basic chromosome number in genus *Hopea* (n=7)[1]. The green fluorescence signal (highlighted by crosses in Supplementary Fig. 1b) were detected in all the four chromosomes using *in situ* hybridization with the probe of 18SrDNA (this experiment was performed for one time), further confirming that there are four copies of basic chromosomes in *H. hainanensis*, i.e., autotetraploidy. When estimating the genome size for *H. Chinesis*, *H. hainanensis* and *H. reticulata*, we used the genome of *Solanum lycopersicum* (2C = 2.12) as the reference for flow cytometry experiments. When estimating genome size, we ran individual sample for each of the three *Hopea* species (*H. Chinesis*, *H. hainanensis* and *H. reticulata*) and *S. lycopersicum* separately, and ran samples combining each *Hopea* species with *S. lycopersicum* (Supplementary Fig 1c (the upper panel) and Supplementary Table 1). *H. Chinesis* was chosen as the reference diploid species for the estimation of ploidy of *H. hainanensis* and *H. reticulata*. Each treatment of flow cytometry experiments were repeated for three times with similar results.

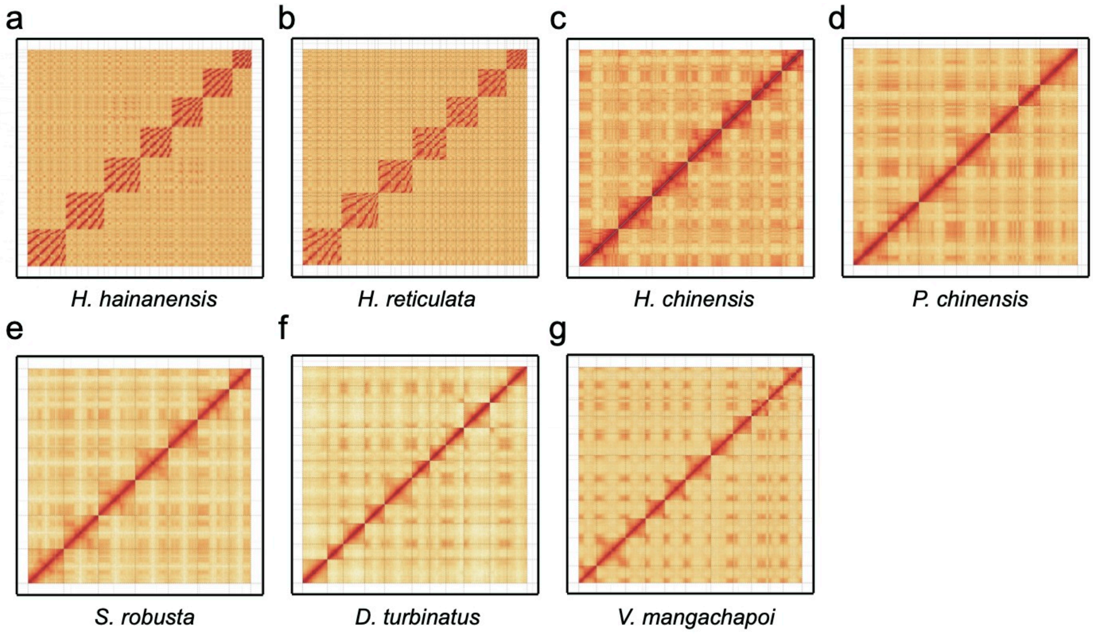

Supplementary Figure 2. Heat maps of interaction intensity between the pseudo-chromosomes clustered by Hi-C reads for the 7 Dipterocarpoideae species.

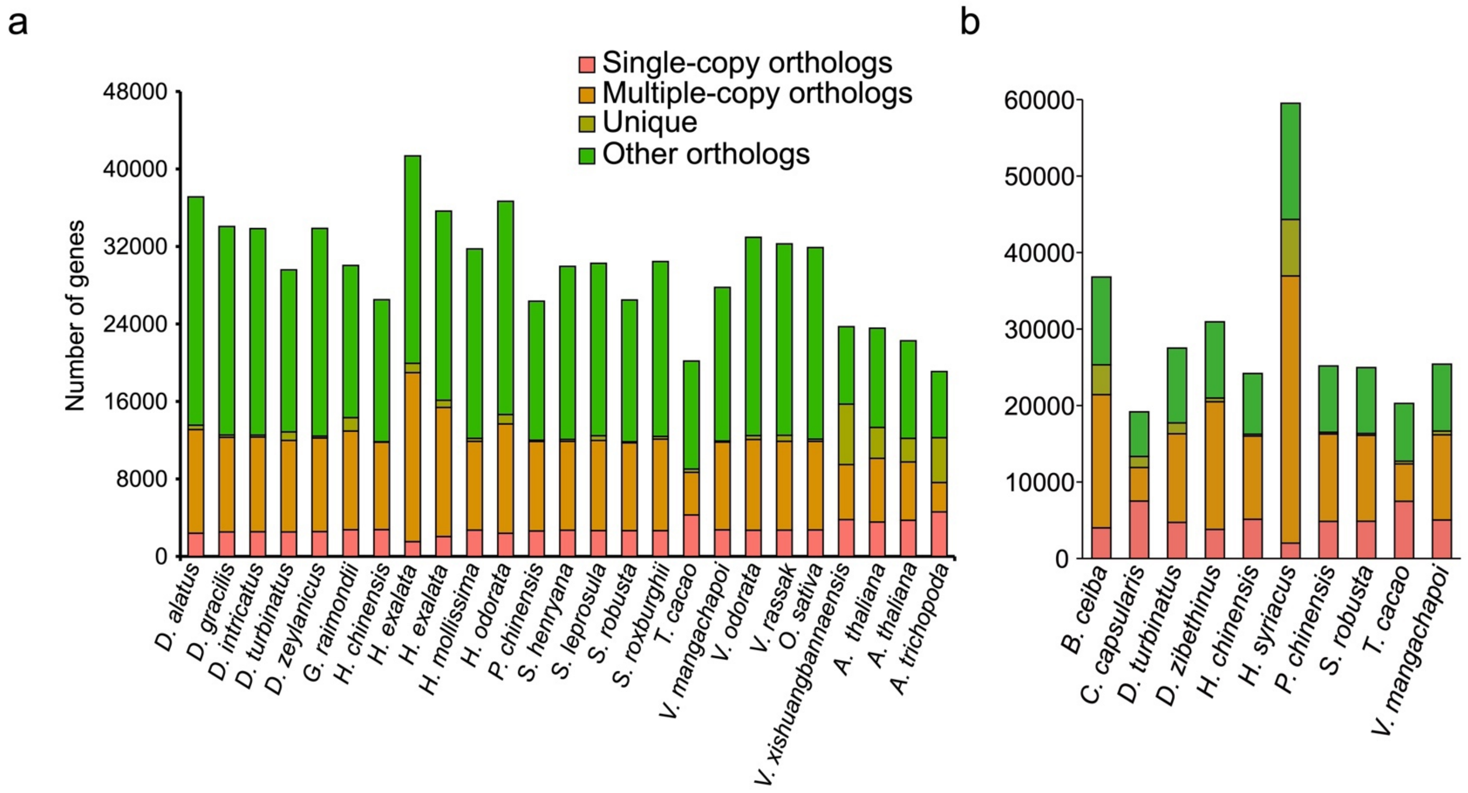

Supplementary Figure 3. Results of gene family clustering based on the genomes of our focal 7 Dipterocarpoideae species, other 12 Dipterocarpoideae species and four species from other plant taxa (a) and those of the 5 diploid Dipterocarpoideae species and 5 temperate tree species (b). Full names of each species and the references reported the assembled genomes are listed in Supplementary Table 9. Source data are provided as a Source Data file.

a

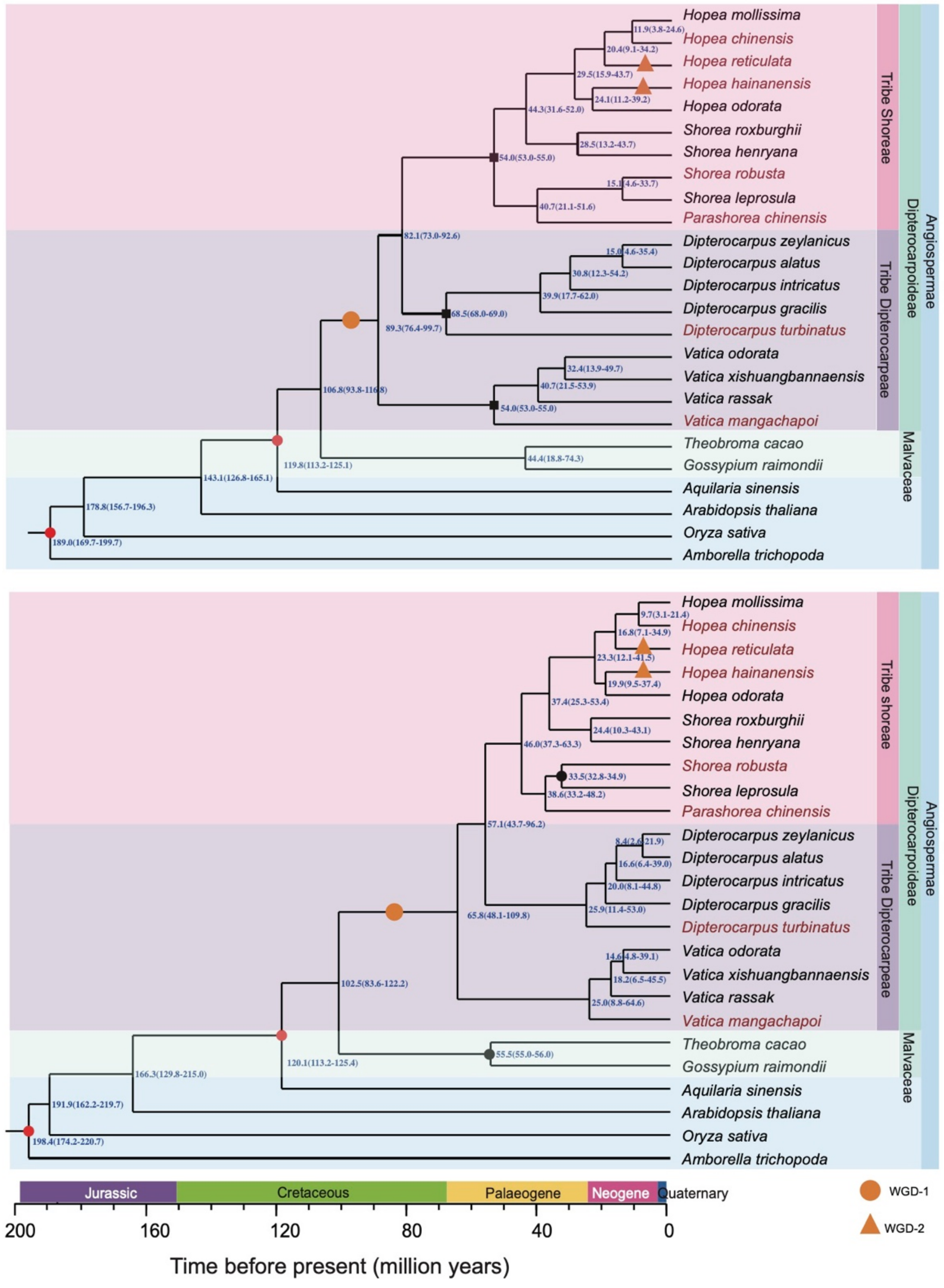

Hopea mollissima
Hopea chinensis
Hopea reticulata
Hopea hainanensis
Hopea odorata
Shorea roxburghii
Shorea henryana
Shorea robusta
Shorea leprosula
Parashorea chinensis
Dipterocarpus zeylanicus
Dipterocarpus alatus
Dipterocarpus intricatus
Dipterocarpus gracilis
Dipterocarpus turbinatus
Vatica odorata
Vatica xishuangbannaensis
Vatica rassak
Vatica mangachapoi
Theobroma cacao
Gossypium raimondii
Aquilaria sinensis
Arabidopsis thaliana
Oryza sativa
Amborella trichopoda
Tribe Shoreae
Tribe Dipterocarpeae
Dipterocarpoideae
Malvaceae
Angiospermae
Hopea mollissima
Hopea chinensis
Hopea reticulata
Hopea hainanensis
Hopea odorata
Shorea roxburghii
Shorea henryana
Shorea robusta
Shorea leprosula
Parashorea chinensis
Dipterocarpus zeylanicus
Dipterocarpus alatus
Dipterocarpus intricatus
Dipterocarpus gracilis
Dipterocarpus turbinatus
Vatica odorata
Vatica xishuangbannaensis
Vatica rassak
Vatica mangachapoi
Theobroma cacao
Gossypium raimondii
Aquilaria sinensis
Arabidopsis thaliana
Oryza sativa
Amborella trichopoda
Tribe shoreae
Tribe Dipterocarpeae
Dipterocarpoideae
Malvaceae
Angiospermae
Jurassic
Cretaceous
Palaeogene
Neogene
Quaternary
200
160
120
80
40
0
Time before present (million years)
WGD-1
WGD-2

b

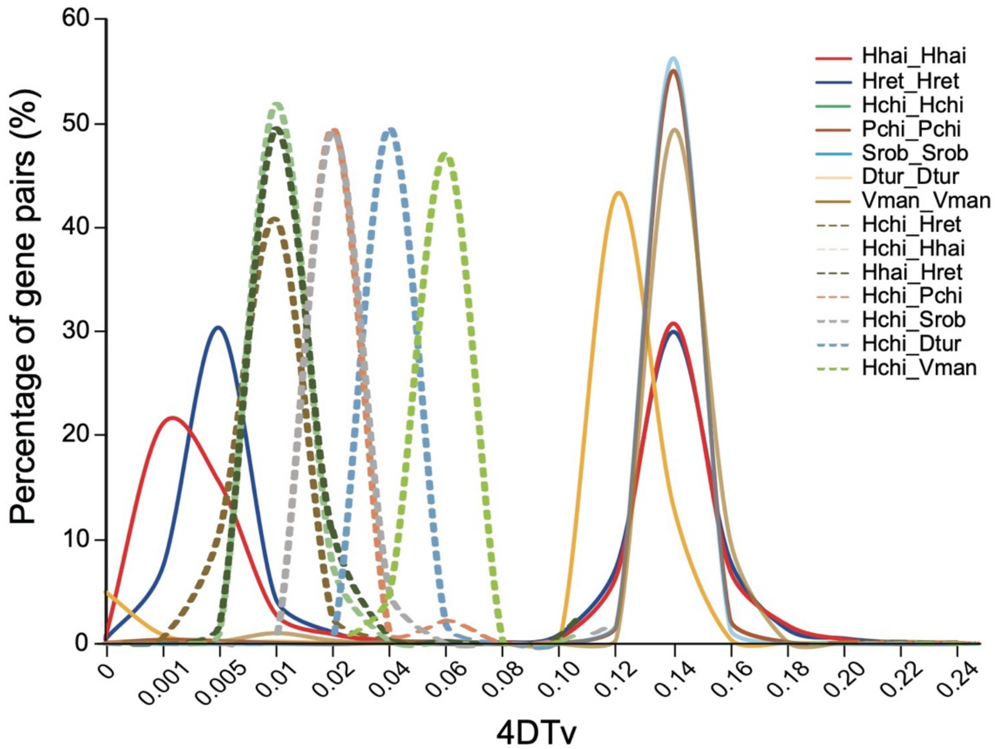

Supplementary Figure 4. Results of phylogenomic analysis using calibrations from Bell et al.[2] and Vega et al.[3] (red dots), Bansal et al.[4] (black squares) and Ng et al.[5] (black dots) (a), and the distribution of transversion rate of four-fold degenerate sites (4DTv) for homologous genes within each genome and between two genomes (b). The genomes assembled in this study are highlighted by red. Abbreviations of species are shown in Supplementary Table 2.

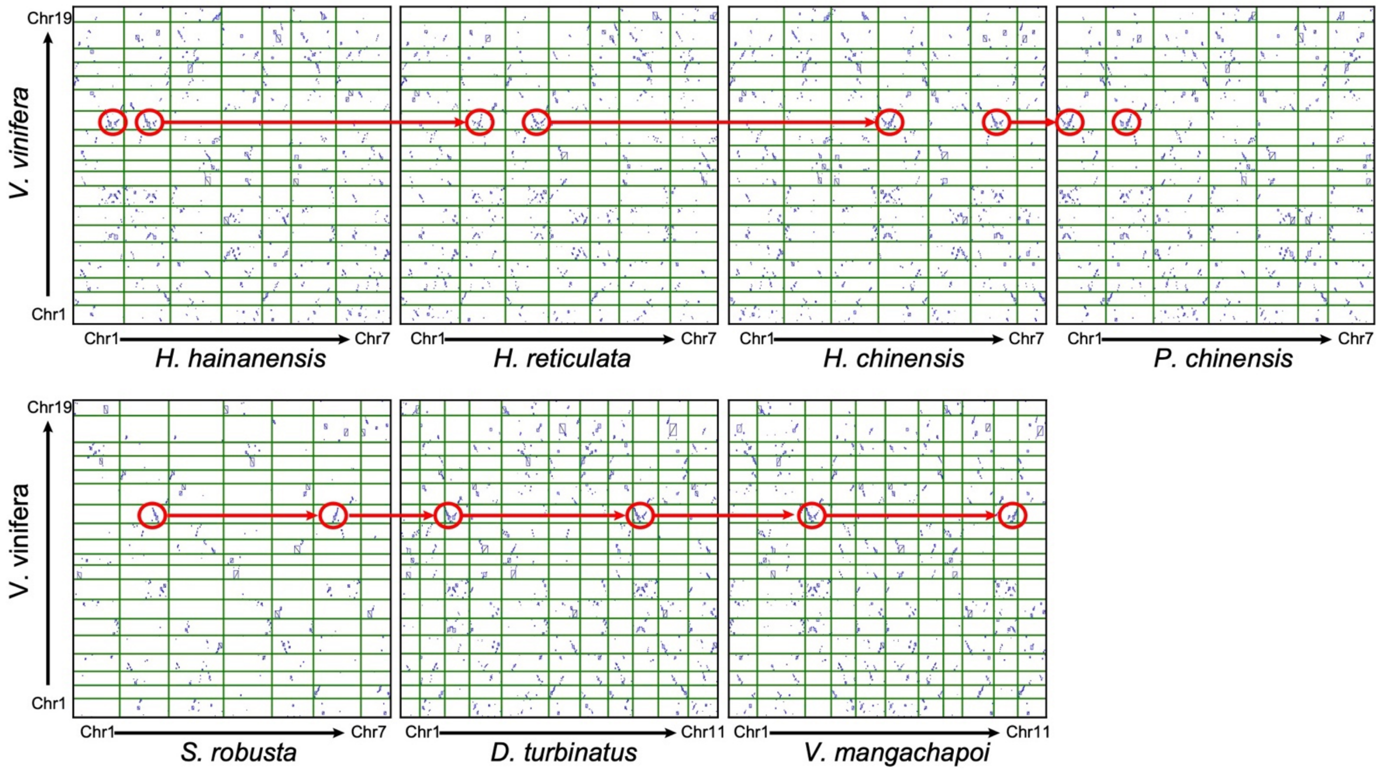

Supplementary Figure 5. Comparison of the homologous regions between the genome of each focal Dipterocarpoideae species and the genome of *Vitis vinifera*. Results show that most homologous regions detected in the genome of *Vitis vinifera* are doubled in the genomes of our focal species.

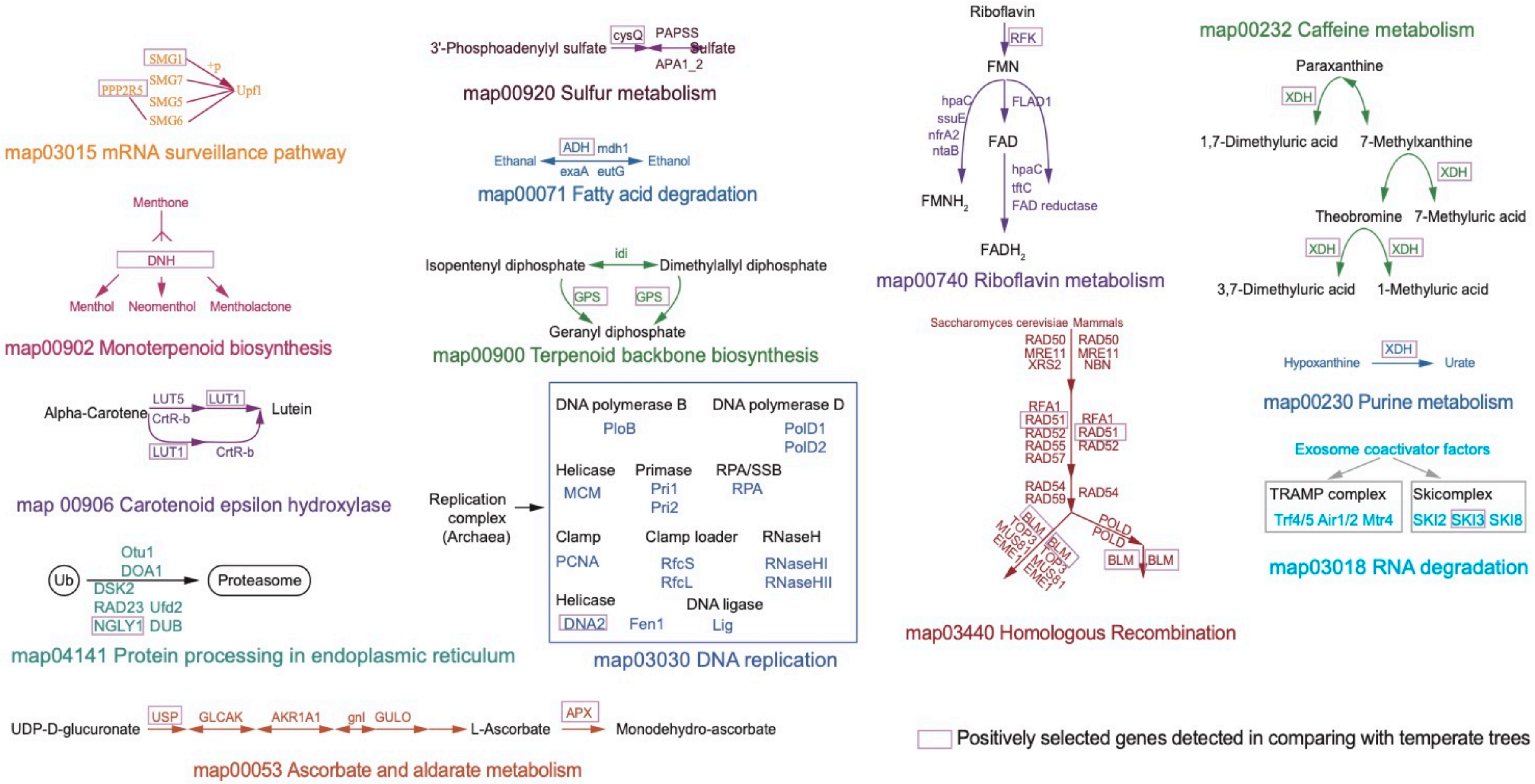

Supplementary Figure 6. Positively selected genes (16) involved in the KEGG pathways relevant to plants' response to environmental stresses. Purple frames are used to highlight the positively selected genes supported by the comparison with the genomes of five temperate tree species. The function of each gene is shown in Supplementary Data 2.

a RAD51D

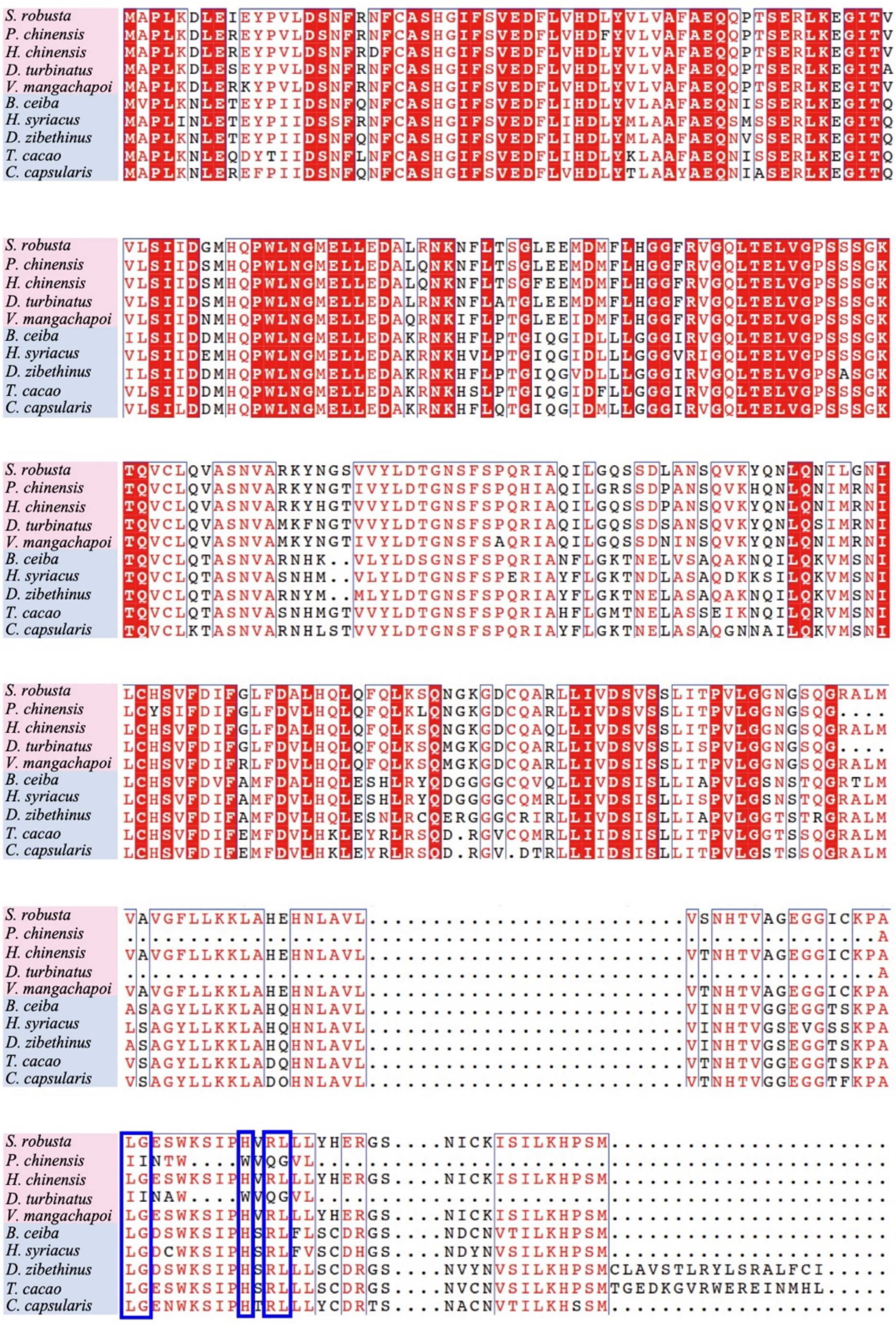
S. robusta
P. chinensis
H. chinensis
D. turbinatus
V. mangachapoi
B. ceiba
H. syriacus
D. zibethinus
T. cacao
C. capsularis

RAD51D

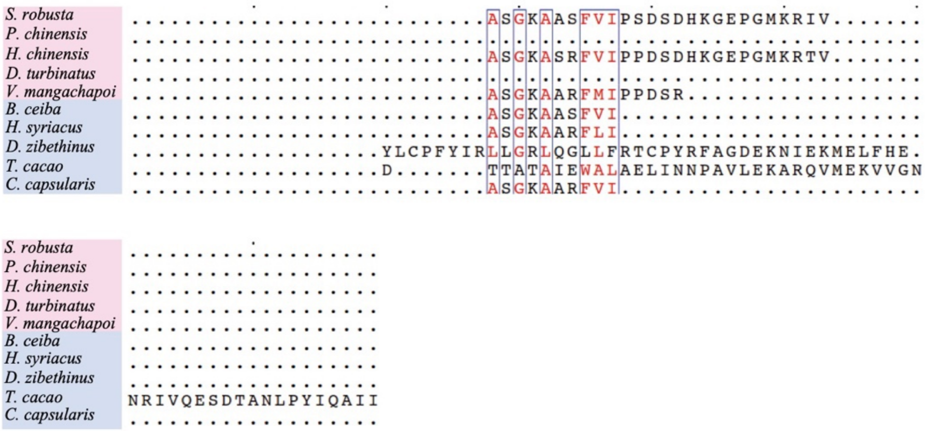

S. robusta
P. chinensis
H. chinensis
D. turbinatus
V. mangachapoi
B. ceiba
H. syriacus
D. zibethinus
T. cacao
C. capsularis
ASGKAASFVIPSDSDHKGEPGMKRIV
ASGKASRFVIPPDSDHKGEPGMKRTV
ASGKAARFMIPPDSR
ASGKAASFVI
ASGKAARFLI
YLCPFYIRLLGRLQGLLFRTCPYRFAGDEKNIEKMELFHE
D
TTATAIEWALAELINNPAVLEKARQVMEKVVGN
ASGKAARFVI
NRIVQESDTANLPYIQAII

b SMC5

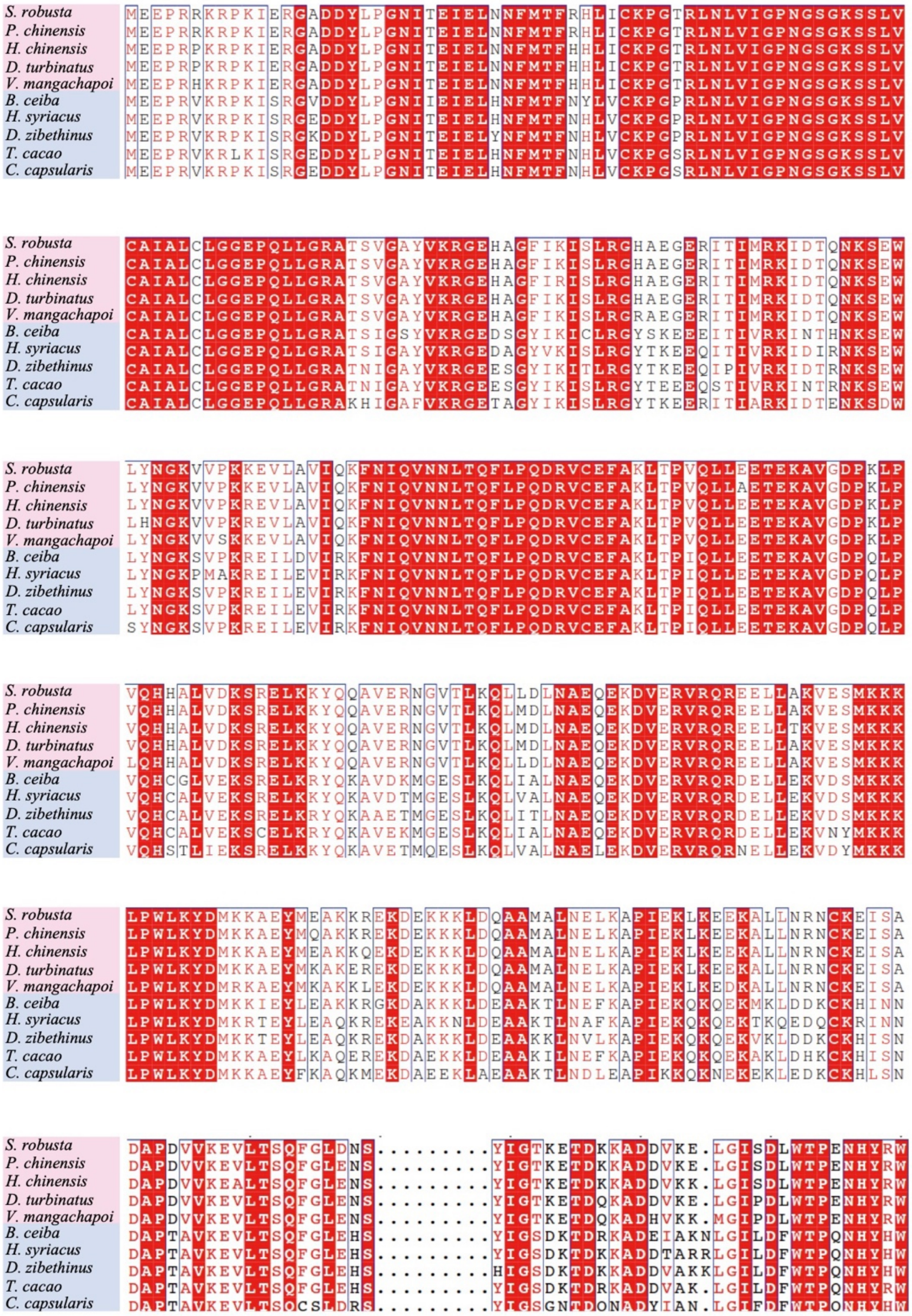
S. robusta
P. chinensis
H. chinensis
D. turbinatus
V. mangachapoi
B. ceiba
H. syriacus
D. zibethinus
T. cacao
C. capsularis

SMC5

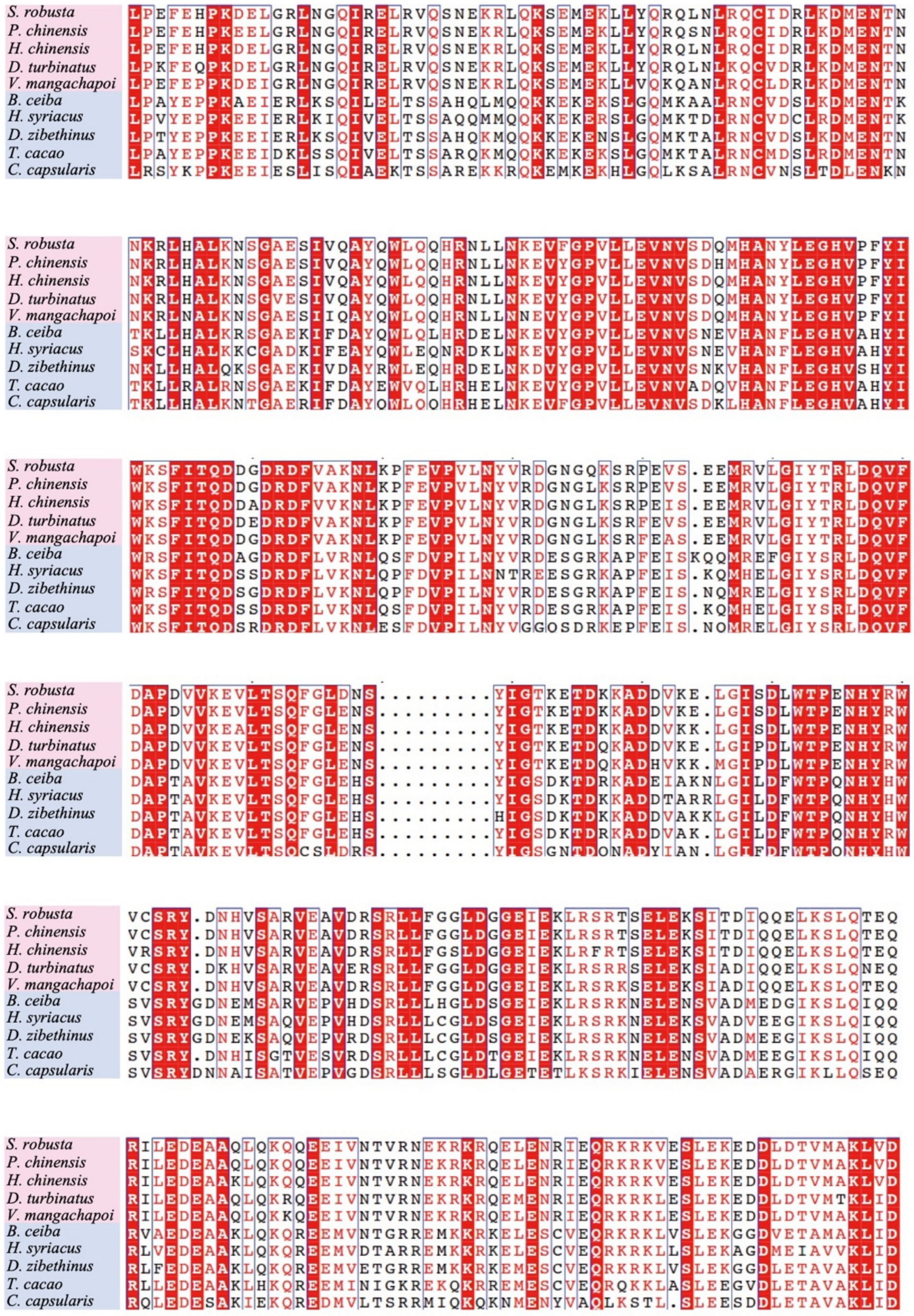
S. robusta
P. chinensis
H. chinensis
D. turbinatus
V. mangachapoi
B. ceiba
H. syriacus
D. zibethinus
T. cacao
C. capsularis

SMC5

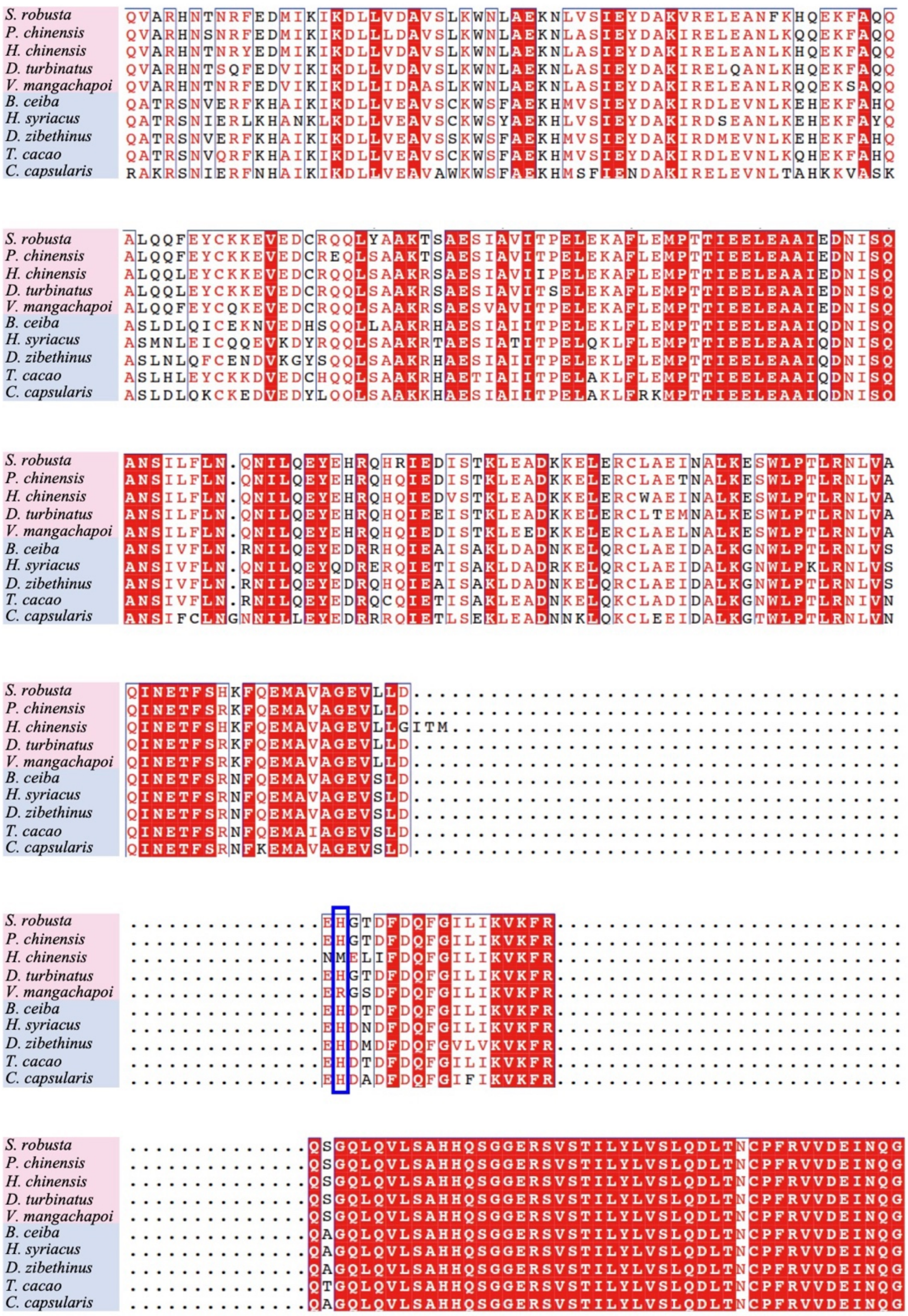
S. robusta
P. chinensis
H. chinensis
D. turbinatus
V. mangachapoi
B. ceiba
H. syriacus
D. zibethinus
T. cacao
C. capsularis

SMC5

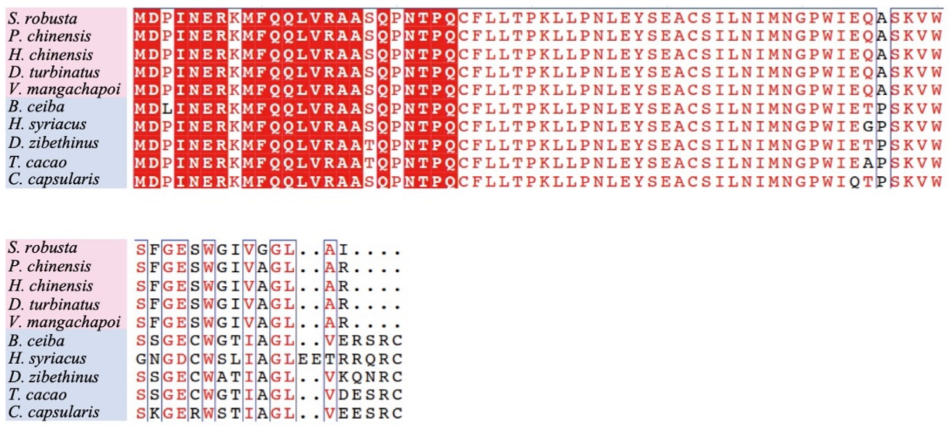

S. robusta MDPINERKMFQQLVRAASQPNTPQCFLLTPKLLPNLEYSEACSILNIMNGPWIEQASKVW
P. chinensis MDPINERKMFQQLVRAASQPNTPQCFLLTPKLLPNLEYSEACSILNIMNGPWIEQASKVW
H. chinensis MDPINERKMFQQLVRAASQPNTPQCFLLTPKLLPNLEYSEACSILNIMNGPWIEQASKVW
D. turbinatus MDPINERKMFQQLVRAASQPNTPQCFLLTPKLLPNLEYSEACSILNIMNGPWIEQASKVW
V. mangachapoi MDPINERKMFQQLVRAASQPNTPQCFLLTPKLLPNLEYSEACSILNIMNGPWIEQASKVW
B. ceiba MDLINERKMFQQLVRAASQPNTPQCFLLTPKLLPNLEYSEACSILNIMNGPWIETPSKVW
H. syriacus MDPINERKMFQQLVRAASQPNTPQCFLLTPKLLPNLEYSEACSILNIMNGPWIEGPSKVW
D. zibethinus MDPINERKMFQQLVRAATQPNTPQCFLLTPKLLPNLEYSEACSILNIMNGPWIETPSKVW
T. cacao MDPINERKMFQQLVRAATQPNTPQCFLLTPKLLPNLEYSEACSILNIMNGPWIEAPSKVW
C. capsularis MDPINERKMFQQLVRAASQPNTPQCFLLTPKLLPNLEYSEACSILNIMNGPWIQTPSKVW
S. robusta SFGESWGIVGGL..AI....
P. chinensis SFGESWGIVAGL..AR....
H. chinensis SFGESWGIVAGL..AR....
D. turbinatus SFGESWGIVAGL..AR....
V. mangachapoi SFGESWGIVAGL..AR....
B. ceiba SSGECWGTIAGL..VERSRC
H. syriacus GNGDCWSLIAGLEETRRQRC
D. zibethinus SSGECWATIAGL..VKQNRC
T. cacao SSGECWGTIAGL..VDESRC
C. capsularis SKGERWSTIAGL..VEESRC

C LUT1

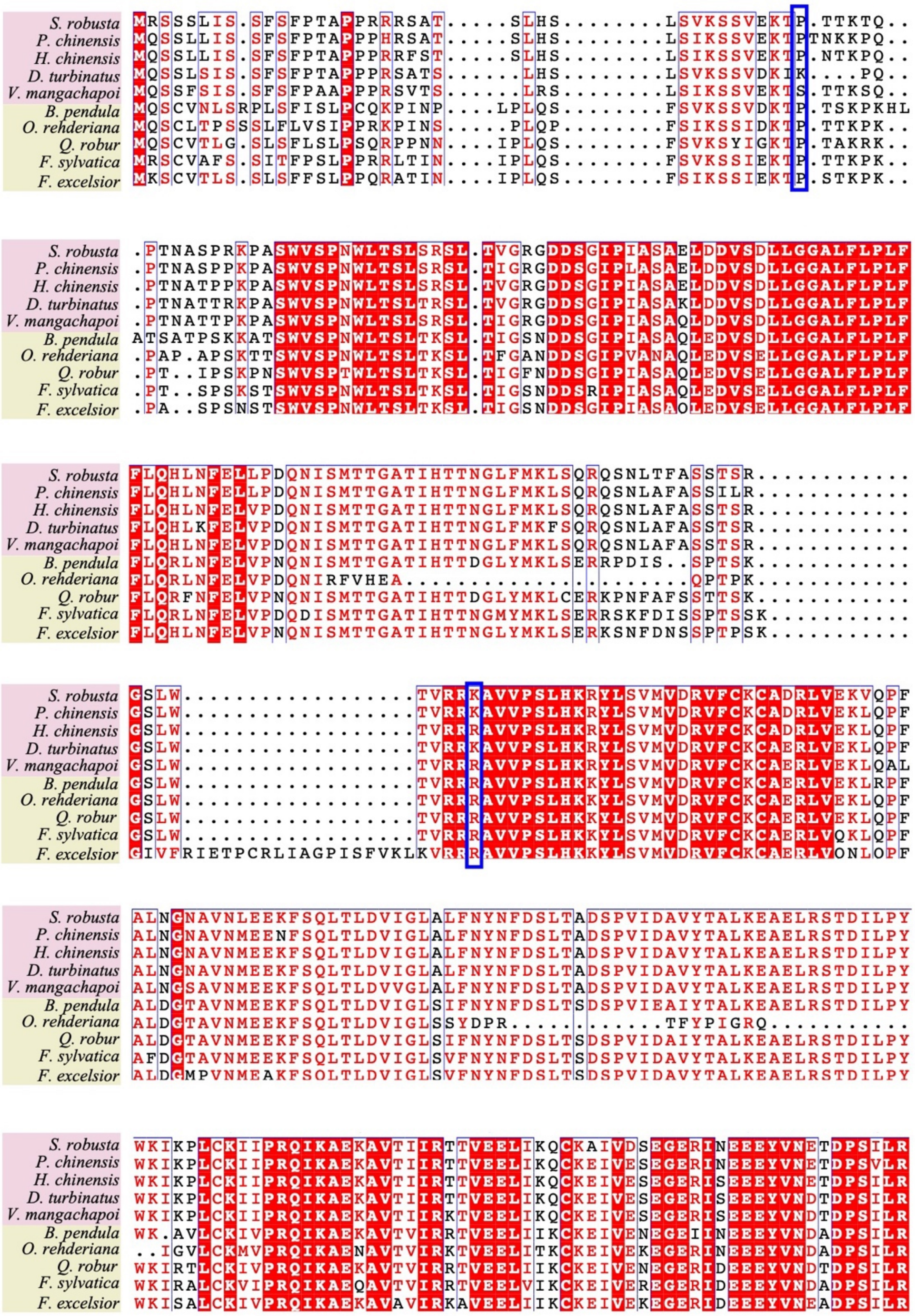
S. robusta
P. chinensis
H. chinensis
D. turbinatus
V. mangachapoi
B. pendula
O. rehderiana
Q. robur
F. sylvatica
F. excelsior

LUT1

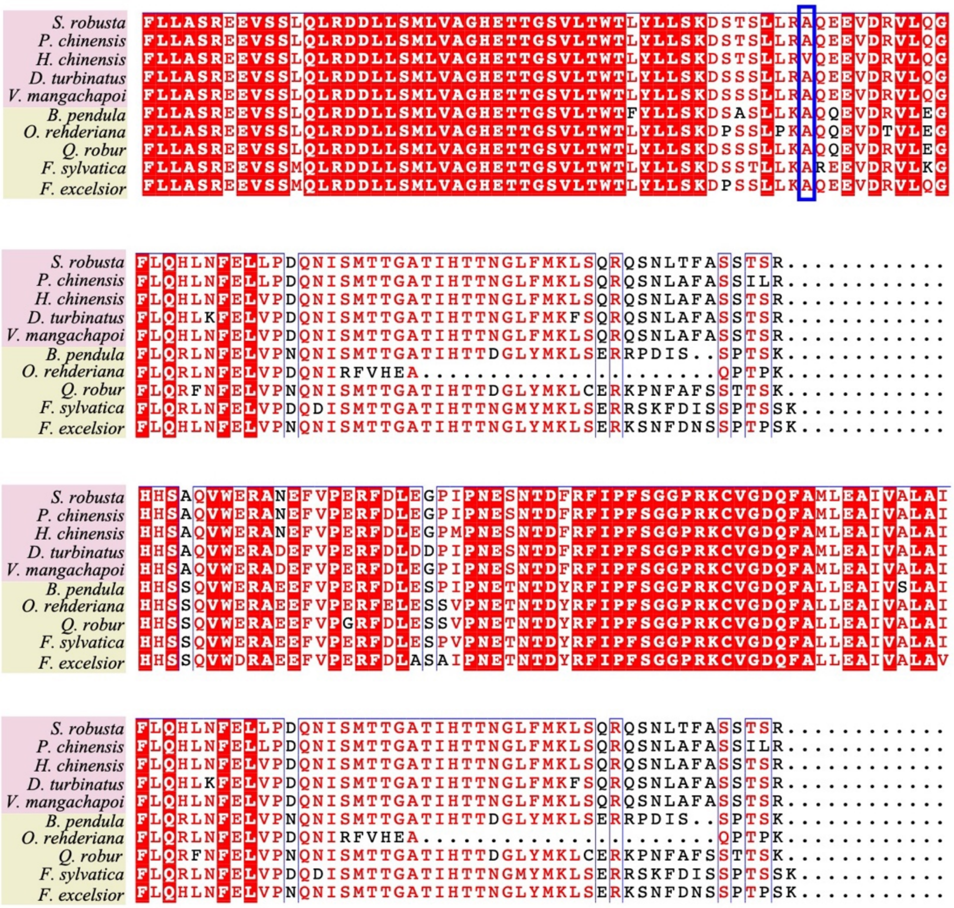
S. robusta FLLASREEVSSLQLRDDLLSMLVAGHETTGSVLTWTLYLLSKDSTSLLRAQEEVDRVLQG
P. chinensis FLLASREEVSSLQLRDDLLSMLVAGHETTGSVLTWTLYLLSKDSTSLLRAQEEVDRVLQG
H. chinensis FLLASREEVSSLQLRDDLLSMLVAGHETTGSVLTWTLYLLSKDSTSLLRVQEEVDRVLQG
D. turbinatus FLLASREEVSSLQLRDDLLSMLVAGHETTGSVLTWTLYLLSKDSSSLLRAQEEVDRVLQG
V. mangachapoi FLLASREEVSSLQLRDDLLSMLVAGHETTGSVLTWTLYLLSKDSSSLLRAQEEVDRVLQG
B. pendula FLLASREEVSSLQLRDDLLSMLVAGHETTGSVLTWTFYLLSKDSASLLKAQQEVDRVLEG
O. rehderiana FLLASREEVSSLQLRDDLLSMLVAGHETTGSVLTWTLYLLSKDPSSLPKAQQEVDTVLEG
Q. robur FLLASREEVSSLQLRDDLLSMLVAGHETTGSVLTWTLYLLSKDSSSLLKAQQEVDRVLEG
F. sylvatica FLLASREEVSSMQLRDDLLSMLVAGHETTGSVLTWTLYLLSKDSSTLLKAREEVDRVLKG
F. excelsior FLLASREEVSSMQLRDDLLSMLVAGHETTGSVLTWTLYLLSKDPSSLLKAQEEVDRVLQG
S. robusta FLQHLNFELLPDQNISMTTGATIHTTNGLFMKLSQRQSNLTFASSTSR............
P. chinensis FLQHLNFELLPDQNISMTTGATIHTTNGLFMKLSQRQSNLAFASSILR............
H. chinensis FLQHLNFELVPDQNISMTTGATIHTTNGLFMKLSQRQSNLAFASSTSR............
D. turbinatus FLQHLKFELVPDQNISMTTGATIHTTNGLFMKFSQRQSNLAFASSTSR............
V. mangachapoi FLQHLNFELVPDQNISMTTGATIHTTNGLFMKLSQRQSNLAFASSTSR............
B. pendula FLQRLNFELVPNQNISMTTGATIHTTDGLYMKLSERRPDIS..SPTSK............
O. rehderiana FLQRLNFELVPDQNIRFVHEA.......................QPTPK............
Q. robur FLQRFNFELVPNQNISMTTGATIHTTDGLYMKLCERKPNFAFSSTTSK............
F. sylvatica FLQRLNFELVPDQDISMTTGATIHTTNGMYMKLSERRSKFDISSPTSSK...........
F. excelsior FLQHLNFELVPNQNISMTTGATIHTTNGLYMKLSERKSNFDNSSPTPSK...........
S. robusta HHSAQVWERANEFVPERFDLEGPIPNESNTDFRFIPFSGGPRKCVGDQFAMLEAIVALAI
P. chinensis HHSAQVWERANEFVPERFDLEGPIPNESNTDFRFIPFSGGPRKCVGDQFAMLEAIVALAI
H. chinensis HHSAQVWERANEFVPERFDLEGPMPNESNTDFRFIPFSGGPRKCVGDQFAMLEAIVALAI
D. turbinatus HHSAQVWERADEFVPERFDLDDPIPNESNTDFRFIPFSGGPRKCVGDQFAMLEAIVALAI
V. mangachapoi HHSAQVWERADEFVPERFDLEGPIPNESNTDFRFIPFSGGPRKCVGDQFAMLEAIVALAI
B. pendula HHSSQVWERAEEFVPERFDLESPIPNETNTDYRFIPFSGGPRKCVGDQFALLEAIVSLAI
O. rehderiana HHSSQVWERAEEFVPERFELESSVPNETNTDYRFIPFSGGPRKCVGDQFALLEAIVALAI
Q. robur HHSSQVWERAEEFVPGRFDLESSVPNETNTDYRFIPFSGGPRKCVGDQFALLEAIVALAI
F. sylvatica HHSSQVWERAEEFVPERFDLESPVPNETNTDYRFIPFSGGPRKCVGDQFALLEAIVALAI
F. excelsior HHSSQVWDRAEEFVPERFDLASAIPNETNTDYRFIPFSGGPRKCVGDQFALLEAIVALAV
S. robusta FLQHLNFELLPDQNISMTTGATIHTTNGLFMKLSQRQSNLTFASSTSR............
P. chinensis FLQHLNFELLPDQNISMTTGATIHTTNGLFMKLSQRQSNLAFASSILR............
H. chinensis FLQHLNFELVPDQNISMTTGATIHTTNGLFMKLSQRQSNLAFASSTSR............
D. turbinatus FLQHLKFELVPDQNISMTTGATIHTTNGLFMKFSQRQSNLAFASSTSR............
V. mangachapoi FLQHLNFELVPDQNISMTTGATIHTTNGLFMKLSQRQSNLAFASSTSR............
B. pendula FLQRLNFELVPNQNISMTTGATIHTTDGLYMKLSERRPDIS..SPTSK............
O. rehderiana FLQRLNFELVPDQNIRFVHEA.......................QPTPK............
Q. robur FLQRFNFELVPNQNISMTTGATIHTTDGLYMKLCERKPNFAFSSTTSK............
F. sylvatica FLQRLNFELVPDQDISMTTGATIHTTNGMYMKLSERRSKFDISSPTSSK...........
F. excelsior FLQHLNFELVPNQNISMTTGATIHTTNGLYMKLSERKSNFDNSSPTPSK...........

d DNA2

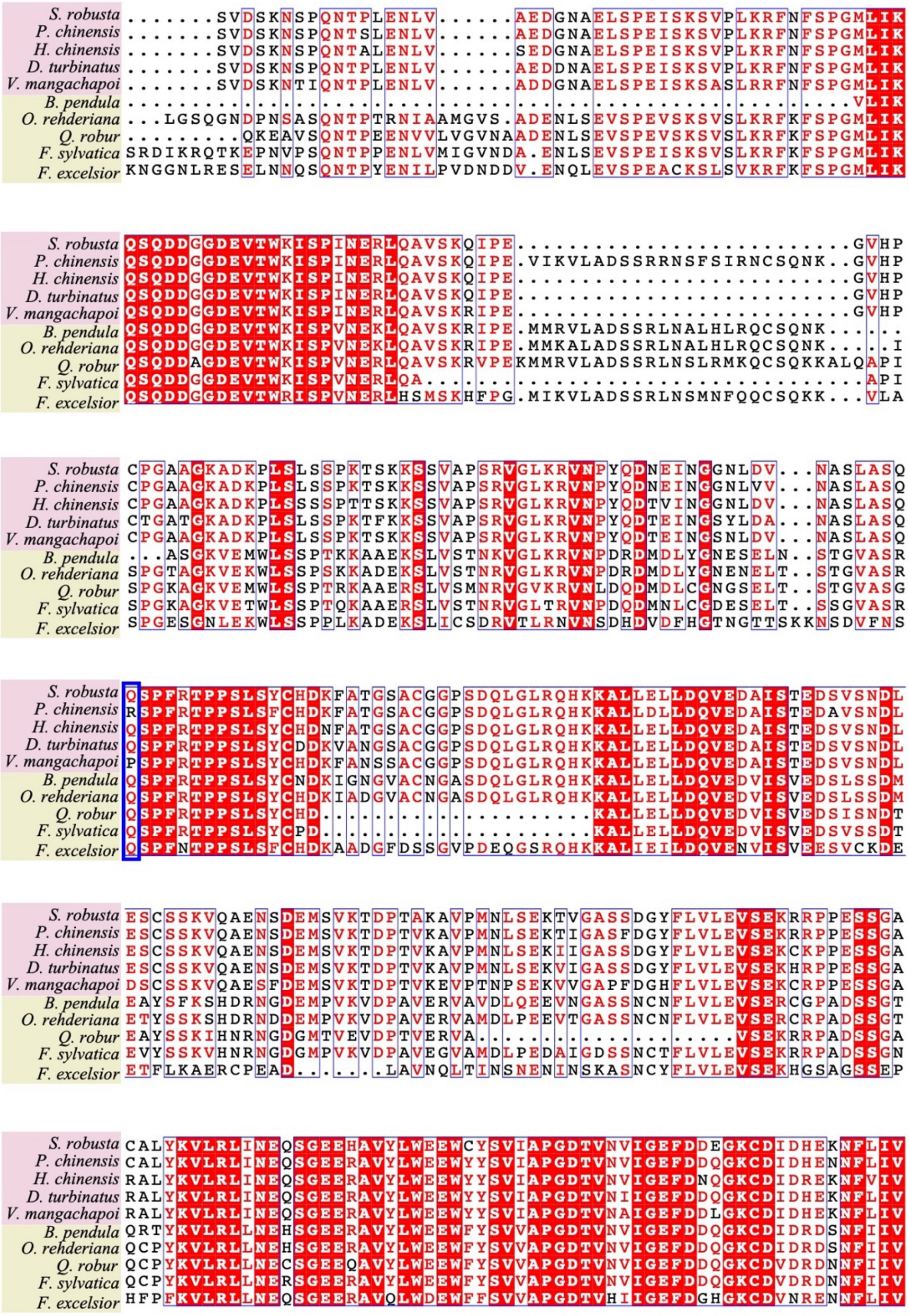

S. robusta
P. chinensis
H. chinensis
D. turbinatus
V. mangachapoi
B. pendula
O. rehderiana
Q. robur
F. sylvatica
F. excelsior

DNA2

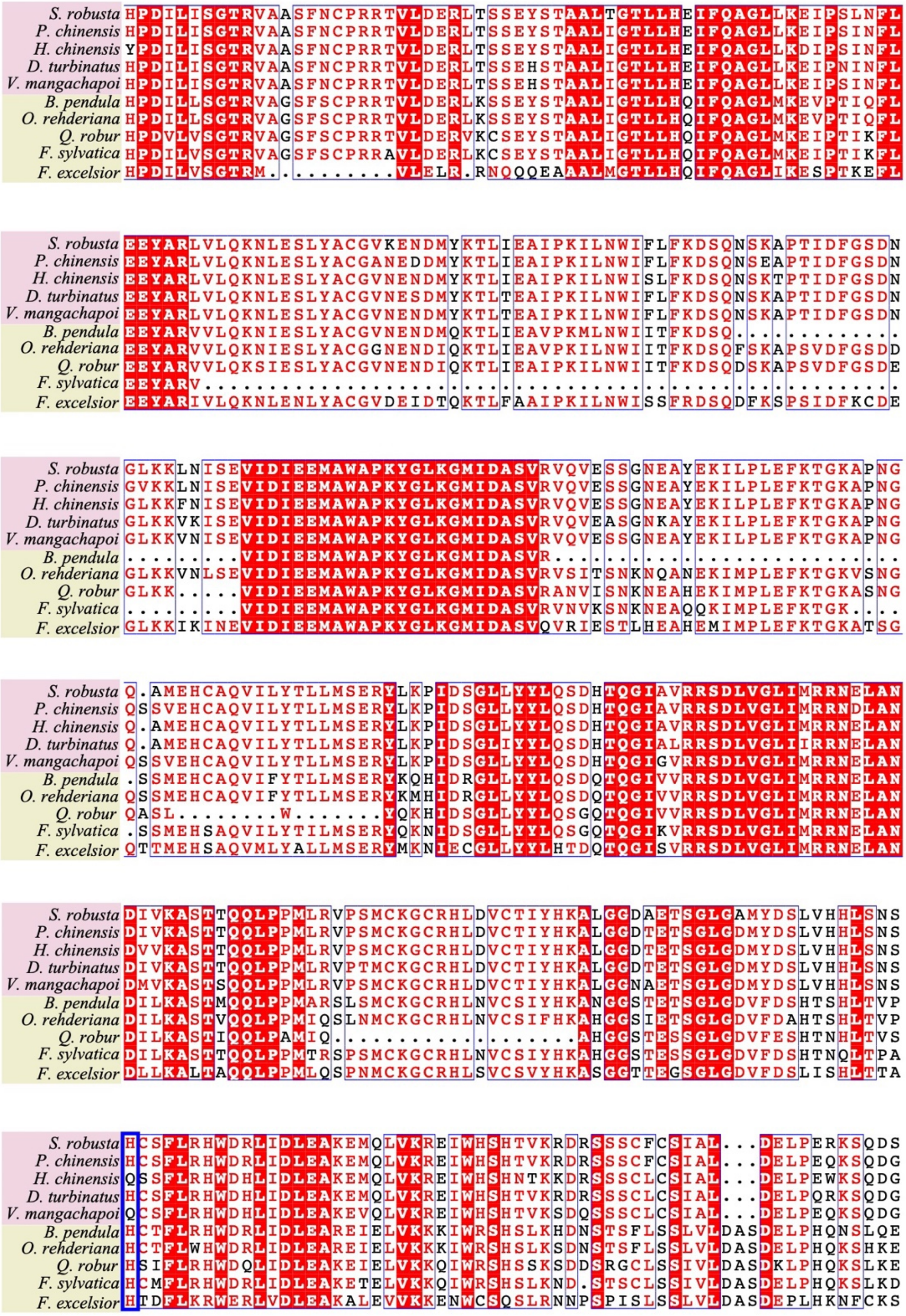

DNA2

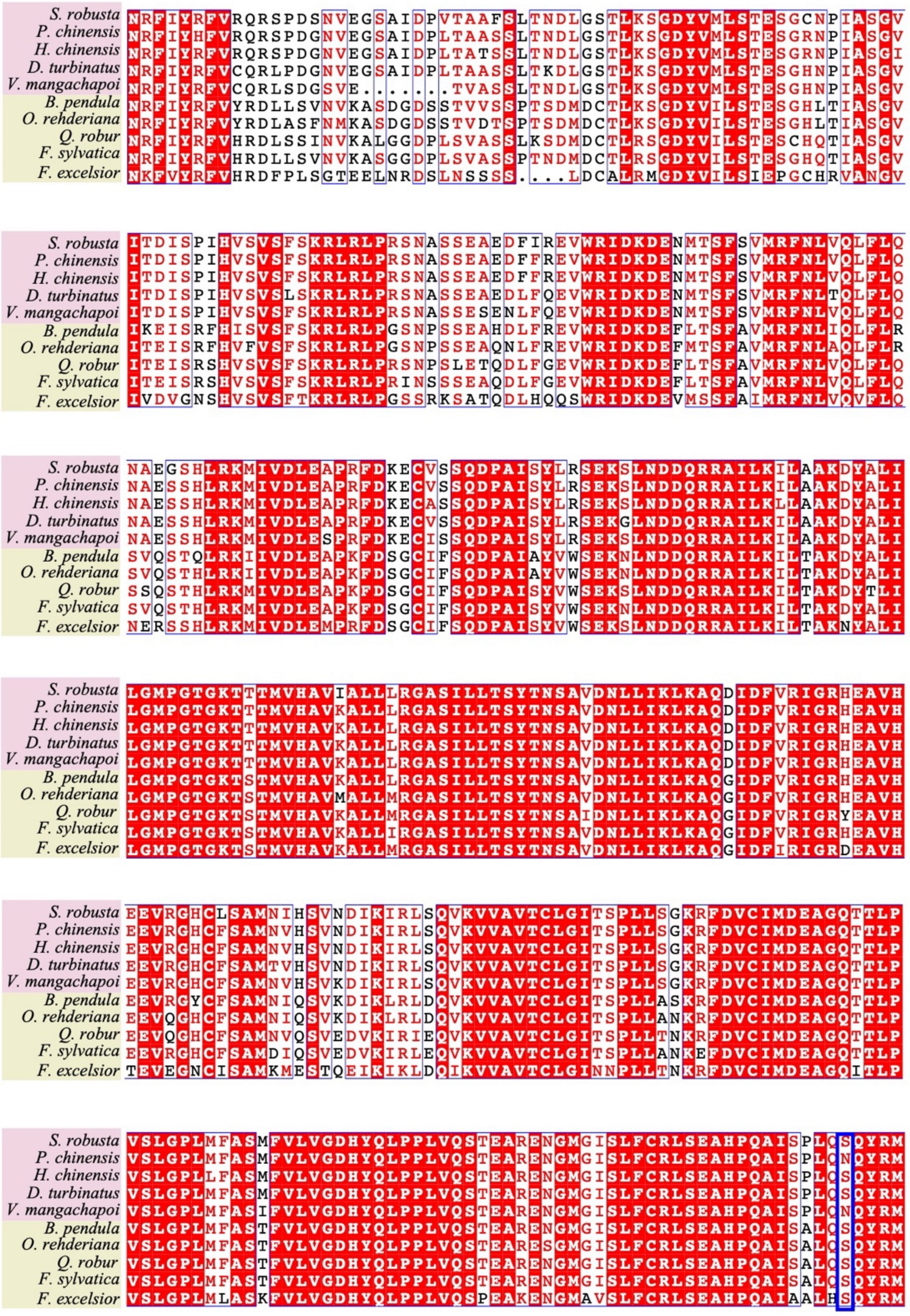
S. robusta NRFIYRFVRQRSPDSNVEGSAIDPVTAAFSLTNDLGSTLKSGDYVMLSTESGCNPIASGV
P. chinensis NRFIYHFVRQRSPDGNVEGSAIDPLTAASSLTNDLGSTLKSGDYVMLSTESGRNPIASGV
H. chinensis NRFIYRFVRQRSPDGNVEGSAIDPLTATSSLTNDLGSTLKSGDYVMLSTESGRNPIASGI
D. turbinatus NRFIYRFVCQRLPDGNVEGSAIDPLTAASSLTKDLGSTLKSGDYVMLSTESGHNPIASGV
V. mangachapoi NRFIYRFVCQRLSDGSVE.......TVASSLTNDLGSTLKSGDYVMLSTESGHNPIASGV
B. pendula NRFIYRFVYRDLLSVNVKASDGDSSTVVSSPTSDMDCTLKSGDYVILSTESGHLTIASGV
O. rehderiana NRFIYRFVYRDLASFNMKASDGDSSTVDTSPTSDMDCTLKSGDYVILSTESGHLTIASGV
Q. robur NRFIYRFVHRDLSSINVKALGGDPLSVASSLKSDMDCTLRSGDYVILSTESCHQTIASGV
F. sylvatica NRFIYRFVHRDLLSVNVKASGGDPLSVASSPTNDMDCTLRSGDYVILSTESGHQTIASGV
F. excelsior NKFVYRFVHRDFPLSGTEELNRDSLNSSSS....LDCALRMGDYVILSIEPGCHRVANGV
S. robusta ITDISPIHVSVSFSKRLRLPRSNASSEAEDFIREVWRIDKDENMTSFSVMRFNLVQLFLQ
P. chinensis ITDISPIHVSVSFSKRLRLPRSNASSEAEDFFREVWRIDKDENMTSFSVMRFNLVQLFLQ
H. chinensis ITDISPIHVSVSFSKRLRLPRSNASSEAEDFFREVWRIDKDENMTSFSVMRFNLVQLFLQ
D. turbinatus ITDISPIHVSVSLSKRLRLPRSNASSEAEDLFQEVWRIDKDENMTSFSVMRFNLTQLFLQ
V. mangachapoi ITDISPIHVSVSFSKRLRLPRSNASSESENLFQEVWRIDKDENMTSFSVMRFNLVQLFLQ
B. pendula IKEISRFHISVSFSKRLRLPGSNPSSEAHDLFREVWRIDKDEFLTSFAVMRFNLIQLFLR
O. rehderiana ITEISRFHVFVSFSKRLRLPGSNPSSEAQNLFREVWRIDKDEFMTSFAVMRFNLAQLFLR
Q. robur ITEISRSHVSVSFSKRLRLPRSNPSLETQDLFGEVWRIDKDEFLTSFAVMRFNLVQLFLQ
F. sylvatica ITEISRSHVSVSFSKRLRLPRINSSSEAQDLFGEVWRIDKDEFLTSFAVMRFNLVQLFLQ
F. excelsior IVDVGNSHVSVSFTKRLRLPGSSRKSATQDLHQQSWRIDKDEVMSSFAIMRFNLVQVFLQ
S. robusta NAEGSHLRKMIVDLEAPRFDKECVSSQDPAISYLRSEKSLNDDQRRAILKILAAKDYALI
P. chinensis NAESSHLRKMIVDLEAPRFDKECVSSQDPAISYLRSEKSLNDDQRRAILKILAAKDYALI
H. chinensis NAESSHLRKMIVDLEAPRFDKECASSQDPAISYLRSEKSLNDDQRRAILKLLAAKDYALI
D. turbinatus NAESSHLRKMIVDLEAPRFDKECVSSQDPAISYLRSEKGLNDDQRRAILKILAAKDYALI
V. mangachapoi NAESSHLRKMIVDLESPRFDKECISSQDPAISYLRSEKSLNDDQRRAILKILAAKDYALI
B. pendula SVQSTQLRKIIVDLEAPKFDSGCIFSQDPAIAYVWSEKNLNDDQRRAILKILTAKDYALI
O. rehderiana SVQSTHLRKIIVDLEAPKFDSGCIFSQDPAIAYVWSEKNLNDDQRRAILKILTAKDYALI
Q. robur SSQSTHLRKMIVDLEAPKFDSGCIFSQDPAISYVWSEKSLNDDQRRAILKILTAKDYTLI
F. sylvatica SVQSTHLRKMIVDLEAPKFDSGCIFSQDPAISYVWSEKNLNDDQRRAILKILTAKDYALI
F. excelsior NERSSHLRKMIVDLEMPRFDSGCIFSQDPAISYVWSEKSLNDDQRRAILKILTAKNYALI
S. robusta LGMPGTGKTTTMVHAVIALLLRGASILLTSYTNSAVDNLLIKLKAQDIDFVRIGRHEAVH
P. chinensis LGMPGTGKTTTMVHAVKALLLRGASILLTSYTNSAVDNLLIKLKAQDIDFVRIGRHEAVH
H. chinensis LGMPGTGKTTTMVHAVKALLLRGASILLTSYTNSAVDNLLIKLKAQDIDFVRIGRHEAVH
D. turbinatus LGMPGTGKTTTMVHAVKALLLRGASILLTSYTNSAVDNLLIKLKAQDIDFVRIGRHEAVH
V. mangachapoi LGMPGTGKTTTMVHAVKALLLRGASILLTSYTNSAVDNLLIKLKAQDIDFVRIGRHEAVH
B. pendula LGMPGTGKTSTMVHAVKALLLRGASILLTSYTNSAVDNLLIKLKAQGIDFVRIGRHEAVH
O. rehderiana LGMPGTGKTSTMVHAVMALLMRGASILLTSYTNSAVDNLLIKLKAQGIDFVRIGRHEAVH
Q. robur LGMPGTGKTSTMVHAVKALLMRGASILLTSYTNSAIDNLLIKLKAQGIDFVRIGRYEAVH
F. sylvatica LGMPGTGKTSTMVHAVKALLIRGASILLTSYTNSAVDNLLIKLKAQGIDFVRIGRHEAVH
F. excelsior LGMPGTGKTSTMVHAVKALLMRGASILLTSYTNSAVDNLLIKLKAQGIDFIRIGRDEAVH
S. robusta EEVRGHCLSAMNIHSVNDIKIRLSQVKVVAVTCLGITSPLLSGKRFDVCIMDEAGQTTLP
P. chinensis EEVRGHCFSAMNVHSVNDIKIRLSQVKVVAVTCLGITSPLLSGKRFDVCIMDEAGQTTLP
H. chinensis EEVRGHCFSAMNVHSVNDIKIRLSQVKVVAVTCLGITSPLLSGKRFDVCIMDEAGQTTLP
D. turbinatus EEVRGHCFSAMTVHSVNDIKIRLSQVKVVAVTCLGITSPLLSGKRFDVCIMDEAGQTTLP
V. mangachapoi EEVRGHCFSAMNVHSVKDIKIRLSQVKVVAVTCLGITSPLLSGKRFDVCIMDEAGQTTLP
B. pendula EEVRGYCFSAMNIQSVKDIKLRLDQVKVVAVTCLGITSPLLASKRFDVCIMDEAGQTTLP
O. rehderiana EEVQGHCFSAMNIQSVKDIKLRLDQVKVVAVTCLGITSPLLANKRFDVCIMDEAGQTTLP
Q. robur EEVQGHCFSAMNVQSVEDVKIRIEQVKVVAVTCLGITSPLLTNKRFDVCIMDEAGQTTLP
F. sylvatica EEVRGHCFSAMDIQSVEDVKIRLEQVKVVAVTCLGITSPLLANKEFDVCIMDEAGQTTLP
F. excelsior TEVEGNCISAMKMESTQEIKIKLDQIKVVAVTCLGINNPLLTNKRFDVCIMDEAGQITLP
S. robusta VSLGPLMFASMFVLVGDHYQLPPLVQSTEARENGMGISLFCRLSEAHPQAISPLQSQYRM
P. chinensis VSLGPLMFASMFVLVGDHYQLPPLVQSTEARENGMGISLFCRLSEAHPQAISPLQNQYRM
H. chinensis VSLGPLLFASMFVLVGDHYQLPPLVQSTEARENGMGISLFCRLSEAHPQAISPLQSQYRM
D. turbinatus VSLGPLMFASMFVLVGDHYQLPPLVQSTEARENGMGISLFCRLSEAHPQAISPLQSQYRM
V. mangachapoi VSLGPLMFASIFVLVGDHYQLPPLVQSTEARENGMGISLFCRLSEAHPQAISPLQNQYRM
B. pendula VSLGPLMFASTFVLVGDHYQLPPLVQSTEARENGMGISLFCRLSEAHPQAISALQSQYRM
O. rehderiana VSLGPLMFASTFVLVGDHYQLPPLVQSTEARESGMGISLFCRLSEAHPQAISALQSQYRM
Q. robur VSLGPLMFASTFVLVGDHYQLPPLVQSTEARENGMGISLFCRLSEAHPQAISALQSQYRM
F. sylvatica VSLGPLMFASTFVLVGDHYQLPPLVQSTEARENGMGISLFCRLSEAHPQAISALQSQYRM
F. excelsior VSLGPLMLASKFVLVGDHYQLPPLVQSPEAKENGMAVSLFCRLSEAHPQAIAALHSQYRM

DNA2

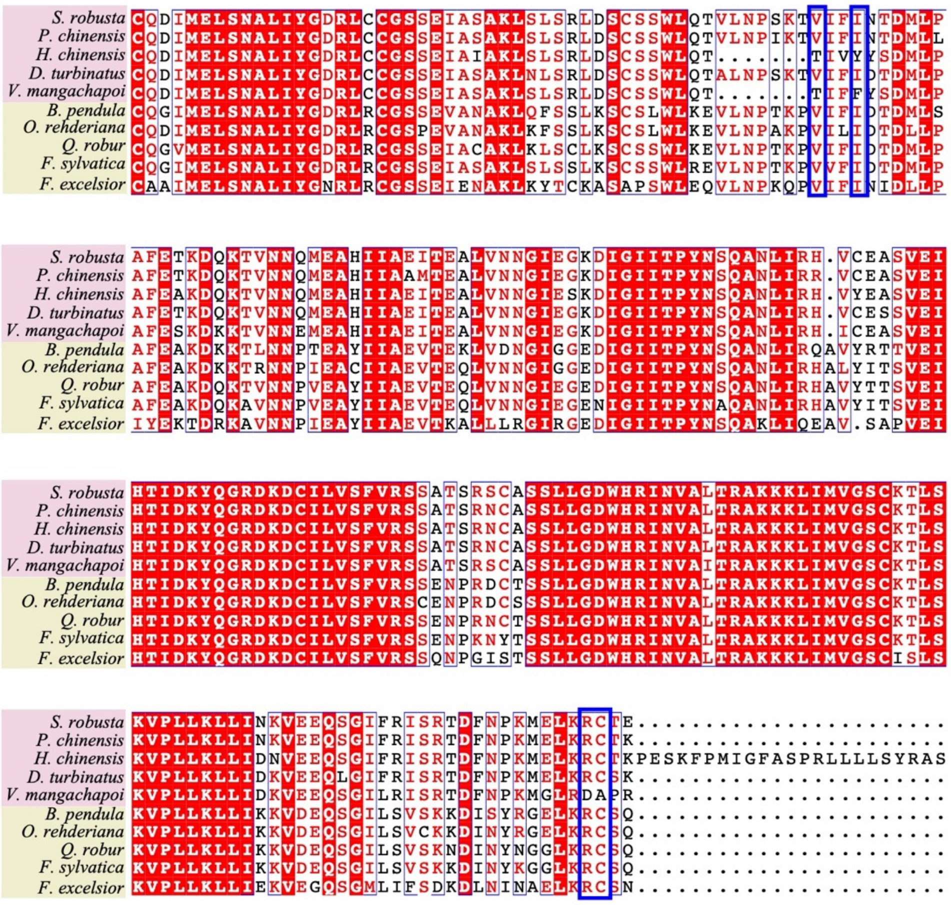
S. robusta CQDIMELSNALIYGDRLCCGSSEIASAKLSLSRLDSCSSWLQTVLNPSKTVIFINTDMLP
P. chinensis CQDIMELSNALIYGDRLCCGSSEIASAKLSLSRLDSCSSWLQTVLNPIKTVIFINTDMLL
H. chinensis CQDIMELSNALIYGDRLCCGSSEIAIAKLSLSRLDSCSSWLQT.......TIVYYSDMLP
D. turbinatus CQDIMELSNALIYGDRLCCGSSEIASAKLNLSRLDSCSSWLQTALNPSKTVIFIDTDMLP
V. mangachapoi CQDIMELSNALIYGDRLCCGSSEIASAKLSLSRLDSCSSWLQT.......TIFFYSDMLP
B. pendula CQGIMELSNALIYGDRLRCGSSEVANAKLQFSSLKSCSLWLKEVLNPTKPVIFIDTDMLS
O. rehderiana CQDIMELSNALIYGDRLRCGSPEVANAKLKFSSLKSCSLWLKEVLNPAKPVILIDTDLLP
Q. robur CQGVMELSNALIYGDRLRCGSSEIACAKLKLSCLKSCSSWLKEVLNPTKPVIFIDTDMLP
F. sylvatica CQGIMELSNALIYGDRLRCGSSEIASAKLSLSSLKSCSSWLREVLNPTKPVVFIDTDMLP
F. excelsior CAAIMELSNALIYGNRLRCGSSEIENAKLKYTCKASAPSWLEQVLNPKQPVIFINIDLLP
S. robusta AFETKDQKTVNNQMEAHIIAEITEALVNNGIEGKDIGIITPYNSQANLIRH.VCEASVEI
P. chinensis AFETKDQKTVNNQMEAHIIAAMTEALVNNGIEGKDIGIITPYNSQANLIRR.VCEASVEI
H. chinensis AFEAKDQKTVNNQMEAHIIAEITEALVNNGIESKDIGIITPYNSQANLIRH.VYEASVEI
D. turbinatus AFETKDQKTVNNQMEAHIIAEITEALVNNGIEGKDIGIITPYNSQANLIRH.VCESSVEI
V. mangachapoi AFESKDKKTVNNEMEAHIIAEITEALVNNGIEGKDIGIITPYNSQANLIRH.ICEASVEI
B. pendula AFEAKDKKTLNNPTEAYIIAEVTEKLVDNGIGGEDIGIITPYNSQANLIRQAVYRTTVEI
O. rehderiana AFEAKDKKTRNNPIEACIIAEVTEQLVNNGIGGEDIGIITPYNSQANLIRHALYITSVEI
Q. robur AFEAKDQKTVNNPVEAYIIAEVTEQLVNNGIEGEDIGIITPYNSQANLIRHAVYTTSVEI
F. sylvatica AFEAKDQKAVNNPVEAYIIAEVTEQLVNNGIEGENIGIITPYNAQANLIRHAVYITSVEI
F. excelsior IYEKTDRKAVNNPIEAYIIAEVTKALLLRGIRGEDIGIITPYNSQAKLIQEAV.SAPVEI
S. robusta HTIDKYQGRDKDCILVSFVRSSATSRSCASSLLGDWHRINVALTRAKKKLIMVGSCKTLS
P. chinensis HTIDKYQGRDKDCILVSFVRSSATSRNCASSLLGDWHRINVALTRAKKKLIMVGSCKTLS
H. chinensis HTIDKYQGRDKDCILVSFVRSSATSRNCASSLLGDWHRINVALTRAKKKLIMVGSCKTLS
D. turbinatus HTIDKYQGRDKDCILVSFVRSSATSRNCASSLLGDWHRINVALTRAKKKLIMVGSCKTLS
V. mangachapoi HTIDKYQGRDKDCILVSFVRSSATSRSCASSLLGDWHRINVAITRAKKKLIMVGSCKTLS
B. pendula HTIDKYQGRDKDCILVSFVRSSENPRDCTSSLLGDWHRINVALTRAKKKLIMVGSCKTLS
O. rehderiana HTIDKYQGRDKDCILVSFVRSCENPRDCSSSLLGDWHRINVALTRAKKKLIMVGSCKTLS
Q. robur HTIDKYQGRDKDCILVSFVRSSENPRNCTSSLLGDWHRINVALTRAKKKLIMVGSCKTLS
F. sylvatica HTIDKYQGRDKDCILVSFVRSSENPKNYTSSLLGDWHRINVALTRAKKKLIMVGSCKTLS
F. excelsior HTIDKYQGRDKDCILVSFVRSSQNPGISTSSLLGDWHRINVALTRAKKKLIMVGSCISLS
S. robusta KVPLLKLLINKVEEQSGIFRISRTDFNPKMELKRCTE......................
P. chinensis KVPLLKLLINKVEEQSGIFRISRTDFNPKMELKRCTK......................
H. chinensis KVPLLKLLIDNVEEQSGIFRISRTDFNPKMELKRCTKPESKFPMIGFASPRLLLLSYRAS
D. turbinatus KVPLLKLLIDKVEEQLGIFRISRTDFNPKMELKRCSK......................
V. mangachapoi KVPLLKLLIDKVEEQSGILRISRTDFNPKMGLRDAPR......................
B. pendula KVPLLKLLIKKVDEQSGILSVSKKDISYRGELKRCSQ......................
O. rehderiana KVPLLKLLIKKVDEQSGILSVCKKDINYRGELKRCSQ......................
Q. robur KVPLLKLLIKKVDEQSGILSVSKNDINYNGGLKRCSQ......................
F. sylvatica KVPLLKLLIKKVDEQSGILSVSKKDINYKGGLKRCSQ......................
F. excelsior KVPLLKLLIEKVEGQSGMLIFSDKDLNINAELKRCSN......................

e cysQ

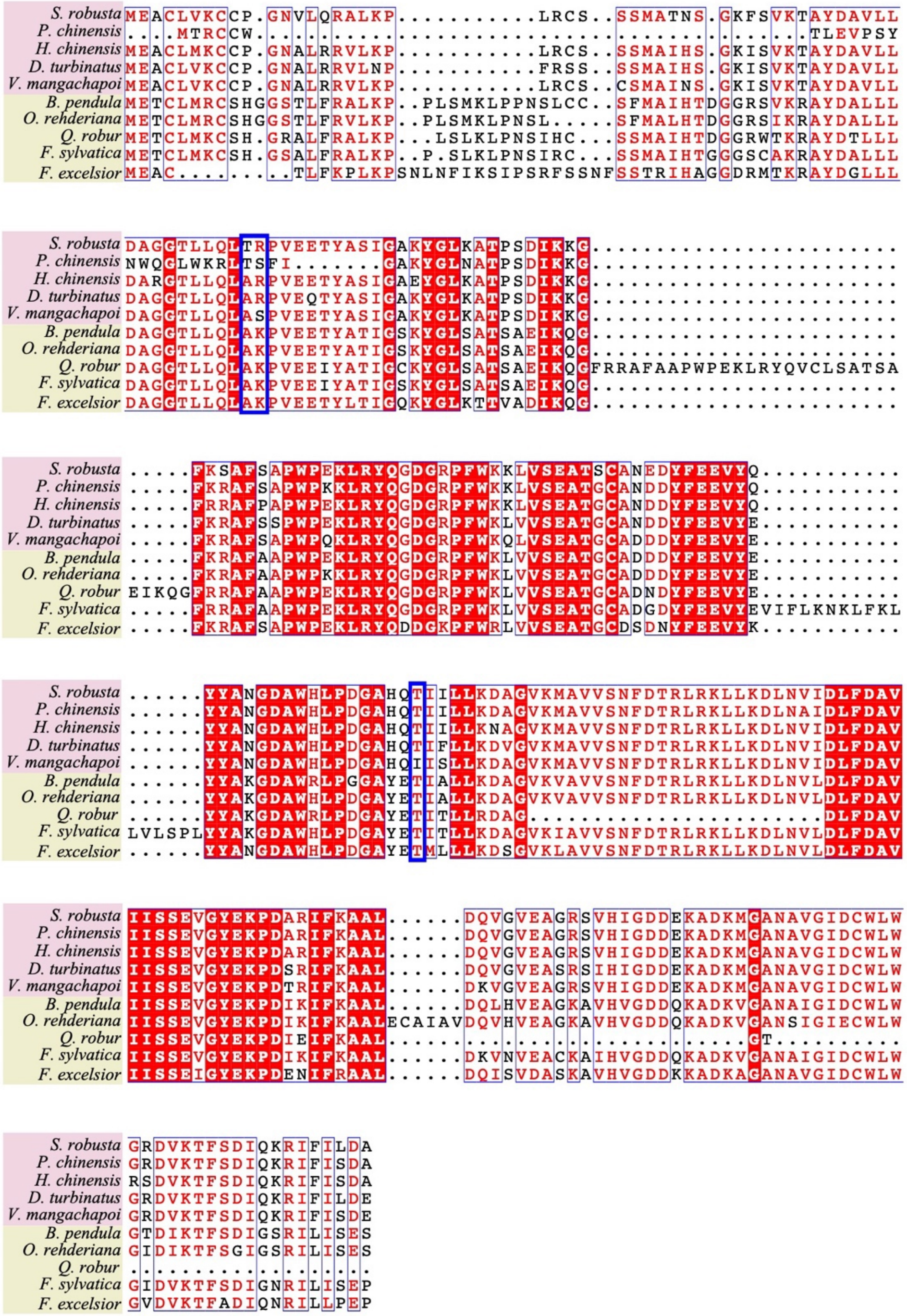

S. robusta MEACLVKCCP.GNVLQRALKP...........LRCS..SSMATNS.GKFSVKTAYDAVLL
P. chinensis ....MTRCCW...TLEVPSY
H. chinensis MEACLMKCCP.GNALRRVLKP...........LRCS..SSMAIHS.GKISVKTAYDAVLL
D. turbinatus MEACLVKCCP.GNALRRVLNP...........FRSS..SSMAIHS.GKISVKTAYDAVLL
V. mangachapoi MEACLVKCCP.GNALRRVLKP...........LRCS..CSMAINS.GKISVKTAYDAVLL
B. pendula METCLMRCSHGGSTLFRALKP..PLSMKLPPNSLCC..SFMAIHTDGGRSVKRAYDALLL
O. rehderiana METCLMRCSHGGSTLFRVLKP..PLSMKLPNSL.....SFMALHTDGGRSIKRAYDALLL
Q. robur METCLMKCSH.GRALFRALKP...LSLKLPNSIHC...SSMAIHTDGGRWTKRAYDTLLL
F. sylvatica METCLMKCSH.GSALFRALKP..P.SLKLPNSIRC...SSMAIHTGGGSCAKRAYDALLL
F. excelsior MEAC.........TLFKPLKPSNLNFIKSIPSRFSSNFSSTRIHAGGDRMTKRAYDGLLL
S. robusta DAGGTLLQLTRPVEETYASIGAKYGLKATPSDIKKG........................
P. chinensis NWQGLWKRLTSFI.......GAKYGLNATPSDIKKG........................
H. chinensis DARGTLLQLARPVEETYASIGAEYGLKATPSDIKKG........................
D. turbinatus DAGGTLLQLARPVEQTYASIGAKYGLKATPSDIKKG........................
V. mangachapoi DAGGTLLQLASPVEETYASIGAKYGLKATPSDIKKG........................
B. pendula DAGGTLLQLAKPVEETYATIGSKYGLSATSAEIKQG........................
O. rehderiana DAGGTLLQLAKPVEETYATIGSKYGLSATSAEIKQG........................
Q. robur DAGGTLLQLAKPVEEIYATIGCKYGLSATSAEIKQGFRRAFAAPWPEKLRYQVCLSATSA
F. sylvatica DAGGTLLQLAKPVEEIYATIGSKYGLSATSAEIKQG........................
F. excelsior DAGGTLLQLAKPVEETYLTIGQKYGLKTTVADIKQG........................
S. robusta .....FKSAFSAPWPEKLRYQGDGRPFWKKLVSEATSCANEDYFEEVYQ...........
P. chinensis .....FKRAFSAPWPKKLRYQGDGRPFWKKLVSEATGCANDDYFEEVYQ...........
H. chinensis .....FRRAFPAPWPEKLRYQGDGRPFWKKLVSEATGCANDDYFEEVYQ...........
D. turbinatus .....FKRAFSSPWPEKLRYQGDGRPFWKLVVSEATGCANDDYFEEVYE...........
V. mangachapoi .....FKRAFSAPWPQKLRYQGDGRPFWKQLVSEATGCADDDYFEEVYE...........
B. pendula .....FKRAFAAPWPEKLRYQGDGRPFWKLVVSEATGCADDDYFEEVYE...........
O. rehderiana .....FKRAFAAPWPKKLRYQGDGRPFWKLVVSEATGCADDDYFEEVYE...........
Q. robur EIKQGFRRAFAAPWPEKLRYQGDGRPFWKLVVSEATGCADNDYFEEVYE...........
F. sylvatica .....FRRAFAAPWPEKLRYQGDGRPFWKLVVSEATGCADGDYFEEVYEVIFLKNKLFKL
F. excelsior .....FKRAFSAPWPEKLRYQDDGKPFWRLVVSEATGCDSDNYFEEVYK...........
S. robusta ......YYANGDAWHLPDGAHQTIILLKDAGVKMAVVSNFDTRLRKLLKDLNVIDLFDAV
P. chinensis ......YYANGDAWHLPDGAHQTIILLKDAGVKMAVVSNFDTRLRKLLKDLNAIDLFDAV
H. chinensis ......YYANGDAWHLPDGAHQTIILLKNAGVKMAVVSNFDTRLRKLLKDLNVIDLFDAV
D. turbinatus ......YYANGDAWHLPDGAHQTIFLLKDVGVKMAVVSNFDTRLRKLLKDLNVIDLFDAV
V. mangachapoi ......YYANGDAWHLPDGAHQIISLLKDAGVKMAVVSNFDTRLRKLLKDLNVIDLFDAV
B. pendula ......YYAKGDAWRLPGGAYETIALLKDAGVKVAVVSNFDTRLRKLLKDLNVLDLFDAV
O. rehderiana ......YYAKGDAWHLPDGAYETIALLKDAGVKVAVVSNFDTRLRKLLKDLNVLDLFDAV
Q. robur ......YYAKGDAWRLPDGAYETITLLRDAG.......................DLFDAV
F. sylvatica LVLSPLYYAKGDAWHLPDGAYETITLLKDAGVKIAVVSNFDTRLRKLLKDLNVLDLFDAV
F. excelsior ......YYANGDAWHLPDGAYETMLLLKDSGVKLAVVSNFDTRLRKLLKDLNVLDLFDAV
S. robusta IISSEVGYEKPDARIFKAAL......DQVGVEAGRSVHIGDDEKADKMGANAVGIDCWLW
P. chinensis IISSEVGYEKPDARIFKAAL......DQVGVEAGRSVHIGDDEKADKMGANAVGIDCWLW
H. chinensis IISSEVGYEKPDARIFKAAL......DQVGVEAGRSVHIGDDEKADKMGANAVGIDCWLW
D. turbinatus IISSEVGYEKPDSRIFKAAL......DQVGVEASRSIHIGDDEKADKMGANAVGIDCWLW
V. mangachapoi IISSEVGYEKPDTRIFKAAL......DKVGVEAGRSVHIGDDEKADKMGANAVGIDCWLW
B. pendula IISSEVGYEKPDIKIFKAAL......DQLHVEAGKAVHVGDDQKADKVGANAIGIDCWLW
O. rehderiana IISSEVGYEKPDIKIFKAALECAIAVDQVHVEAGKAVHVGDDQKADKVGANSIGIECWLW
Q. robur IISSEVGYEKPDIEIFKAAL............................GT..........
F. sylvatica IISSEVGYEKPDIKIFKAAL......DKVNVEACKAIHVGDDQKADKVGANAIGIDCWLW
F. excelsior IISSEIGYEKPDENIFRAAL......DQISVDASKAVHVGDDKKADKAGANAVGIDCWLW
S. robusta GRDVKTFSDIQKRIFILDA
P. chinensis GRDVKTFSDIQKRIFISDA
H. chinensis RSDVKTFSDIQKRIFISDA
D. turbinatus GRDVKTFSDIQKRIFILDE
V. mangachapoi GRDVKTFSDIQKRIFISDE
B. pendula GTDIKTFSDIGSRILISES
O. rehderiana GIDIKTFSGIGSRILISES
Q. robur ....
F. sylvatica GIDVKTFSDIGNRILISEP
F. excelsior GVDVKTFADIQNRILLPEP

f BLM

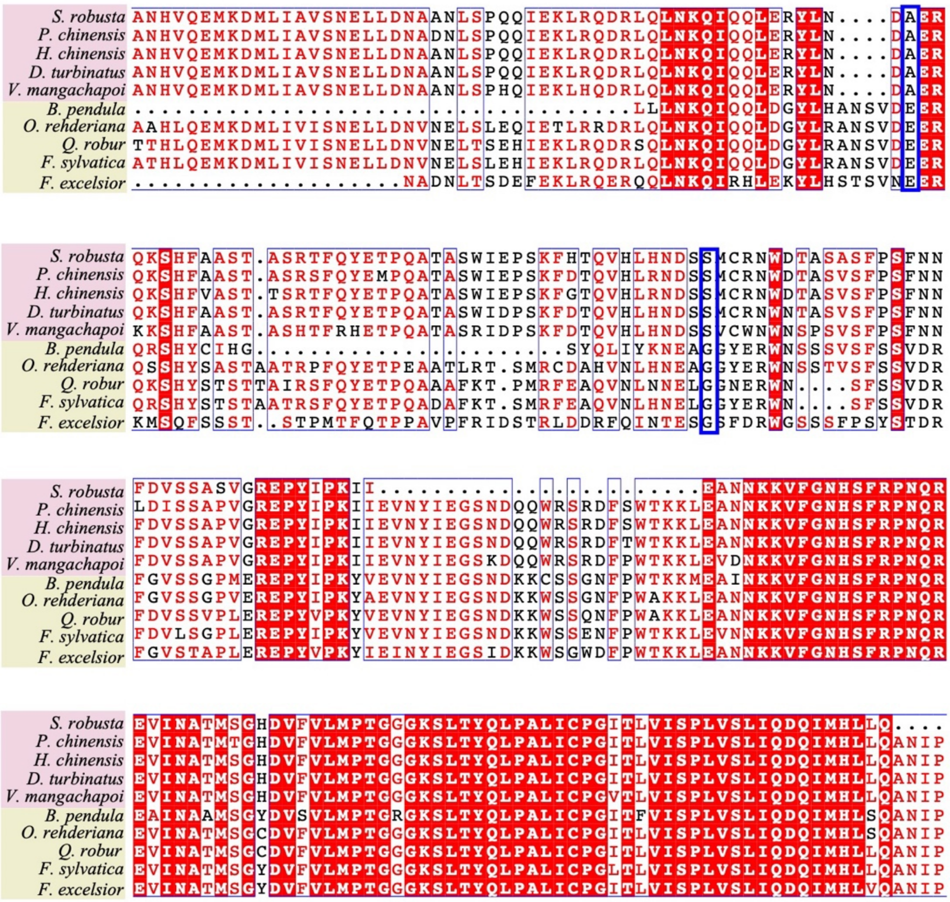
S. robusta
P. chinensis
H. chinensis
D. turbinatus
V. mangachapoi
B. pendula
O. rehderiana
Q. robur
F. sylvatica
F. excelsior

g LRPPRC

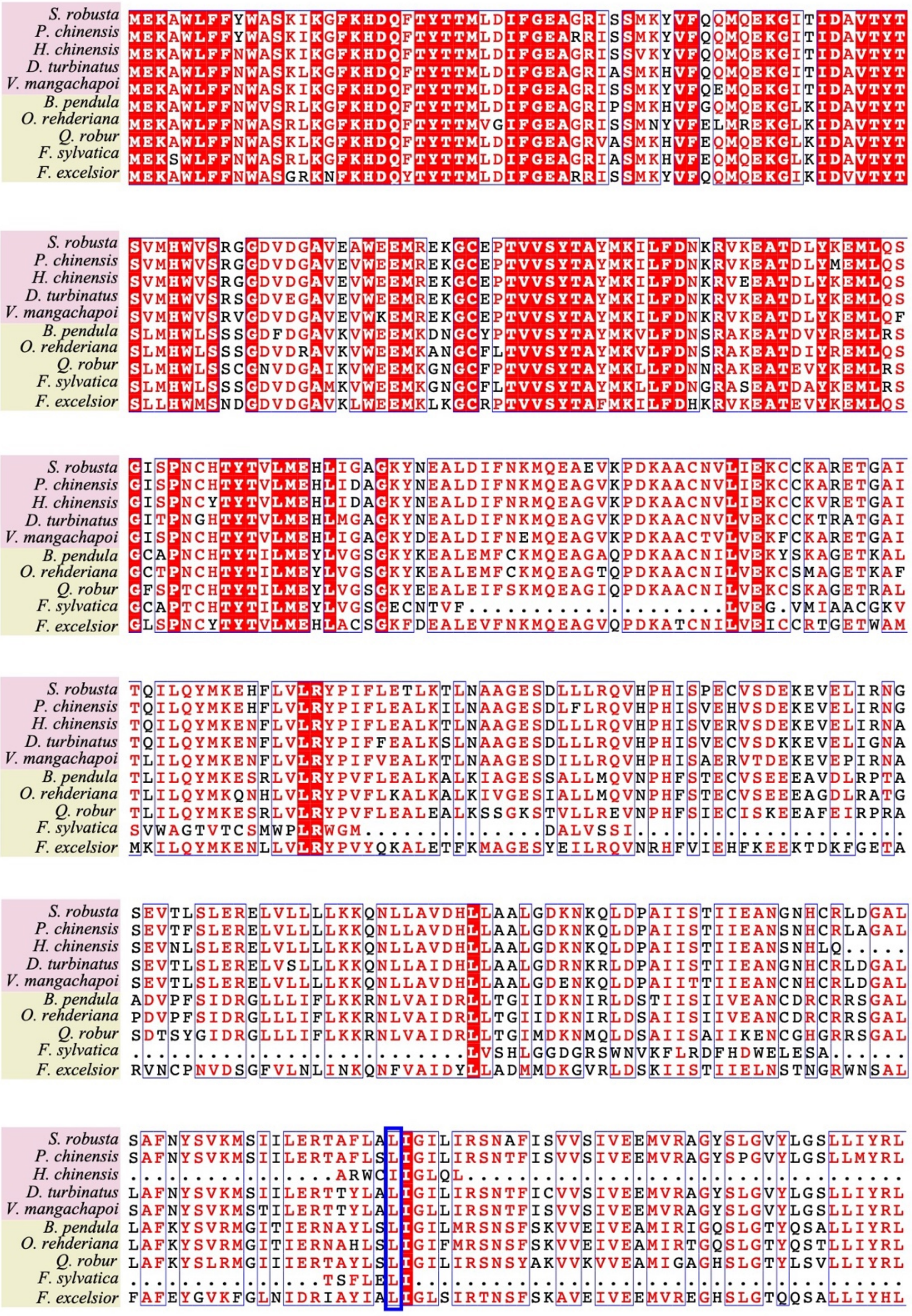
S. robusta
P. chinensis
H. chinensis
D. turbinatus
V. mangachapoi
B. pendula
O. rehderiana
Q. robur
F. sylvatica
F. excelsior

h LSD1

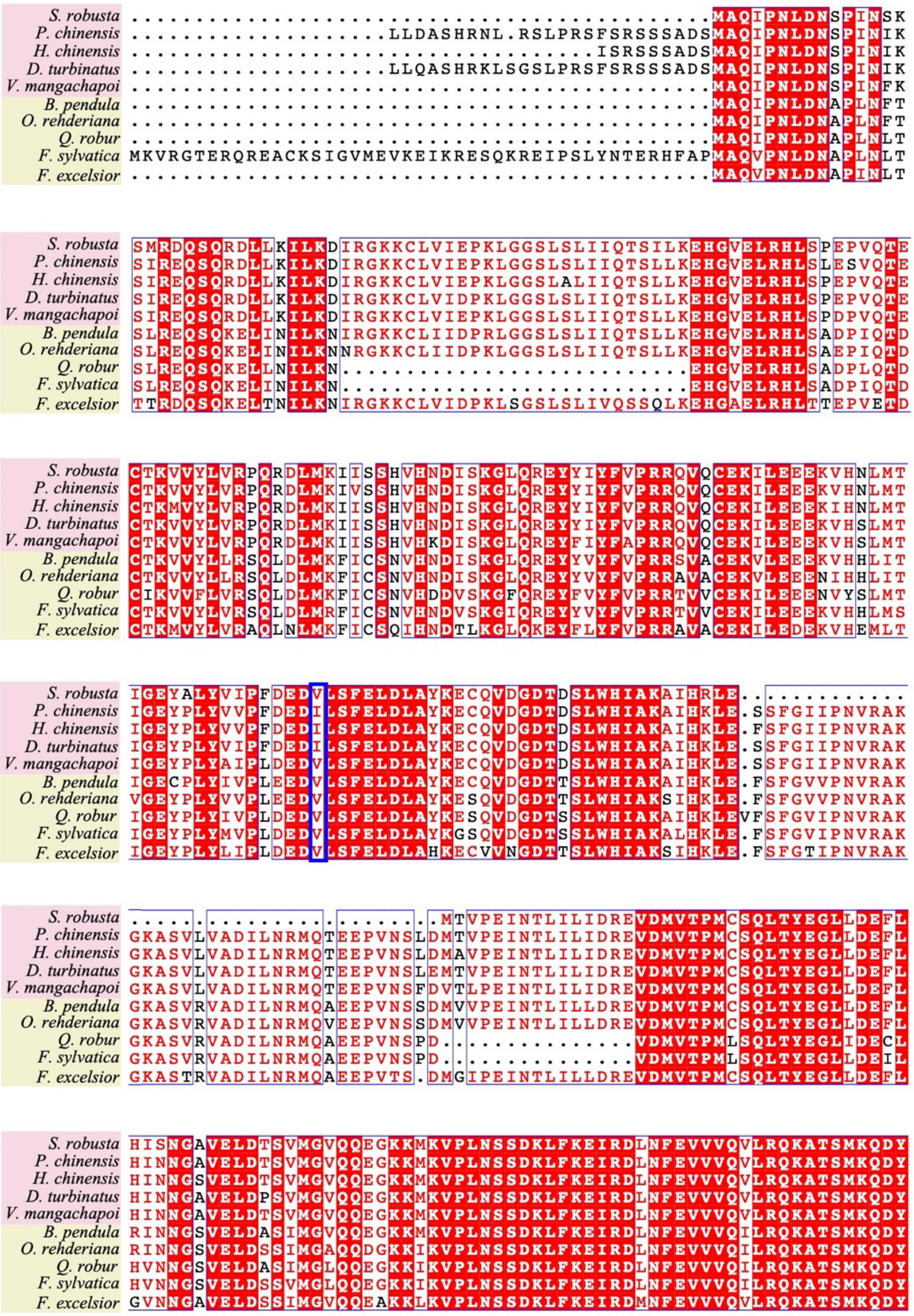
S. robusta
P. chinensis
H. chinensis
D. turbinatus
V. mangachapoi
B. pendula
O. rehderiana
Q. robur
F. sylvatica
F. excelsior

LSD1

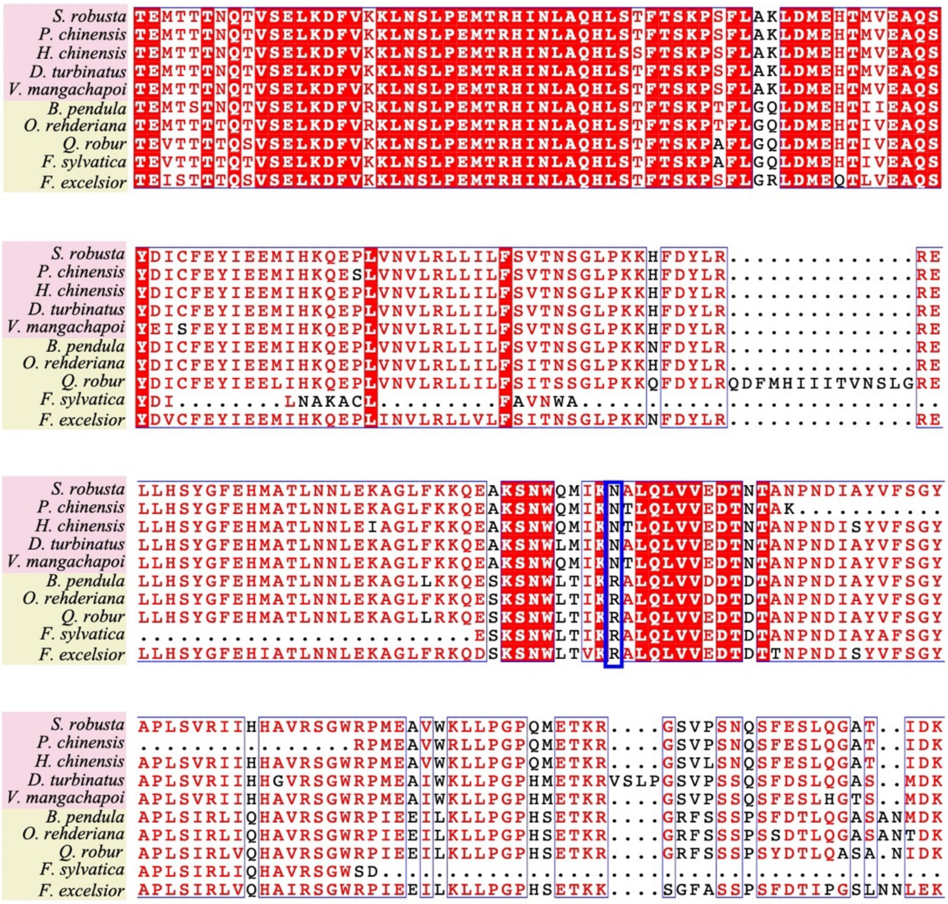
S. robusta
P. chinensis
H. chinensis
D. turbinatus
V. mangachapoi
B. pendula
O. rehderiana
Q. robur
F. sylvatica
F. excelsior

i SMG1

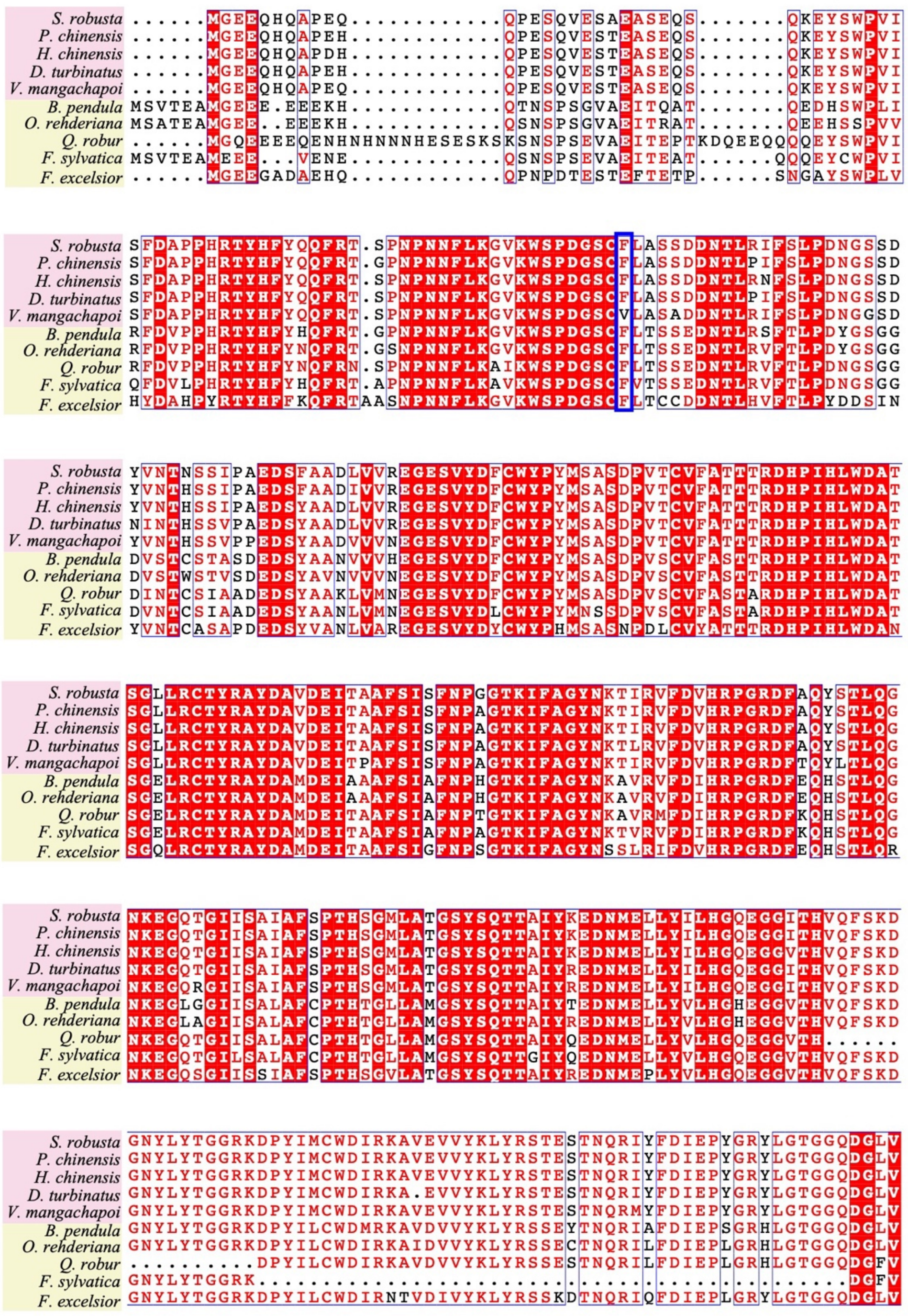
S. robusta
P. chinensis
H. chinensis
D. turbinatus
V. mangachapoi
B. pendula
O. rehderiana
Q. robur
F. sylvatica
F. excelsior

SMG1

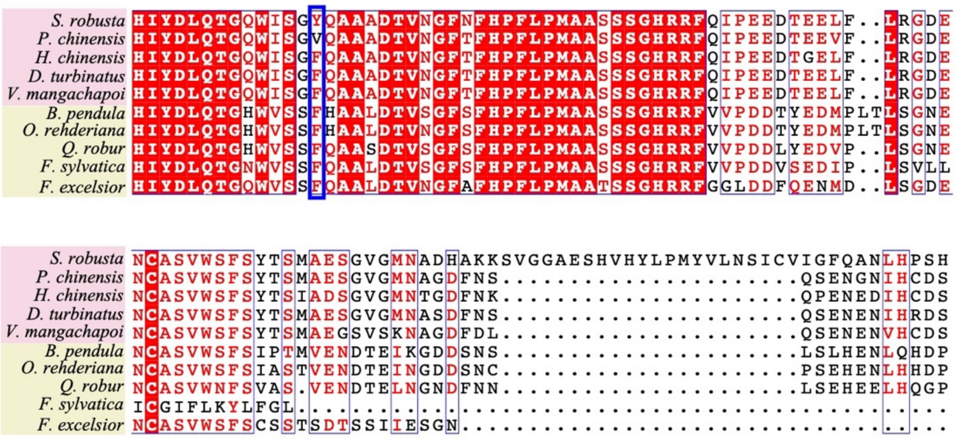

S. robusta HIYDLQTGQWISGYQAAADTVNGFNFHPFLPMAASSSGHRRFQIPEEDTEELF..LRGDE
P. chinensis HIYDLQTGQWISGVQAAADTVNGFTFHPFLPMAASSSGHRRFQIPEEDTEEVF..LRGDE
H. chinensis HIYDLQTGQWISGFQAAADTVNGFTFHPFLPMAASSSGHRRFQIPEEDTGELF..LRGDE
D. turbinatus HIYDLQTGQWISGFQAAADTVNGFTFHPFLPMAASSSGHRRFQIPEEDTEELF..LRGDE
V. mangachapoi HIYDLQTGQWISGFQAAADTVNGFTFHPFLPMAASSSGHRRFQIPEEDTEELF..LRGDE
B. pendula HIYDLQTGHWVSSFHAALDTVSGFSFHPFLPMAASSSGHRRFVVPDDTYEDMPLTLSGNE
O. rehderiana HIYDLQTGHWVSSFHAALDTVNGFSFHPFLPMAASSSGHRRFVVPDDTYEDMPLTLSGNE
Q. robur HIYDLQTGHWVSSFQAASDTVSGFSFHPFLPMAASSSGHRRFVVPDDLYEDVP..LSGNE
F. sylvatica HIYDLQTGNWVSSFQAALDTVSGFSFHPFLPMAASSSGHRRFVVPDDVSEDIP..LSVLL
F. excelsior HIYDLQTGQWVSSFQAALDTVNGFAFHPFLPMAATSSGHRRFGGLDDFQENMD..LSGDE
S. robusta NCASVWSFSYTSMAESGVGMNADHAKKSVGGAESHVHYLPMYVLNSICVIGFQANLHPSH
P. chinensis NCASVWSFSYTSMAESGVGMNAGDFNS......................QSENGNIHCDS
H. chinensis NCASVWSFSYTSIADSGVGMNTGDFNK......................QPENEDIHCDS
D. turbinatus NCASVWSFSYTSMAESGVGMNASDFNS......................QSENENIHRDS
V. mangachapoi NCASVWSFSYTSMAEGSVSKNAGDFDL......................QSENENVHCDS
B. pendula NCASVWSFSIPTMVENDTEIKGDDSNS......................LSLHENLQHDP
O. rehderiana NCASVWSFSIASTVENDTEINGDDSNC......................PSEHENLHHDP
Q. robur NCASVWNFSVAS.VENDTELNGNDFNN......................LSEHEELHQGP
F. sylvatica ICGIFLKYLFGL..
F. excelsior NCASVWSFSCSSTSDTSSIIESGN....................................

j PPP2R5

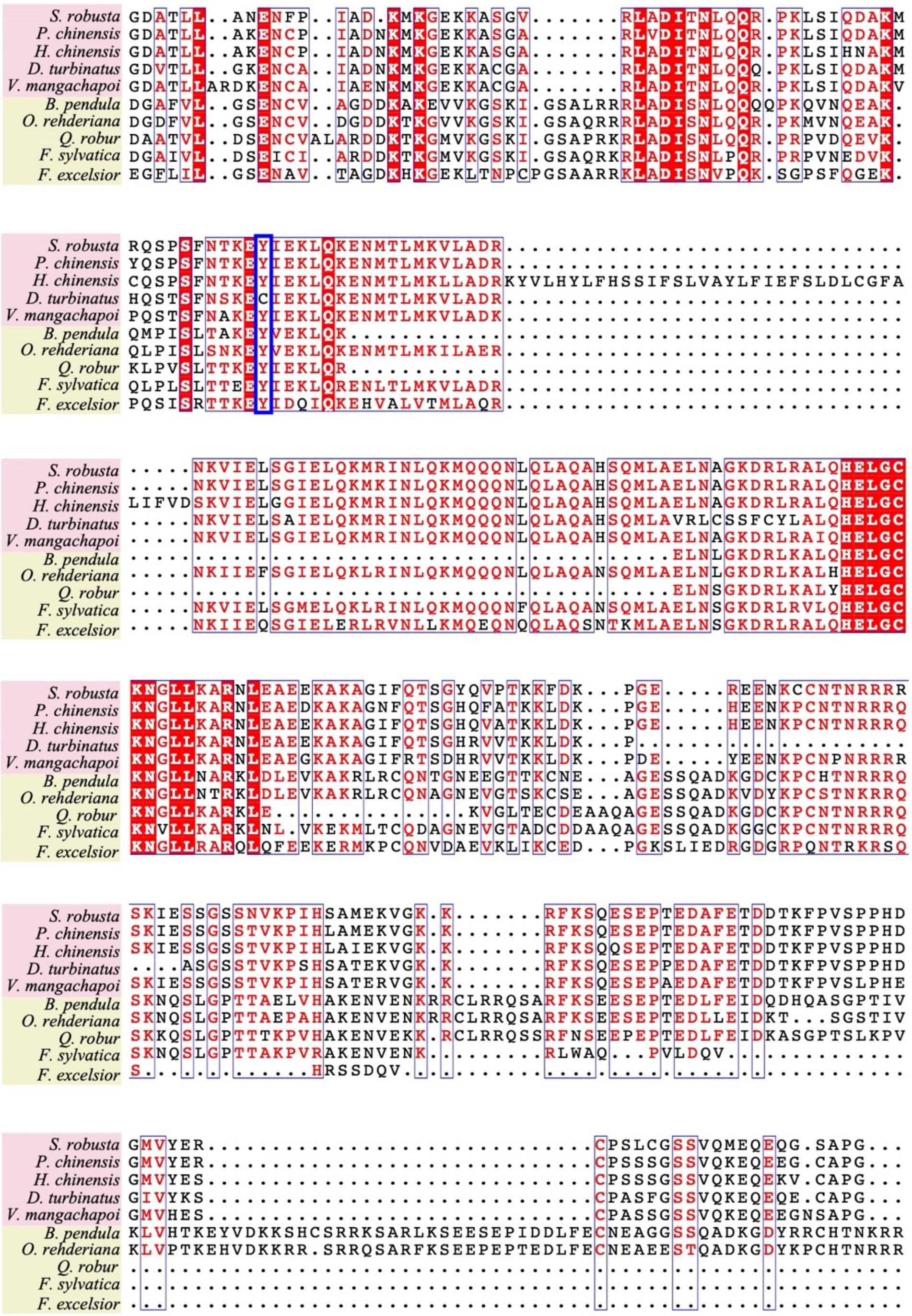

S. robusta GDATLL..ANENFP..IAD.KMKGEKKASGV.......RLADITNLQQR.PKLSIQDAKM
P. chinensis GDATLL..AKENCP..IADNKMKGEKKASGA.......RLVDITNLQQR.PKLSIQDAKM
H. chinensis GDATLL..AKENCP..IADNKMKGEKKASGA.......RLADITNLQQR.PKLSIHNAKM
D. turbinatus GDVTLL..GKENCA..IADNKMKGEKKACGA.......RLADITNLQQQ.PKLSIQDAKM
V. mangachapoi GDATLLARDKENCA..IAENKMKGEKKACGA.......RLADITNLQQR.PKLSIQDAKV
B. pendula DGAFVL..GSENCV..AGDDKAKEVVKGSKI.GSALRRRLADISNLQQQQPKQVNQEAK.
O. rehderiana DGDFVL..GSENCV..DGDDKTKGVVKGSKI.GSAQRRRLADISNLQQR.PKMVNQEAK.
Q. robur DAATVL..DSENCVALARDDKTKGMVKGSKI.GSAPRKRLADISNLQQR.PRPVDQEVK.
F. sylvatica DGAIVL..DSEICI..ARDDKTKGMVKGSKI.GSAQRKRLADISNLPQR.PRPVNEDVK.
F. excelsior EGFLIL..GSENAV..TAGDKHKGEKLTNPCPGSAARRKLADISNVPQK.SGPSFQGEK.

S. robusta RQSPSFNTKEYIEKLQKENMTLMKVLADR...............................
P. chinensis YQSPSFNTKEYIEKLQKENMTLMKVLADR...............................
H. chinensis CQSPSFNTKEYIEKLQKENMTLMKLLADRKYVLHYLFHSSIFSLVAYLFIEFSLDLCGFA
D. turbinatus HQSTSFNSKECIEKLQKENMTLMKVLADR...............................
V. mangachapoi PQSTSFNAKEYIEKLQKENMTLMKVLADK...............................
B. pendula QMPISLTAKEYVEKLQK...
O. rehderiana QLPISLSNKEYVEKLQKENMTLMKILAER...............................
Q. robur KLPVSLTTKEYIEKLQR...
F. sylvatica QLPLSLTTEEYIEKLQRENLTLMKVLADR...............................
F. excelsior PQSISRTTKEYIDQIQKEHVALVTMLAQR...............................

S. robusta .....NKVIELSGIELQKMRINLQKMQQQNLQLAQAHSQMLAELNAGKDRLRALQHELGC
P. chinensis .....NKVIELSGIELQKMKINLQKMQQQNLQLAQAHSQMLAELNAGKDRLRALQHELGC
H. chinensis LIFVDSKVIELGGIELQKMRINLQKMQQQNLQLAQAHSQMLAELNAGKDRLRALQHELGC
D. turbinatus .....NKVIELSAIELQKMRINLQKMQQQNLQLAQAHSQMLAVRLCSSFCYLALQHELGC
V. mangachapoi .....NKVIELSGIELQKMRINLQKMQQQNLQLAQAHSQMLAELNAGKDRLRAIQHELGC
B. pendula ....ELNLGKDRLKALQHELGC
O. rehderiana .....NKIIEFSGIELQKLRINLQKMQQQNLQLAQANSQMLAELNLGKDRLKALHHELGC
Q. robur ....ELNSGKDRLKALYHELGC
F. sylvatica .....NKVIELSGMELQKLRINLQKMQQQNFQLAQANSQMLAELNSGKDRLRVLQHELGC
F. excelsior .....NKIIEQSGIELERLRVNLLKMQEQNQQLAQSNTKMLAELNSGKDRLRALQHELGC

S. robusta KNGLLKARNLEAEEKAKAGIFQTSGYQVPTKKFDK...PGE.....REENKCCNTNRRRR
P. chinensis KNGLLKARNLEAEDKAKAGNFQTSGHQFATKKLDK...PGE.....HEENKPCNTNRRRQ
H. chinensis KNGLLKARNLEAEEKAKAGIFQTSGHQVATKKLDK...PGE.....HEENKPCNTNRRRQ
D. turbinatus KNGLLKARNLEAEEKAKAGIFQTSGHRVVTKKLDK...P.....................
V. mangachapoi KNGLLKARNLEAEGKAKAGIFRTSDHRVVTKKLDK...PDE.....YEENKPCNPNRRRR
B. pendula KNGLLNARKLDLEVKAKRLRCQNTGNEEGTTKCNE...AGESSQADKGDCKPCHTNRRRQ
O. rehderiana KNGLLNTRKLDLEVKAKRLRCQNAGNEVGTSKCSE...AGESSQADKVDYKPCSTNKRRQ
Q. robur KNGLLKARKLE...............KVGLTECDEAAQAGESSQADKGDCKPCNTNRRRQ
F. sylvatica KNVLLKARKLNL.VKEKMLTCQDAGNEVGTADCDDAAQAGESSQADKGGCKPCNTNRRRQ
F. excelsior KNGLLRARQLQFEEKERMKPCQNVDAEVKLIKCED...PGKSLIEDRGDGRPQNTRKRSQ

S. robusta SKIESSGSSNVKPIHSAMEKVGK.K.......RFKSQESEPTEDAFETDDTKFPVSPPHD
P. chinensis SKIESSGSSTVKPIHLAMEKVGK.K.......RFKSQESEPTEDAFETDDTKFPVSPPHD
H. chinensis SKIESSGSSTVKPIHLAIEKVGK.K.......RFKSQQSEPTEDAFETDDTKFPVSPPHD
D. turbinatus ....ASGSSTVKPSHSATEKVGK.K.......RFKSQESEPPEDAFETDDTKFPVSPPHD
V. mangachapoi SKIESSGSSTVKPIHSATERVGK.K.......RFKSQESEPAEDAFETDDTKFPVSLPHE
B. pendula SKNQSLGPTTAELVHAKENVENKRRCLRRQSARFKSEESEPTEDLFEIDQDHQASGPTIV
O. rehderiana SKNQSLGPTTAEPAHAKENVENKRRCLRRQSARFKSEESEPTEDLLEIDKT...SGSTIV
Q. robur SKKQSLGPTTTKPVHAKENVEKK.RCLRRQSSRFNSEEPEPTEDLFEIDKASGPTSLKPV
F. sylvatica SKNQSLGPTTAKPVRAKENVENK.........RLWAQ...PVLDQV..............
F. excelsior S.............HRSSDQV.......................................

S. robusta GMVYER..............................CPSLCGSSVQMEQEQG.SAPG...
P. chinensis GMVYER..............................CPSSSGSSVQKEQEEG.CAPG...
H. chinensis GMVYES..............................CPSSSGSSVQKEQEKV.CAPG...
D. turbinatus GIVYKS..............................CPASFGSSVQKEQEQE.CAPG...
V. mangachapoi GMVHES..............................CPASSGSSVQKEQEEGNSAPG...
B. pendula KLVHTKEYVDKKSHCSRRKSARLKSEESEPIDDLFECNEAGGSSQADKGDYRRCHTNKRR
O. rehderiana KLVPTKEHVDKKRR.SRRQSARFKSEEPEPTEDLFECNEAEESTQADKGDYKPCHTNRRR
Q. robur ....
F. sylvatica ....
F. excelsior ....

PPP2R5

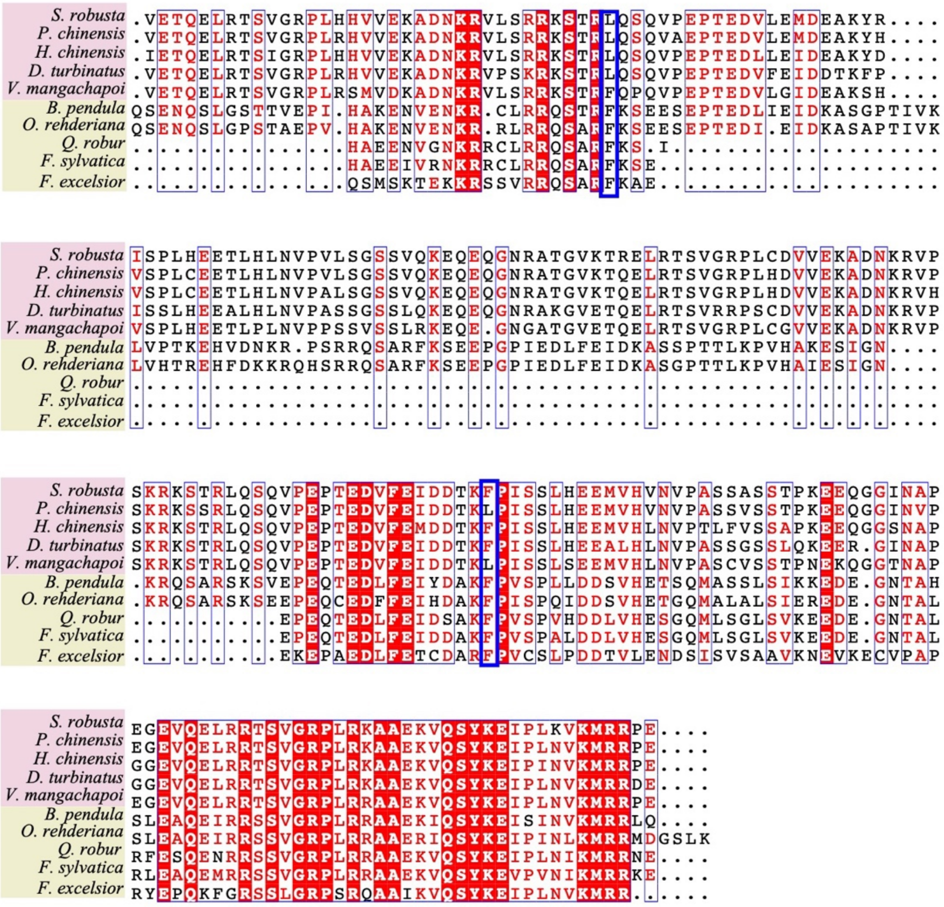

S. robusta
P. chinensis
H. chinensis
D. turbinatus
V. mangachapoi
B. pendula
O. rehderiana
Q. robur
F. sylvatica
F. excelsior

k SKI3

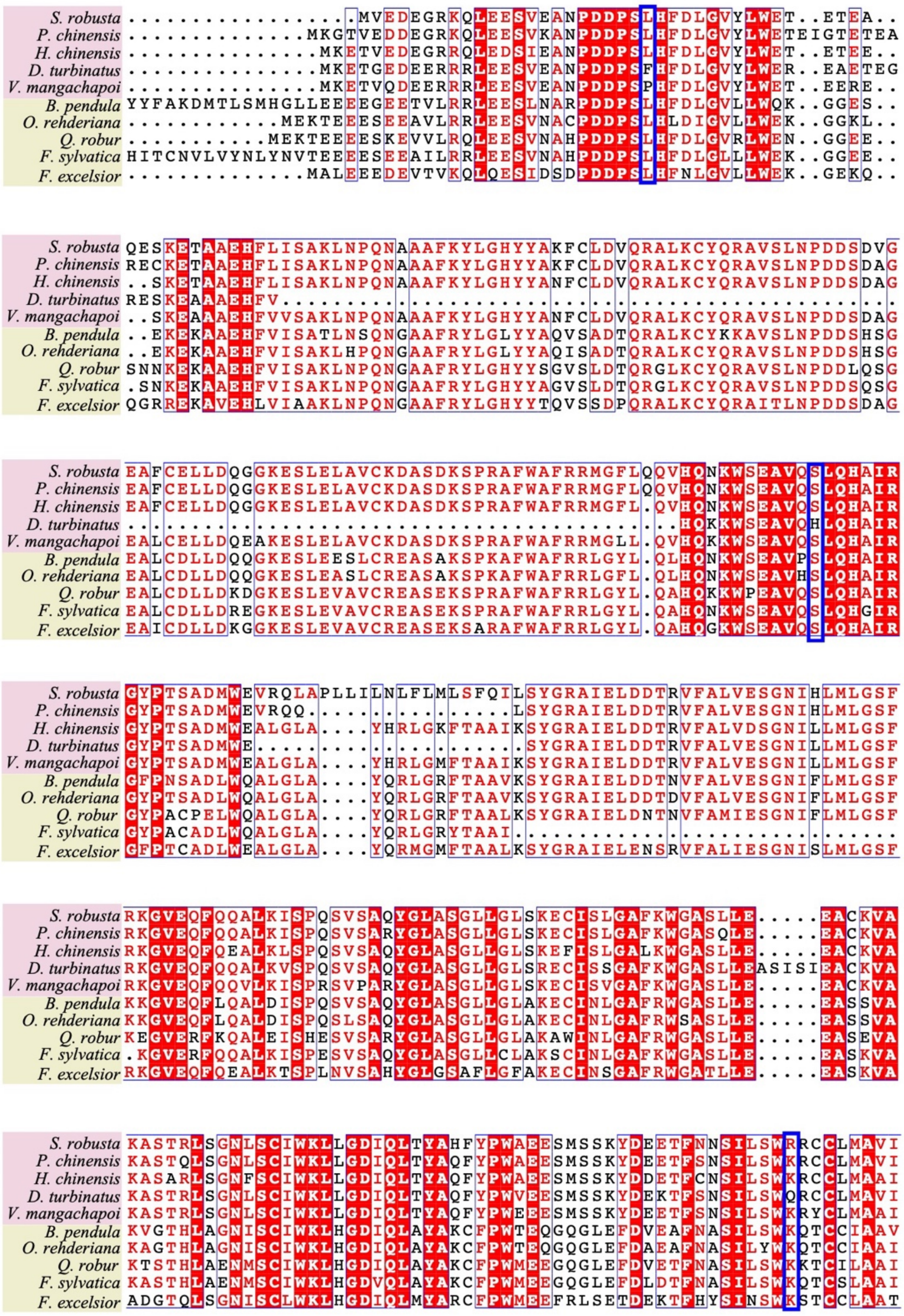

S. robusta .................MVEDEGRKQLEESVEANPDDPSLHFDLGVYLWET..ETEA..
P. chinensis ..............MKGTVEDDEGRKQLEESVKANPDDPSLHFDLGVYLWETEIGTETEA
H. chinensis ..............MKETVEDEGRKQLEDSIEANPDDPSLHFDLGVYLWET..ETEE..
D. turbinatus ..............MKETGEDEERRRLEESVEANPDDPSFHFDLGVYLWER..EAETEG
V. mangachapoi ..............MKETVQDEERRRLEESVEANPDDPSPHFDLGVYLWET..EERE..
B. pendula YYFAKDMTLSMHGLLEEEEGEETVLRRLEESLNARPDDPSLHFDLGVLLWQK..GGES..
O. rehderiana ...........MEKTEEEESEEAVLRRLEESVNACPDDPSLHLDIGVLLWEK..GGKL..
Q. robur ..........MEKTEEEESKEVVLRQLEESVNAHPDDPSLHFDLGVRLWEN..GGEE..
F. sylvatica HITCNVLVYNLYNVTEEEESEEAILRRLEESVNAHPDDPSLHFDLGLLLWEK..GGEE..
F. excelsior ..............MALEEEDEVTVKQLQESIDSDPDDPSLHFNLGVLLWEK..GEKQ..
S. robusta QESKETAAEHFLISAKLNPQNAAAFKYLGHYYAKFCLDVQRALKCYQRAVSLNPDDSDVG
P. chinensis RECKETAAEHFLISAKLNPQNAAAFKYLGHYYAKFCLDVQRALKCYQRAVSLNPDDSDAG
H. chinensis ..SKETAAEHFLISAKLNPQNAAAFKYLGHYYANFCLDVQRALKCYQRAVSLNPDDSDAG
D. turbinatus RESKEAAAEHFV..
V. mangachapoi ..SKEAAAEHFVVSAKLNPQNAAAFKYLGHYYANFCLDVQRALKCYQRAVSLNPDDSDAG
B. pendula ..EKEKAAEHFVISATLNSQNGAAFRYLGLYYAQVSADTQRALKCYKKAVSLNPDDSHSG
O. rehderiana ..EKEKAAEHFVISAKLHPQNGAAFRYLGLYYAQISADTQRALKCYQRAVSLNPDDSHSG
Q. robur SNNKEKAAEHFVISAKLNPQNGAAFRYLGHYYSGVSLDTQRGLKCYQRAVSLNPDDLQSG
F. sylvatica .SNKEKAAEHFVISAKLNPQNGAAFRYLGHYYAGVSLDTQRGLKCYQRAVSLNPDDSQSG
F. excelsior QGRREKAVEHLVIAAKLNPQNGAAFRYLGHYYTQVSSDPQRALKCYQRAITLNPDDSDAG
S. robusta EAFCELLDQGGKESLELAVCKDASDKSPRAFWAFRRMGFLQQVHQNKWSEAVQSLQHAIR
P. chinensis EAFCELLDQGGKESLELAVCKDASDKSPRAFWAFRRMGFLQQVHQNKWSEAVQSLQHAIR
H. chinensis EAFCELLDQGGKESLELAVCKDASDKSPRAFWAFRRMGFL.QVHQNKWSEAVQSLQHAIR
D. turbinatus ....HQKKWSEAVQHLQHAIR
V. mangachapoi EALCELLDQEAKESLELAVCKDASDKSPRAFWAFRRMGLL.QVHQKKWSEAVQSLQHAIR
B. pendula EALCDLLDQQGKESLEESLCREASAKSPKAFWAFRRLGYL.QLHQNKWSEAVPSLQHAIR
O. rehderiana EALCDLLDQQGKESLEASLCREASAKSPKAFWAFRRLGFL.QLHQNKWSEAVHSLQHAIR
Q. robur EALCDLLDKDGKESLEVAVCREASEKSPRAFWAFRRLGYL.QAHQKKWPEAVQSLQHAIR
F. sylvatica EALCDLLDREGKESLEVAVCREASEKSPRAFWAFRRLGYL.QAHQNKWSEAVQSLQHGIR
F. excelsior EAICDLLDKGGKESLEVAVCREASEKSARAFWAFRRLGYL.QAHQGKWSEAVQSLQHAIR
S. robusta GYPTSADMWEVRQLAPLLILNLFLMLSFQILSYGRAIELDDTRVFALVESGNIHLMLGSF
P. chinensis GYPTSADMWEVRQQ..............LSYGRAIELDDTRVFALVESGNIHLMLGSF
H. chinensis GYPTSADMWEALGLA....YHRLGKFTAAIKSYGRAIELDDTRVFALVDSGNILLMLGSF
D. turbinatus GYPTSADMWE.................SYGRAIELDDTRVFALVESGNILLMLGSF
V. mangachapoi GYPTSADMWEALGLA....YHRLGMFTAAIKSYGRAIELDDTRVFALVESGNILLMLGSF
B. pendula GFPNSADLWQALGLA....YQRLGRFTAAVKSYGRAIELDDTNVFALVESGNIFLMLGSF
O. rehderiana GYPTSADLWQALGLA....YQRLGRFTAAVKSYGRAIELDDTDVFALVESGNIFLMLGSF
Q. robur GYPACPELWQALGLA....YQRLGRFTAALKSYGRAIELDNTNVFAMIESGNIFLMLGSF
F. sylvatica GYPACADLWQALGLA....YQRLGRYTAAI.............................
F. excelsior GFPTCADLWEALGLA....YQRMGMFTAALKSYGRAIELENSRVFALIESGNISLMLGSF
S. robusta RKGVEQFQQALKISPQSVSAQYGLASGLLGLSKECISLGAFKWGASLLE.....EACKVA
P. chinensis RKGVEQFQQALKISPQSVSARYGLASGLLGLSKECISLGAFKWGASQLE.....EACKVA
H. chinensis RKGVEQFQEALKLSPQSVSAQYGLASGLLGLSKEFISLGALKWGASLLE.....EACKVA
D. turbinatus RKGVEQFQQALKVSPQSVSAQYGLASGLLGLSRECISSGAFKWGASLLEASISIEACKVA
V. mangachapoi RKGVEQFQQVLKISPRSVPARYGLASGLLGLSKECISVGAFKWGASLLE.....EACKVA
B. pendula KKGVEQFLQALDISPQSVSAQYGLASGLLGLAKECINLGAFRWGASLLE.....EASSVA
O. rehderiana KKGVEQFLQALDISPQSLSAQYGLASGLLGLAKECINLGAFRWSASLLE.....EASSVA
Q. robur KEGVERFKQALEISHESVSARYGLASGLLGLAKAWINLGAFRWGASLLE.....EASEVA
F. sylvatica .KGVERFQQALKISPESVSAQYGLASGLLCLAKSCINLGAFKWGASLLE.....EASKVA
F. excelsior RKGVEQFQEALKTSPLNVSAHYGLGSAFLGFAKECINSGAFRWGATLLE.....EASKVA
S. robusta KASTRLSGNLSCIWKLLGDIQLTYAHFYPWAEESMSSKYDEETFNNSILSWRRCCLMAVI
P. chinensis KASTQLSGNLSCIWKLLGDIQLTYAQFYPWAEESMSSKYDEETFSNSILSWKRCCLMAVI
H. chinensis KASARLSGNFSCIWKLLGDIQLTYAQFYPWAEESMSSKYDDETFCNSILSWKRCCLMAAI
D. turbinatus KASTRLSGNLSCIWKLLGDIQLTYAQFYPWVEESMSSKYDEKTFSNSILSWQRCCLMAVI
V. mangachapoi KASTRLSGNLSCIWKLLGDIQLTYAQFYPWEEESMSSKYDEETFSNSILSWKRYCLMAAI
B. pendula KVGTHLAGNISCIWKLHGDIQLAYAKCFPWTEQGQGLEFDVEAFNASILSWKQTCCIAAV
O. rehderiana KAGTHLAGNISCIWKLHGDIQLAYAKCFPWTEQGQGLEFDAEAFNASILYWKQTCCIAAI
Q. robur KTSTHLAENMSCIWKLHGDIQLAYAKCFPWMEEGQGLEFDVETFNASILSWKKTCILAAI
F. sylvatica KASTHLAENMSCIWKLHGDVQLAYAKCFPWMEEGQGLEFDLDTFNASILSWKQTCSLAAI
F. excelsior ADGTQLSGNISCLWKLHGDIQLMYARCFPWMEEFRLSETDEKTFHYSINSWKSTCCLAAT

SKI3

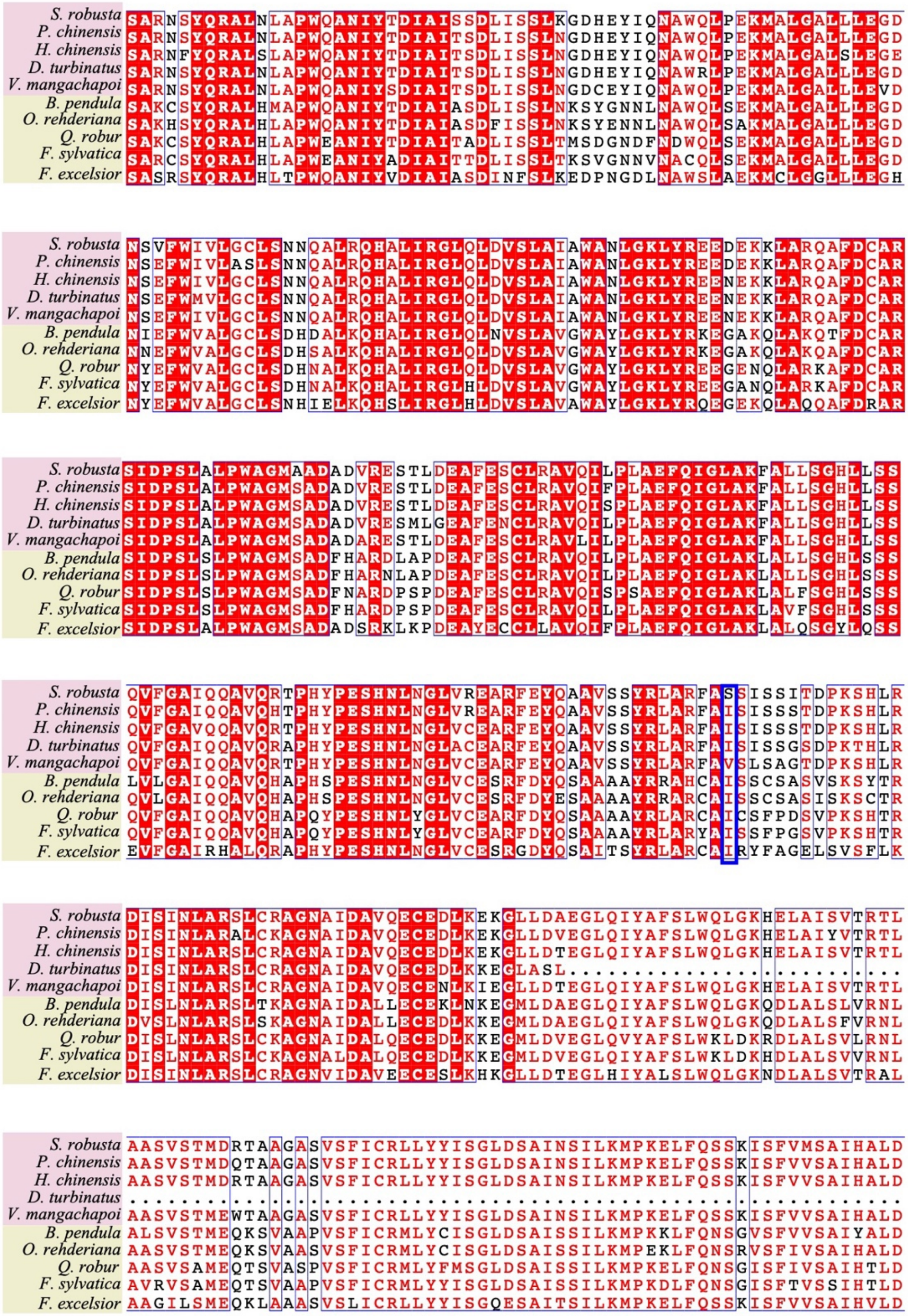

S. robusta
P. chinensis
H. chinensis
D. turbinatus
V. mangachapoi
B. pendula
O. rehderiana
Q. robur
F. sylvatica
F. excelsior

SKI3

```
S. robusta      QSNRLESVVSSSRYYLSSHEEISGMHYLIALNELIKHGLEDHLGFQSAVGHLRKALHMYP
P. chinensis    QSNRLESVVSSSCCYLSSHEEITGMHYLIALNNLIKHGLEDHLGFQSAVGHLRKALHKYP
H. chinensis    QSNRLESVVSSSRYFLSSHEEITGMHYLIALNKLIKHGLEEHLGFQSAIGHLRKALHMYP
D. turbinatus   ............................................................
V. mangachapoi  QSNRLESVVSSSRYFLSSHEEITGMHYLIALNKLIKNGLEDHLGFQSAVGHLRKALHMYP
B. pendula      QSNRLESVVSSSRSFVQSPVEVAGMHFMIALSKVVKHGTEFCLGVQSGVAHLRKALHMFP
O. rehderiana   QSNRLESVVSSSRSIVQSPVEVAGMHFLIALSKLVKQGTEFCLGVQCGIAHLRKALHMFP
Q. robur        TCDRLEPVVSSSRRFLTSHEEITEMHFLIALGKLVKHGTEFCLGFQSGVAHLRKALHMYP
F. sylvatica    ICNRLESVVSSSRCFLTSPEEIIGMHFLIALGKLVKHGTESCLGFQSGVAHLRKAIHMYP
F. excelsior    QNDQLESVVSSSRSSVTSDEEITAMHTLIAVGKLVKHGSKDCLAIQKGVDHLRKALHKYP

S. robusta      NSVLLRNLLGYLLLLSEDWNTTHVSSRCSIIDSSDY..KEGLKSAWEIIGAGAVACHVLC
P. chinensis    NSVLIR......................................................
H. chinensis    NSVLIRNLLGYLLLLSEDWNTTHVSSRCSIIDASDY..KEGLKSAWEIIGAGAVACYVLC
D. turbinatus   ............................................................
V. mangachapoi  NSVLIRNLLGYLLLLSEDWNTTHVSSRCSIIDASDH..KEGLKSAWEIIGAGAVACYVLG
B. pendula      NSSLMRNLLGYLLLSSKGWNDTHVATRCCNIDASDCAKKEGLKSASEILGAGAVACYAIG
O. rehderiana   NSSLMRNLLGYLLLSSKEWNDTHVATRCCNIDTSDCANKEGLKSASEILGAGAVACYAIG
Q. robur        NSSLIRNLLGYLLLSSKEWNDTHVATRCCNVDSTNYQNKGGLKSASEILGAGAVACYAVG
F. sylvatica    NSSLIRNLLGYLLLSSKEWNDTHVATRCCNV.STDRQNKEGLKSASEILGAGAVACYAVG
F. excelsior    NSSVLRNLLGYILLSSKEQRDLYLATRCSFIDLSDHKKEEGLKSASEIVGAGTVACYSTG

S. robusta      NREPKFSFPTCSYQCLNGPGSTKELQKCLRREPWNHNVRYLLVLNLLQKAREERFPVHLC
P. chinensis    ............................................................
H. chinensis    NREPKFSFPTCSYQCLNGSESIKELQKCFRREPWNHKPQYLLVLNLLQKAREERFPVHLC
D. turbinatus   ...........................CFRREPWNHNMQYLLVLNLLQKAREERFPVHLC
V. mangachapoi  NREPKFSFPTCSYRCLNGSGSIKELQKCFRREPWNHNAQYLLVLNLLQKAREERFPVHLC
B. pendula      NKNPKFSFPTCTYQCLKAPGAIQQLQKCLHQEPWNHNVRYLLILNFLQKAREERFPHHLC
O. rehderiana   NKNRKFSFPTCTYQCLKEPGAIQQLQKCLRQEPWNHNVRYLLILNFIQKAREERFPHNLC
Q. robur        NSNPKFSFPSCTYQCLNGHGAIQQLQKCLRREPWNHNIRYLLILNLLQKAREERFPHHLC
F. sylvatica    NSNPKLSFPTCTYQCLNEPGAIQQLQ..................................
F. excelsior    SYNGRFSFPTCRPQWPSGSGAIRLLQKNLHQEPWNLNMRYLLTVNYLQKAREERFPRHIC

S. robusta      TILERLIALSLSNEFYSGKDISCLYQKFQLLLCASEICLQGGNIAGCINLAKNASALFLP
P. chinensis    ............................................................
H. chinensis    TLLERLIALSLSNEFYSGKDISCRYQKFQLLLCASEICLQGGNIAGCINHAKNASALFLP
D. turbinatus   TILERLVALSLSNEFYSGKDTSCLYQKFQLLLCASEICLQVGNIADCINHAKNASALFLP
V. mangachapoi  TILEHLIALSLSNEFYSGKGISCLYQKFQLLLCASEICLQGGNIADCVNHAKNASALFLP
B. pendula      IVLERLISVALSSDLYTKTDMS..YQKFQLLLSASEIRLQSEDNIGCINHAKNASTLLLP
O. rehderiana   IVLKRLISVALSSDLYTKTDMS..YQKFQLLLCASEISLQSEDNIGCVNHAKNASTLLLP
Q. robur        IILKRLMSVALPSEVYSKTDNC..YQKFQLLLCASEISLHSGNKTGCIDHAKNASKLLLP
F. sylvatica    ............NEVYSKTDMC..YQKFQLLLCASEISLYSGNKIGCINHAKNASKLLLP
F. excelsior    LVLERLVAVALSNQSYSRKDLSSQYNMFHLLLCAAEVSLQQGNHSECLKHARLATGLSVH

S. robusta      ESYLFFGHLLLCRAYAAEGNCMNLQQEYKKCLELKTDFHVGWLCLKVIESQYNLQSGSNT
P. chinensis    ............................................................
H. chinensis    ESYLFFGHLLLCRAYAAEGNFMNVQQEYEKCLELKTDFHVGWLCLKVIESQYNLQTGSNT
D. turbinatus   ESYLFFGHLLLCRAYAAEGNFMNLQQEYENCLELKTDFHIGLLCLKVIESQYKLQTGSNT
V. mangachapoi  ESYLFFGHLVLCRAYAAEGNFMNLQQEYEKCLELKTDFHVGWLCLKVIECQYKLQTGSNT
B. pendula      DDYLFFSHLQLCRAYAAESDSLNIQKEYIRCLELKTDYHIGWICLKFIELRYNVQNDENI
O. rehderiana   DDYLFFSHLQLCRAYAAESDSLNLQKEYIRCLELKTDYRIGWICLKFIELRYNVQNDANI
Q. robur        DAYLFFSHLQLCRAYAVEGDIINLQKEYIRCLELKTDYHIGWICLKFVESQYHVQTDLNI
F. sylvatica    DAYLFFSHLQLCRAYAVEGDIINLQKEYLRCLELRTDYHIGWICLKLLESQYHVQTDLNI
F. excelsior    NSSLFFVHLLLCRAYAAENNIVSLTEEYRRCMELRTDSHIGWICLKFIESRYRLQNDSTI

S. robusta      IELSLNECSRGNSNSWNMWMAVVSLVLGLICIWNQDFLSAEEFLQHACSLAGADSCFFLC
P. chinensis    ............................................................
H. chinensis    IELSLSECSQGSSNSWNMWMAVVSLVLGLLCIWNQDFLSAEEFVQNACSLAGADSCFFLC
D. turbinatus   IELSLNECSRGSSNSWNMWMAVVSLVLGMICIWNQDFLSAEEFVQHACSLGGAESCFFLC
V. mangachapoi  IELSLNECSRGSSNSWNMWMAVVSLVLGLICTWNQDFLSAEEFVQHACSLAGADSCFFVC
B. pendula      LELSFKESSKERNYSWNMWMAVFNLVWGLISLWNEDFLSAEEFLTQACSLAGAESCLFLC
O. rehderiana   LELSSKESSKERNYSWNMGMAVFNLVWGLISLRNEDFLSAEEFLAQACSLAGAESCLLLC
Q. robur        LDLCFKECSTERDYSWNMWMAIFNLVFGLISIWNQDFLSAEEFLAQACSLAGAESCLFLC
F. sylvatica    LELSFKEYSKEENYSWNMWMARFYLVLGLISIWNQDFLSAEEFLAQACSSAGAESCLFLC
F. excelsior    LALNFEECSKDIKNSWNMWMAVFDLVQGLIAMWSGDFIGAEEFLMQACSAGGGESCFFLC
```

SKI3

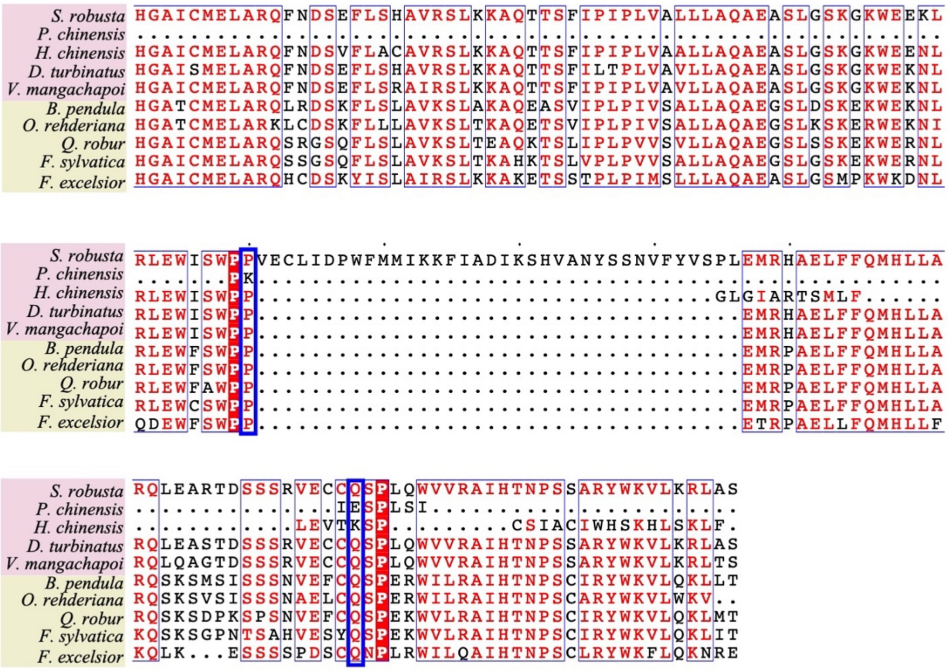
S. robusta HGAICMELARQFNDSEFLSHAVRSLKKAQTTSFIPIPLVALLLAQAEASLGSKGKWEEKL
P. chinensis ....
H. chinensis HGAICMELARQFNDSVFLACAVRSLKKAQTTSFIPIPLVAALLAQAEASLGSKGKWEENL
D. turbinatus HGAISMELARQFNDSEFLSHAVRSLKKAQTTSFILTPLVAVLLAQAEASLGSKGKWEKNL
V. mangachapoi HGAICMELARQFNDSEFLSRAIRSLKKAQTTSFIPIPLVAVLLAQAEASLGSKGKWEKNL
B. pendula HGATCMELARQLRDSKFLSLAVKSLAKAQEASVIPLPIVSALLAQAEGSLDSKEKWEKNL
O. rehderiana HGATCMELARKLCDSKFLLLAVKSLTKAQETSVIPLPIVSALLAQAEGSLKSKERWEKNI
Q. robur HGAICMELARQSRGSQFLSLAVKSLTEAQKTSLIPLPVVSVLLAQAEGSLSSKEKWERNL
F. sylvatica HGAICMELARQSSGSQFLSLAVKSLTKAHKTSLVPLPVVSALLAQAEGSLGSKEKWERNL
F. excelsior HGAICMELARQHCDSKYISLAIRSLKKAKETSSTPLPIMSLLLAQAEASLGSMPKWKDNL
S. robusta RLEWISWPPVECLIDPWFMMIKKFIADIKSHVANYSSNVFYVSPLEMRHAELFFQMHLLA
P. chinensis ....PK...
H. chinensis RLEWISWPP.................................GLGIARTSMLF.......
D. turbinatus RLEWISWPP....................................EMRHAELFFQMHLLA
V. mangachapoi RLEWISWPP....................................EMRHAELFFQMHLLA
B. pendula RLEWFSWPP....................................EMRPAELFFQMHLLA
O. rehderiana RLEWFSWPP....................................EMRPAELFFQMHLLA
Q. robur RLEWFAWPP....................................EMRPAELFFQMHLLA
F. sylvatica RLEWCSWPP....................................EMRPAELFFQMHLLA
F. excelsior QDEWFSWPP....................................ETRPAELLFQMHLLF
S. robusta RQLEARTDSSSRVECCQSPLQWVVRAIHTNPSSARYWKVLKRLAS
P. chinensis ....IESPLSI.......................
H. chinensis ............LEVTKSP........CSIACIWHSKHLSKLF.
D. turbinatus RQLEASTDSSSRVECCQSPLQWVVRAIHTNPSSARYWKVLKRLAS
V. mangachapoi RQLQAGTDSSSRVECCQSPLQWVVRAIHTNPSSARYWKVLKRLTS
B. pendula RQSKSMSISSSNVEFCQSPERWILRAIHTNPSCIRYWKVLQKLLT
O. rehderiana RQSKSVSISSSNAELCQSPERWILRAIHTNPSCARYWKVLWKV..
Q. robur RQSKSDPKSPSNVEFCQSPEKWVLRAIHTNPSCIRYWKVLQKLMT
F. sylvatica KQSKSGPNTSAHVESYQSPEKWVLRAIHTNPSCIRYWKVLQKLIT
F. excelsior KQLK...ESSSSPDSCQNPLRWILQAIHTNPSCLRYWKFLQKNRE

I RFK

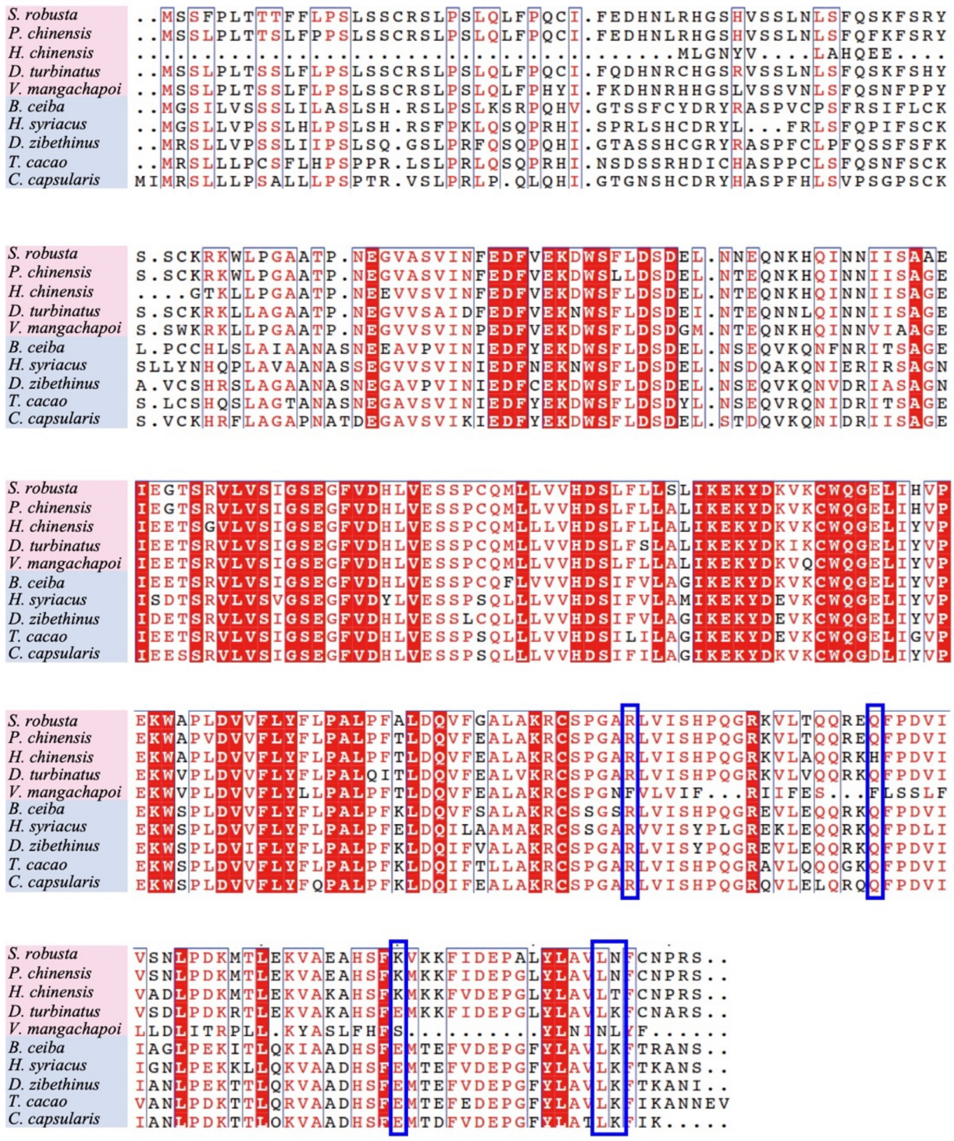

S. robusta
P. chinensis
H. chinensis
D. turbinatus
V. mangachapoi
B. ceiba
H. syriacus
D. zibethinus
T. cacao
C. capsularis

m APX

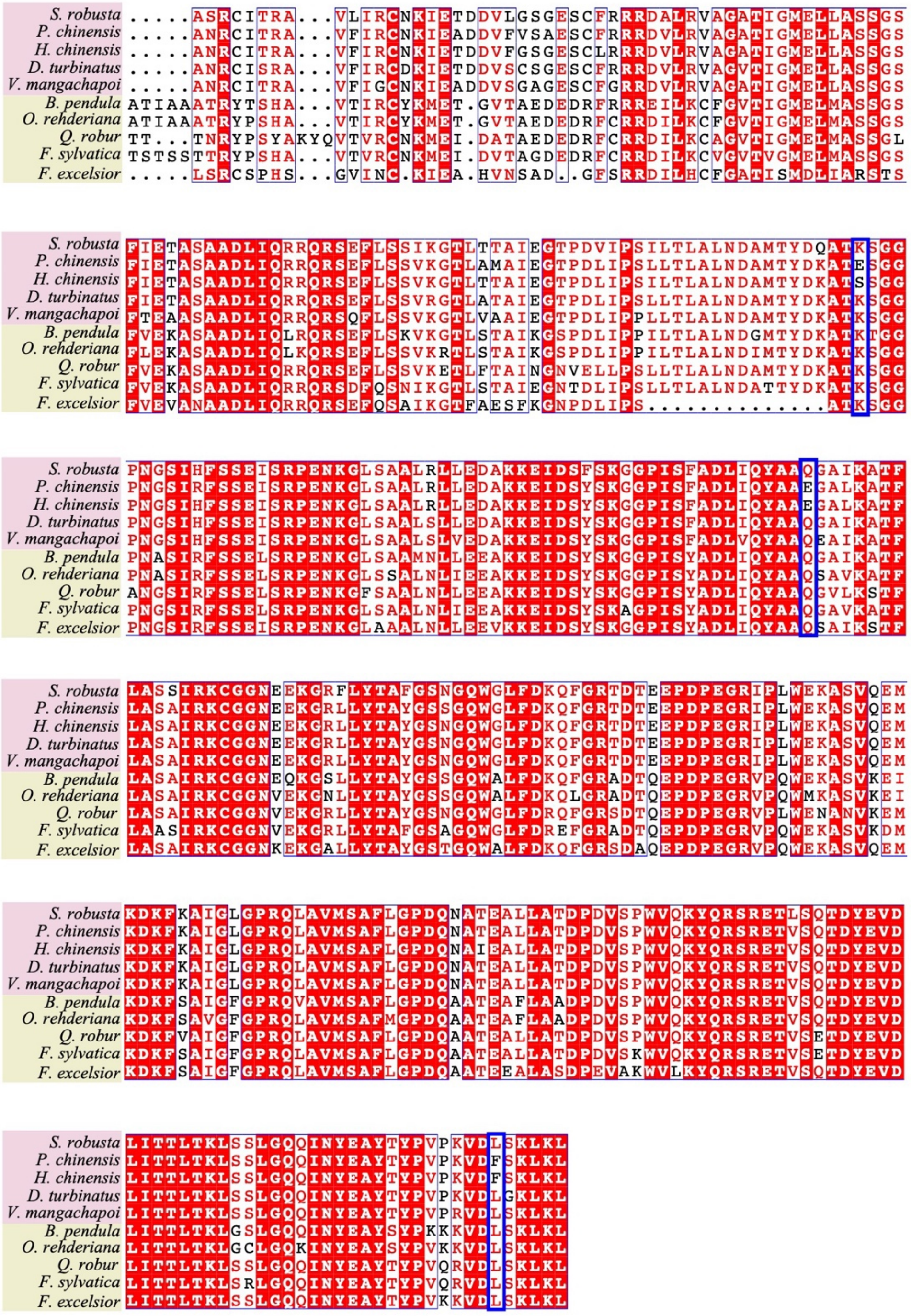
S. robusta
P. chinensis
H. chinensis
D. turbinatus
V. mangachapoi
B. pendula
O. rehderiana
Q. robur
F. sylvatica
F. excelsior

n XDH

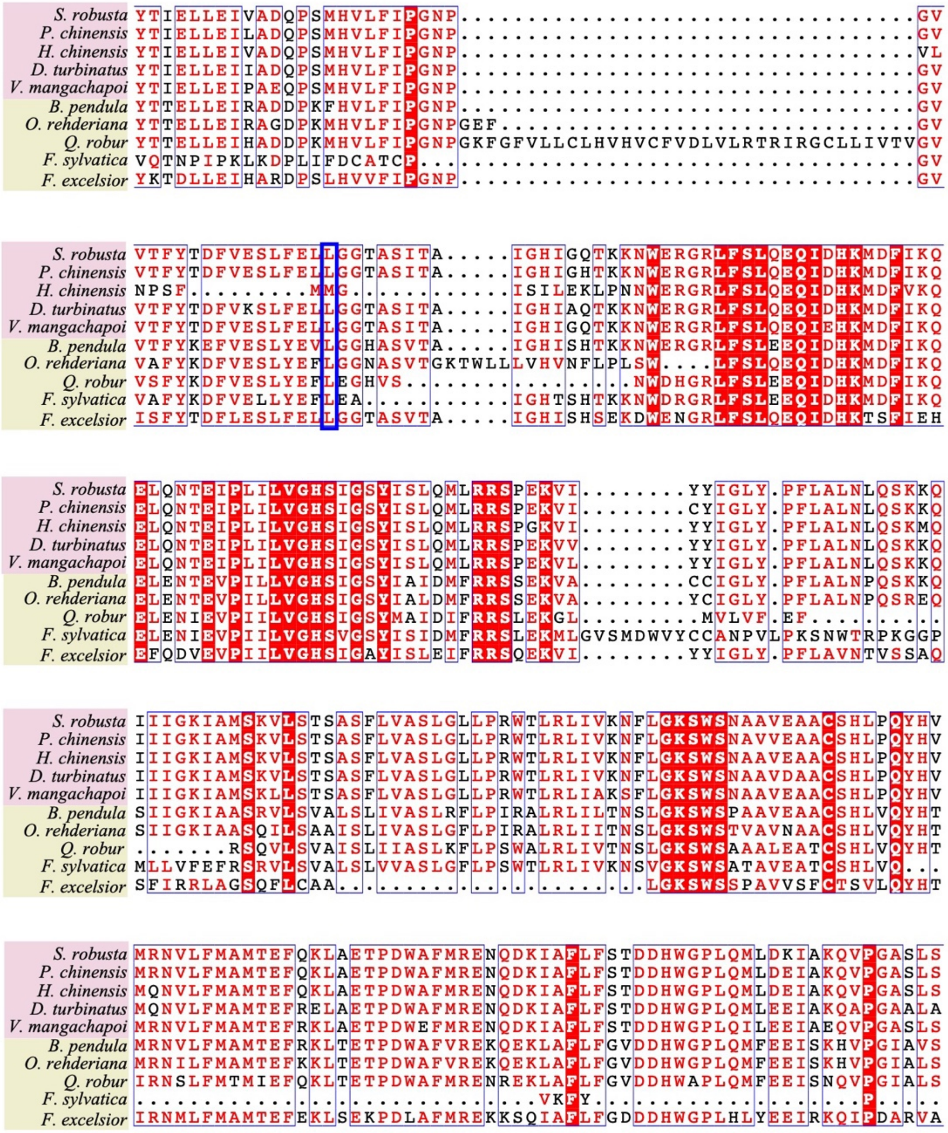

S. robusta
P. chinensis
H. chinensis
D. turbinatus
V. mangachapoi
B. pendula
O. rehderiana
Q. robur
F. sylvatica
F. excelsior

O GPS

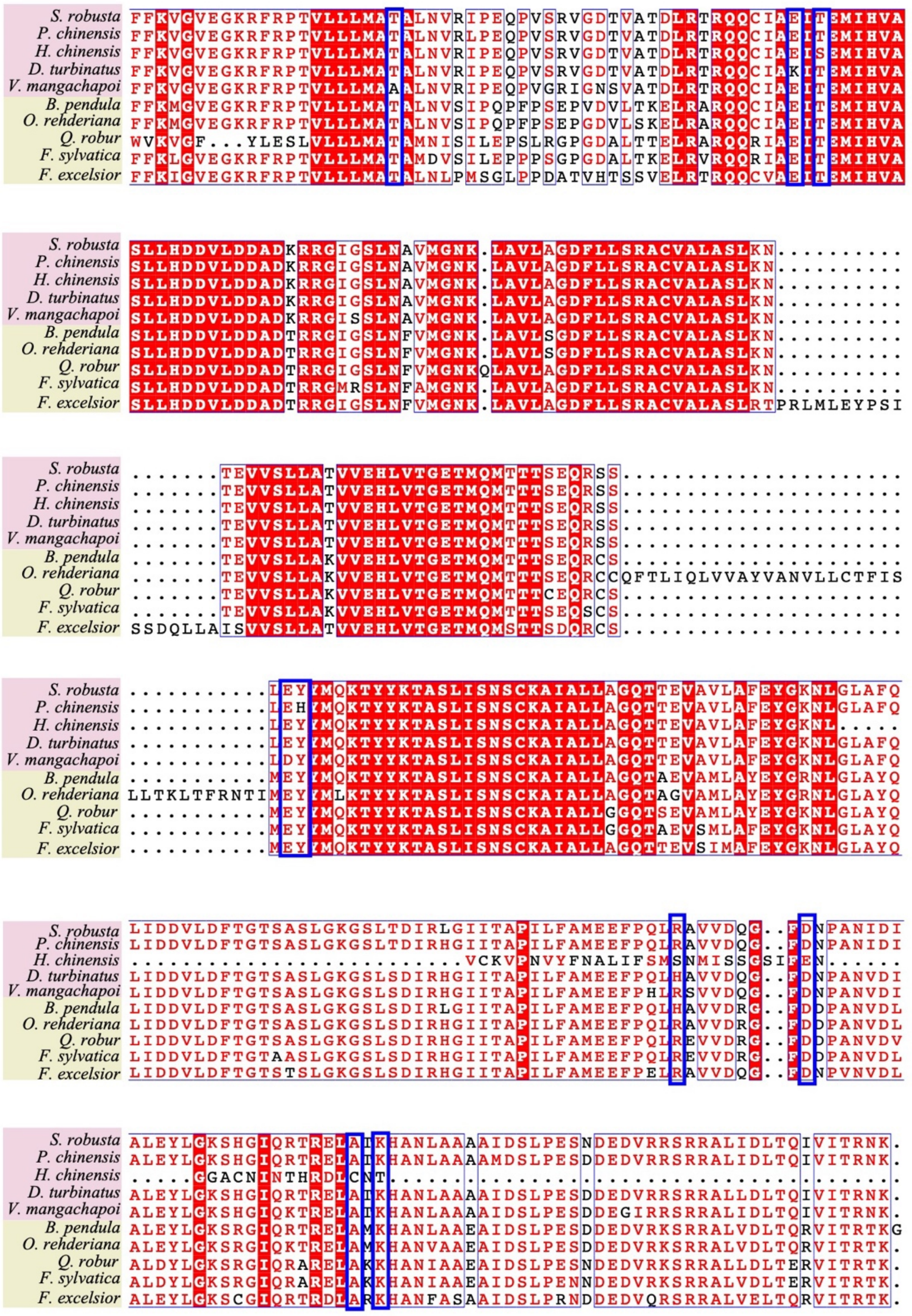
S. robusta
P. chinensis
H. chinensis
D. turbinatus
V. mangachapoi
B. pendula
O. rehderiana
Q. robur
F. sylvatica
F. excelsior

p ADH

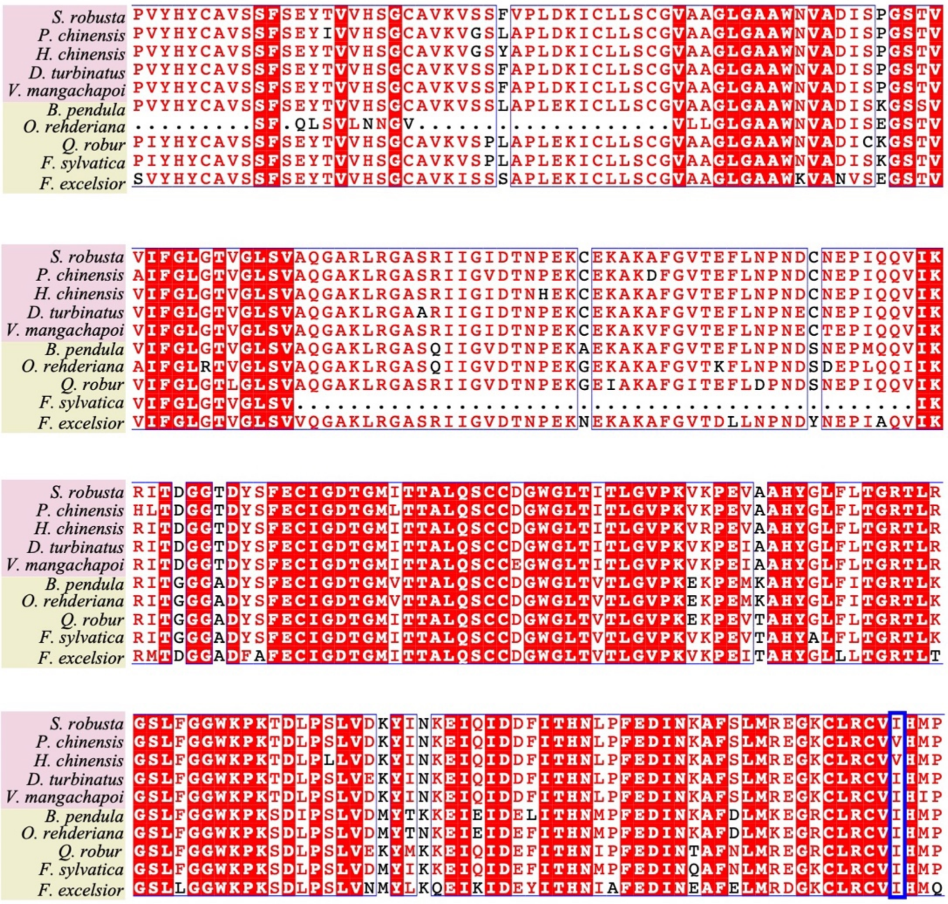
S. robusta PVYHYCAVSSFSEYTVVHSGCAVKVSSFVPLDKICLLSCGVAAGLGAAWNVADISPGSTV
P. chinensis PVYHYCAVSSFSEYIVVHSGCAVKVGSLAPLDKICLLSCGVAAGLGAAWNVADISPGSTV
H. chinensis PVYHYCAVSSFSEYTVVHSGCAVKVGSYAPLDKICLLSCGVAAGLGAAWNVADISPGSTV
D. turbinatus PVYHYCAVSSFSEYTVVHSGCAVKVSSFAPLDKICLLSCGVAAGLGAAWNVADISPGSTV
V. mangachapoi PVYHYCAVSSFSEYTVVHSGCAVKVSSFAPLDKICLLSCGVAAGLGAAWNVADISPGSTV
B. pendula PVYHYCAVSSFSEYTVVHSGCAVKVSSLAPLEKICLLSCGVAAGLGAAWNVADISKGSSV
O. rehderiana .........SF.QLSVLNNGV...................VLLGLGAAWNVADISEGSTV
Q. robur PIYHYCAVSSFSEYTVVHSGCAVKVSPLAPLEKICLLSCGVAAGLGAAWNVADICKGSTV
F. sylvatica PIYHYCAVSSFSEYTVVHSGCAVKVSPLAPLEKICLLSCGVAAGLGAAWNVADISKGSTV
F. excelsior SVYHYCAVSSFSEYTVVHSGCAVKISSSAPLEKICLLSCGVAAGLGAAWKVANVSEGSTV
S. robusta VIFGLGTVGLSVAQGARLRGASRIIGIDTNPEKCEKAKAFGVTEFLNPNDCNEPIQQVIK
P. chinensis AIFGLGTVGLSVAQGAKLRGASRIIGVDTNPEKCEKAKDFGVTEFLNPNDCNEPIQQVIK
H. chinensis VIFGLGTVGLSVAQGAKLRGASRIIGIDTNHEKCEKAKAFGVTEFLNPNDCNEPIQQVIK
D. turbinatus VIFGLGTVGLSVAQGAKLRGAARIIGIDTNPEKCEKAKAFGVTEFLNPNECNEPIQQVIK
V. mangachapoi VIFGLGTVGLSVAQGAKLRGASRIIGIDTNPEKCEKAKVFGVTEFLNPNECTEPIQQVIK
B. pendula VIFGLGTVGLSVAQGAKLRGASQIIGVDTNPEKAEKAKAFGVTEFLNPNDSNEPMQQVIK
O. rehderiana AIFGLRTVGLSVAQGAKLRGASQIIGVDTNPEKGEKAKAFGVTKFLNPNDSDEPLQQIIK
Q. robur VIFGLGTLGLSVAQGAKLRGASRIIGVDTNPEKGEIAKAFGITEFLDPNDSNEPIQQVIK
F. sylvatica VIFGLGTVGLSV..IK
F. excelsior VIFGLGTVGLSVVQGAKLRGASRIIGVDTNPEKNEKAKAFGVTDLLNPNDYNEPIAQVIK
S. robusta RITDGGTDYSFECIGDTGMITTALQSCCDGWGLTITLGVPKVKPEVAAHYGLFLTGRTLR
P. chinensis HLTDGGTDYSFECIGDTGMLTTALQSCCDGWGLTITLGVPKVKPEVAAHYGLFLTGRTLR
H. chinensis RITDGGTDYSFECIGDTGMITTALQSCCDGWGLTITLGVPKVRPEVAAHYGLFLTGRTLR
D. turbinatus RITDGGTDYSFECIGDTGMITTALQSCCDGWGLTITLGVPKVKPEIAAHYGLFLTGRTLR
V. mangachapoi RITDGGTDYSFECIGDTGMITTALQSCCEGWGLTITLGVPKVKPEIAAHYGLFLTGRTLR
B. pendula RITGGGADYSFECIGDTGMVTTALQSCCDGWGLTVTLGVPKEKPEMKAHYGLFITGRTLK
O. rehderiana RITGGGADYSFECIGDTGMVTTALQSCCDGWGLTVTLGVPKEKPEMKAHYGLFITGRTLK
Q. robur RITGGGADYSFECIGDTGMITTALQSCCDGWGLTVTLGVPKEKPEVTAHYGLFLTGRTLK
F. sylvatica RITGGGADYSFECIGDTGMITTALQSCCDGWGLTVTLGVPKVKPEVTAHYALFLTGRTLK
F. excelsior RMTDGGADFAFECIGDTGMITTALQSCCDGWGLTVTLGVPKVKPEITAHYGLLLTGRTLT
S. robusta GSLFGGWKPKTDLPSLVDKYINKEIQIDDFITHNLPFEDINKAFSLMREGKCLRCVIHMP
P. chinensis GSLFGGWKPKTDLPSLVDKYINKEIQIDDFITHNLPFEDINKAFSLMREGKCLRCVVHMP
H. chinensis GSLFGGWKPKTDLPLLVDKYINKEIQIDDFITHNLPFEDINKAFSLMREGKCLRCVVHMP
D. turbinatus GSLFGGWKPKTDLPSLVEKYINKEIQIDDFITHNLPFEDINKAFSLMREGKCLRCVIHMP
V. mangachapoi GSLFGGWKPKTDLPSLVDKYINKEIQIDDFITHNLPFEDINKAFSLMREGKCLRCVIHIP
B. pendula GSLFGGWKPKSDIPSLVDMYTKKEIEIDELITHNMPFEDINKAFDLMKEGRCLRCVIHMP
O. rehderiana GSLFGGWKPKSDLPSLVDMYTNKEIEIDEFITHNMPFEDINKAFDLMKEGRCLRCVIHMP
Q. robur GSLFGGWKPKSDLPSLVEKYMKKEIQIDEFITHNIPFEDINTAFNLMREGRCLRCVIHMP
F. sylvatica GSLFGGWKPKSDLPSLVDMYIKKEIQIDEFITHNMPFEDINQAFNLMREGKCLRCVIHMP
F. excelsior GSLLGGWKPKSDLPSLVNMYLKQEIKIDEYITHNIAFEDINEAFELMRDGKCLRCVIHMQ

q NGLY1

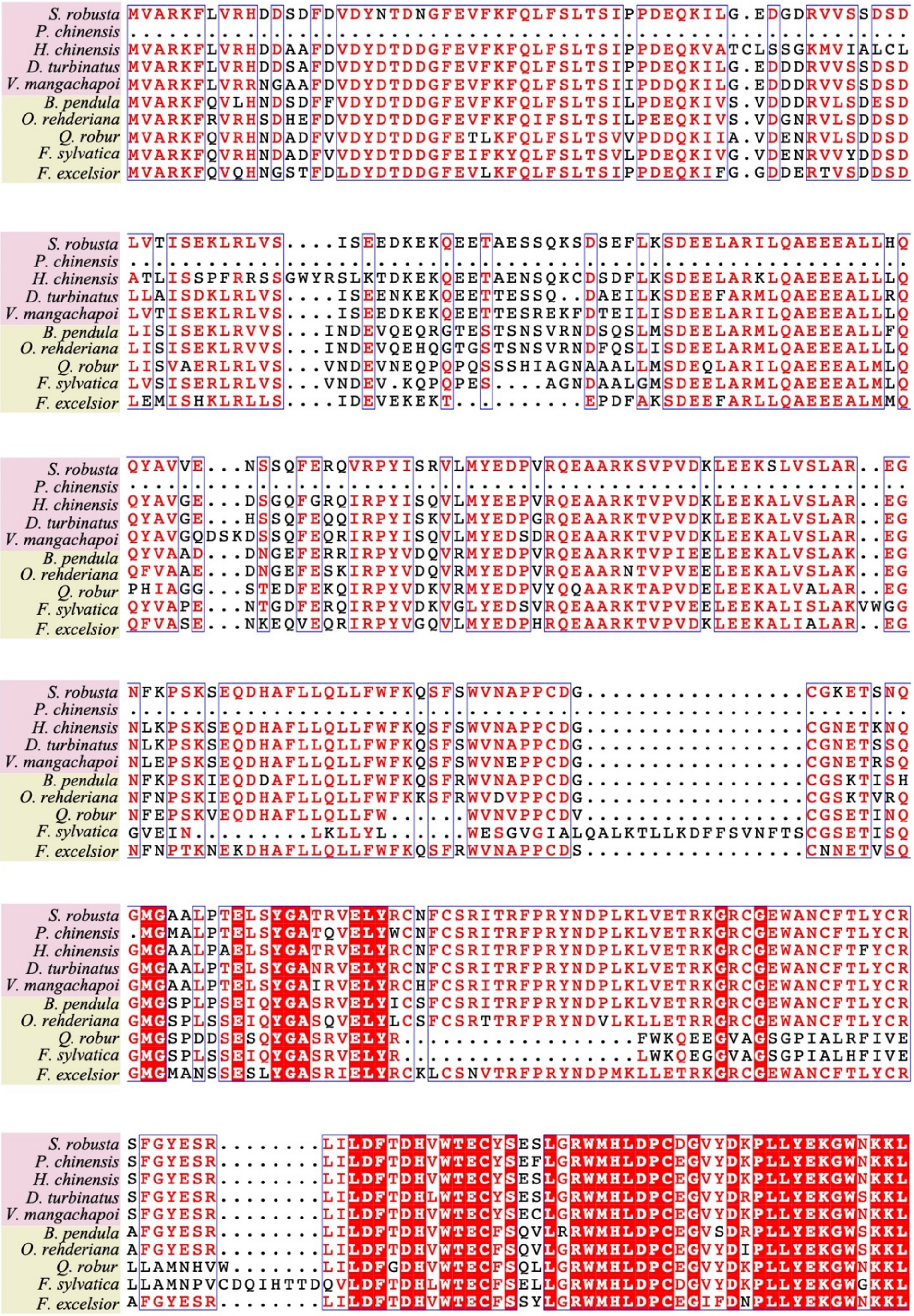
S. robusta
P. chinensis
H. chinensis
D. turbinatus
V. mangachapoi
B. pendula
O. rehderiana
Q. robur
F. sylvatica
F. excelsior

NGLY1

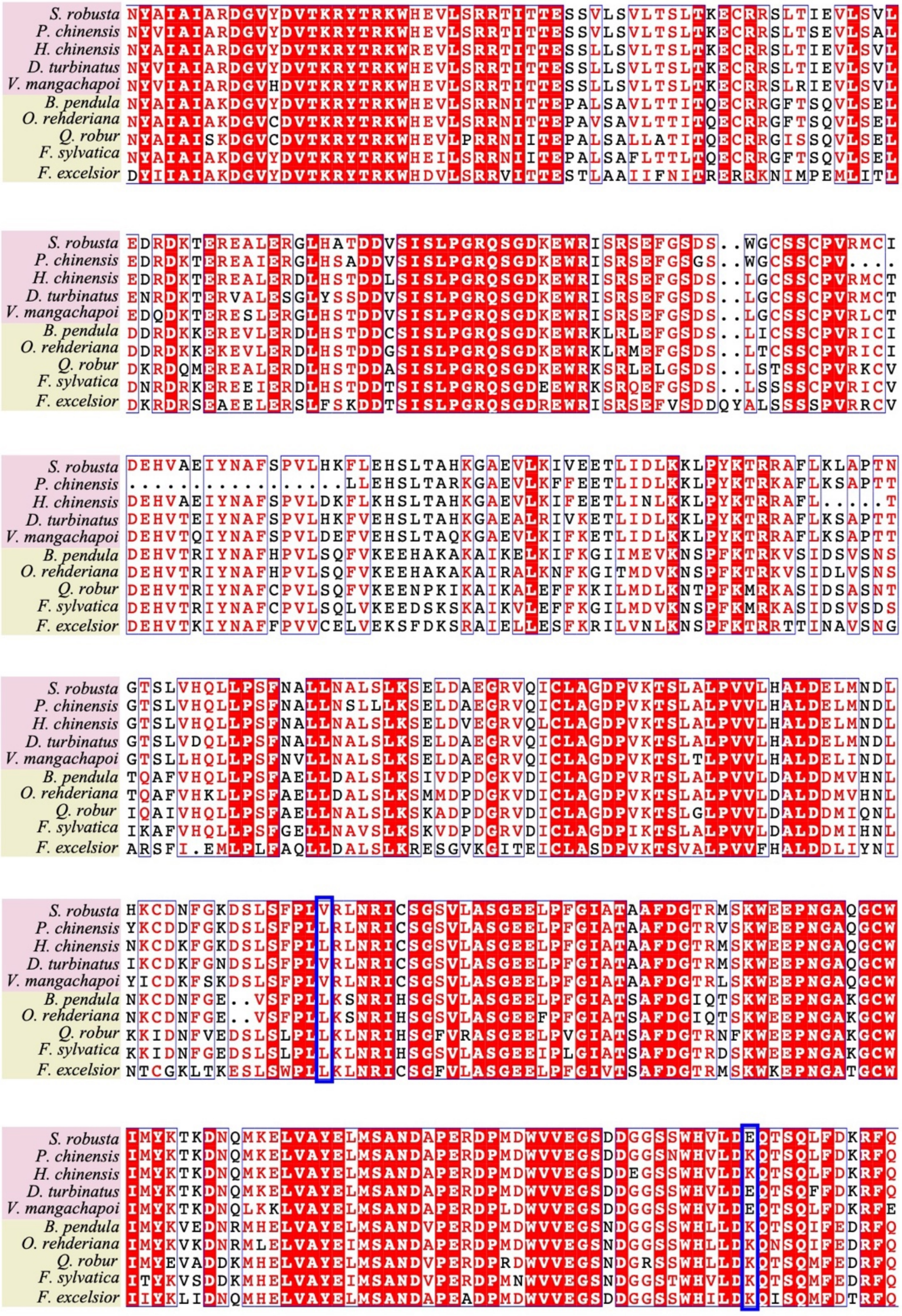
S. robusta
P. chinensis
H. chinensis
D. turbinatus
V. mangachapoi
B. pendula
O. rehderiana
Q. robur
F. sylvatica
F. excelsior

r NDH

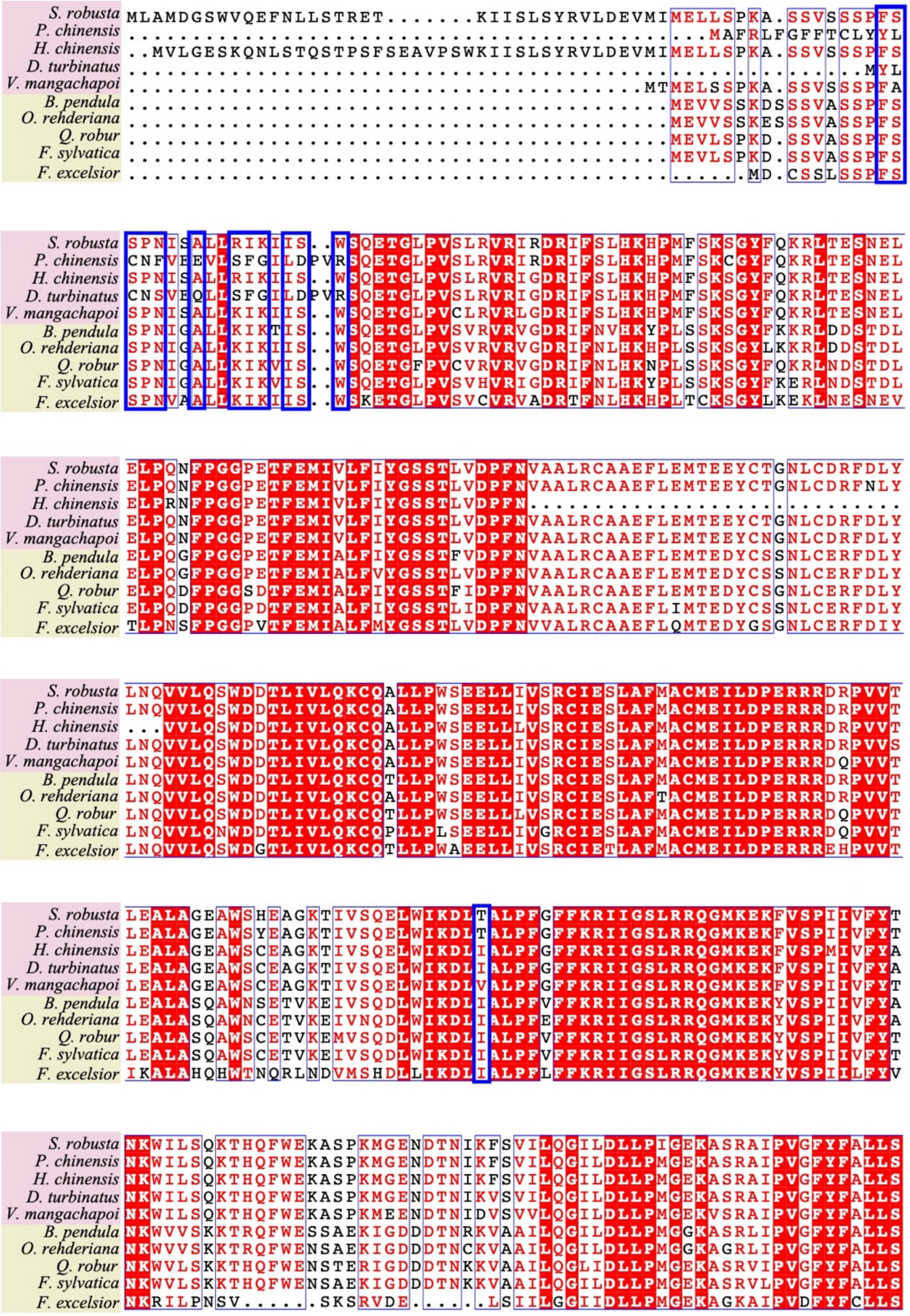
S. robusta
P. chinensis
H. chinensis
D. turbinatus
V. mangachapoi
B. pendula
O. rehderiana
Q. robur
F. sylvatica
F. excelsior

NDH

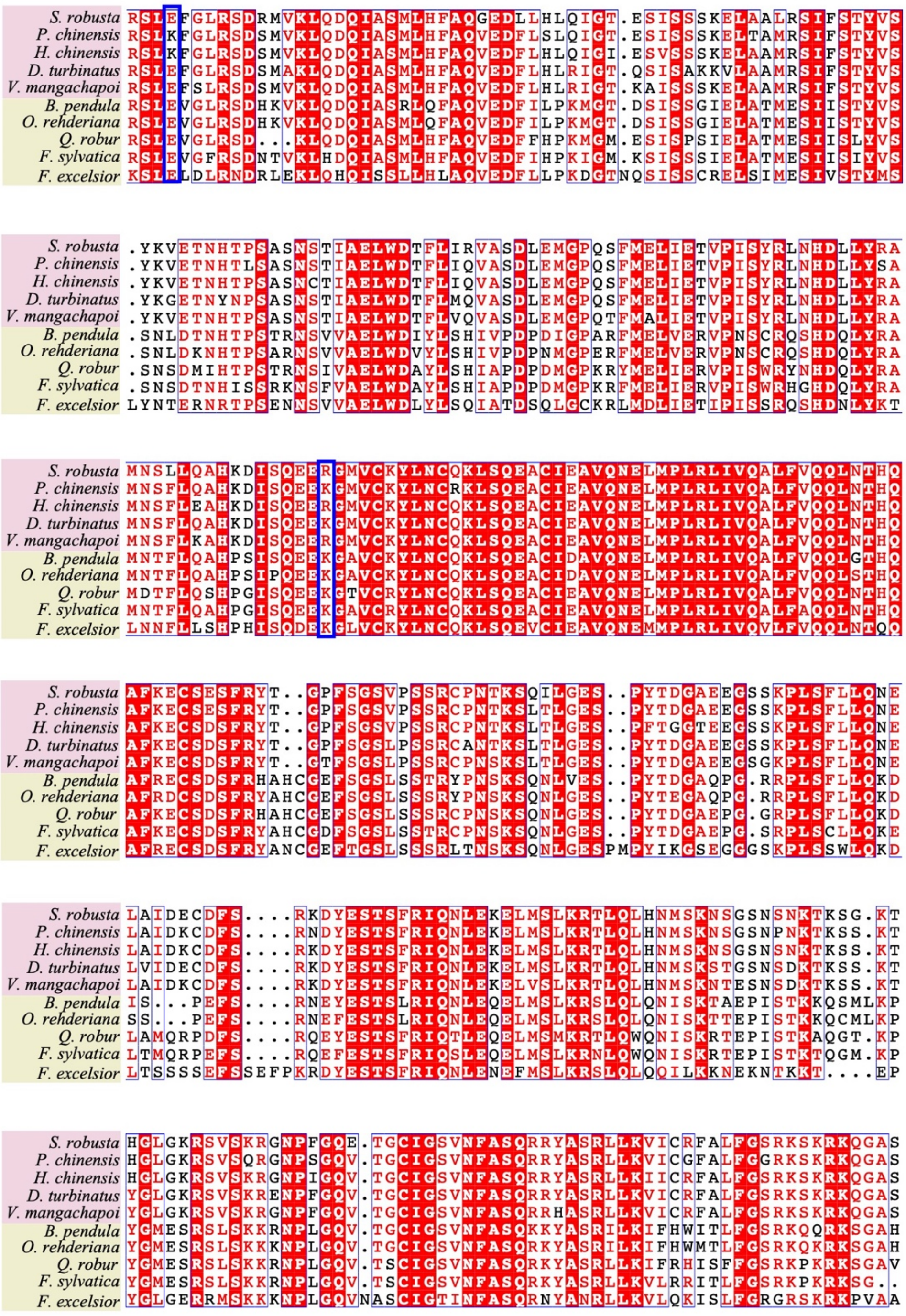

S. robusta
P. chinensis
H. chinensis
D. turbinatus
V. mangachapoi
B. pendula
O. rehderiana
Q. robur
F. sylvatica
F. excelsior

S GRPE

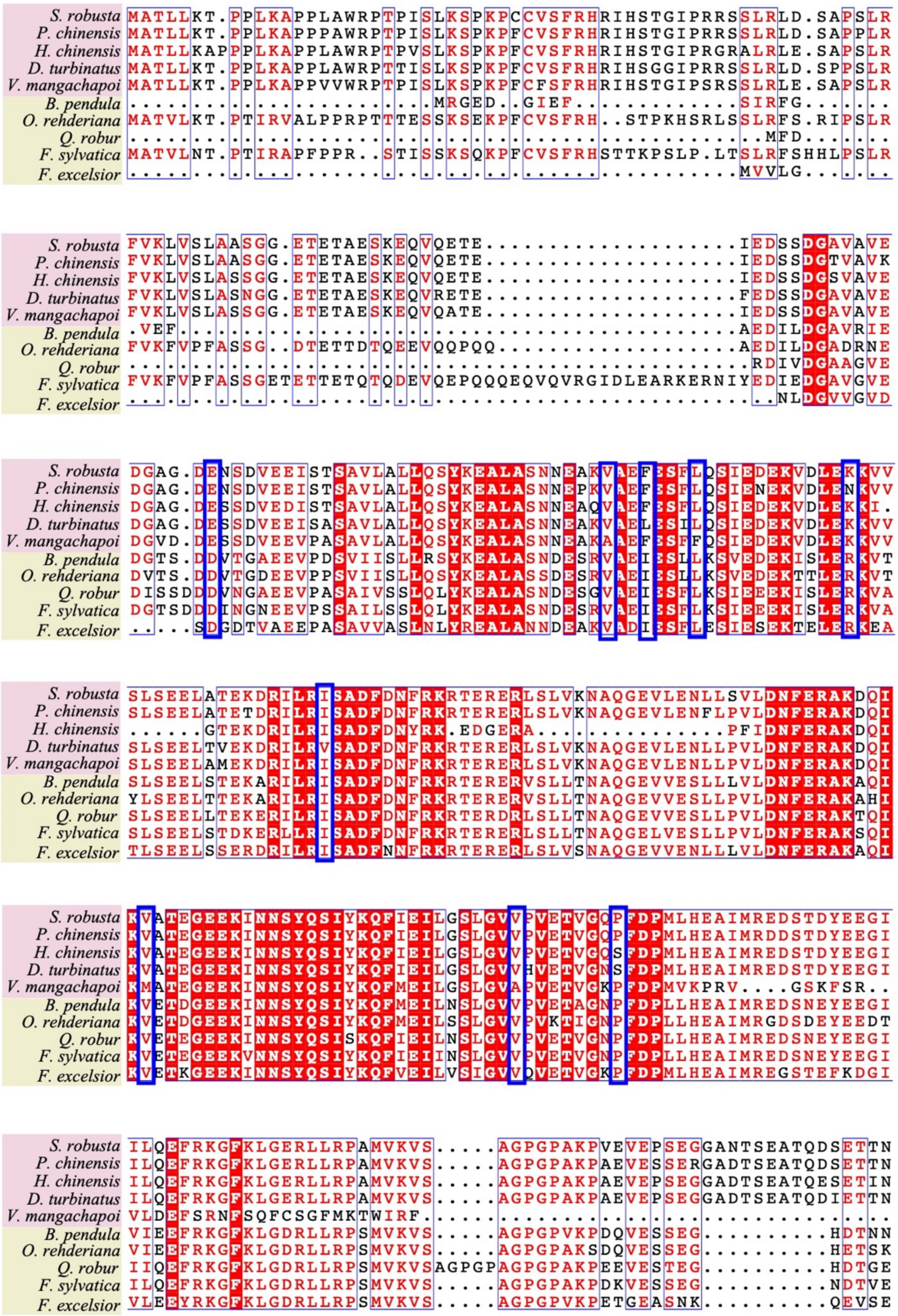
S. robusta
P. chinensis
H. chinensis
D. turbinatus
V. mangachapoi
B. pendula
O. rehderiana
Q. robur
F. sylvatica
F. excelsior

t USP

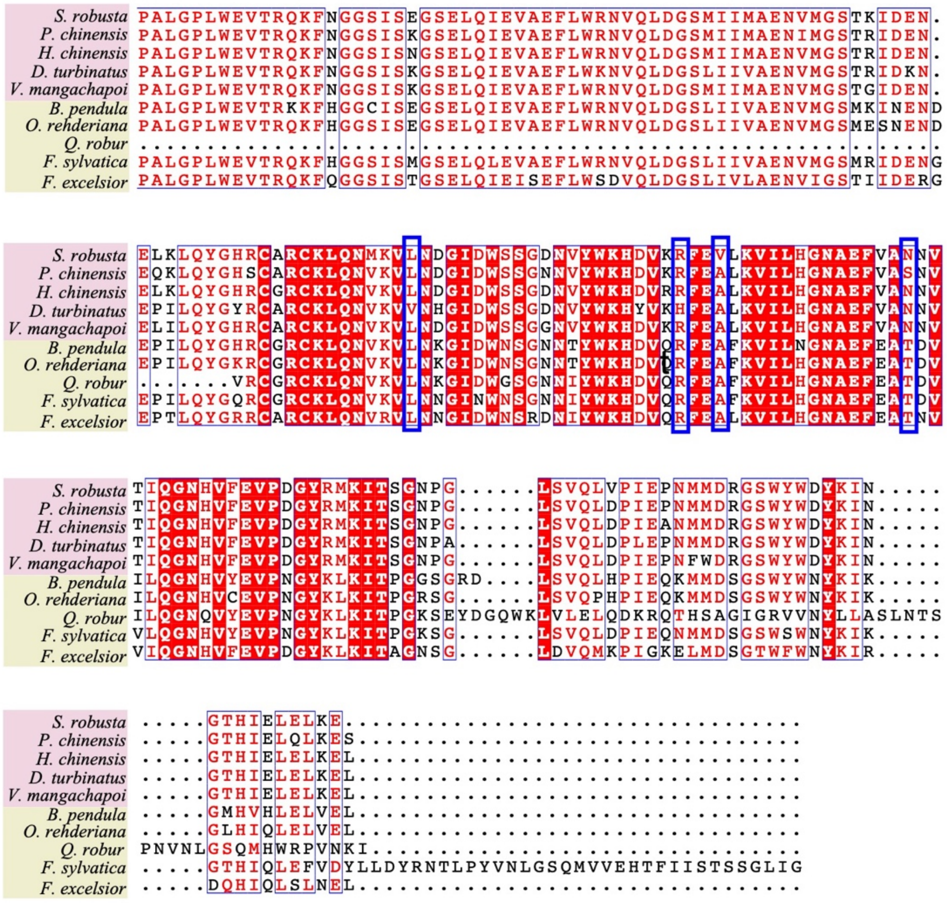

Supplementary Figure 7. Positively selected sites in RAD51D (a), SMC5 (b), LUT1 (c), DNA2 (d), cysQ (e), BLM (f), LRPPPRC (g), LSD1 (h), SMG1 (i), PPP2R5 (j), SKI3 (k), PFK (l), APX (m), XDH1 (n), GPS (o), ADH (p), NGLY1 (q), NDH (r), GRPE (s) and USP (t). Blue frames are used to highlight the positively selected sites supported by the comparison between the genomes of our focal Dipterocarpoideae species and those of five temperate tree species. The function of each gene is shown in Supplementary Data 2.

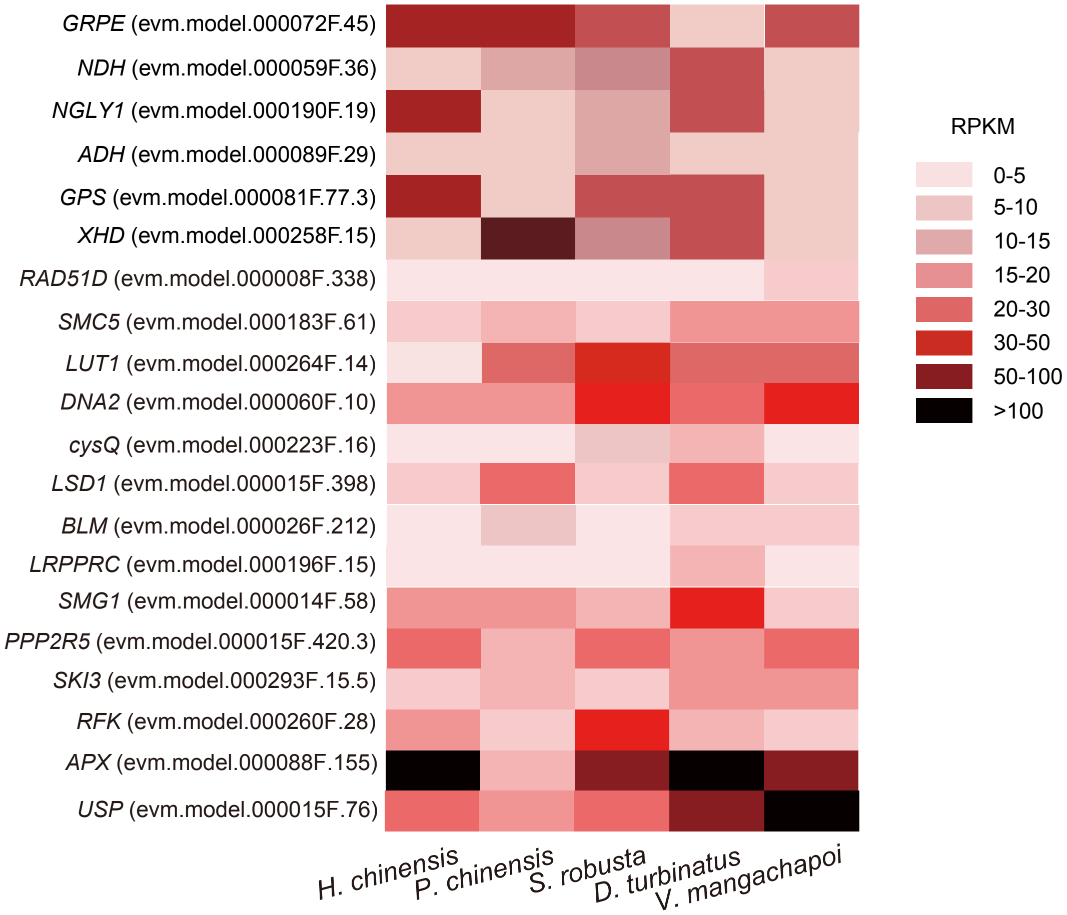

Supplementary Figure 8. Expression of the 20 positively selected genes involved in the responses of plants to environmental stresses in the comparisons between the genomes of our focal Dipterocarpoideae species and those of five temperate tree species comparisons of genomes. The function of each gene is shown in Supplementary Data 2. Expression level of each gene is estimated based on the transcriptome data (Illumina RNA-seq) for gene structure prediction (see Supplementary Table 21). RPKM: reads per kilobase per million mapped reads. Source data are provided as a Source Data file.

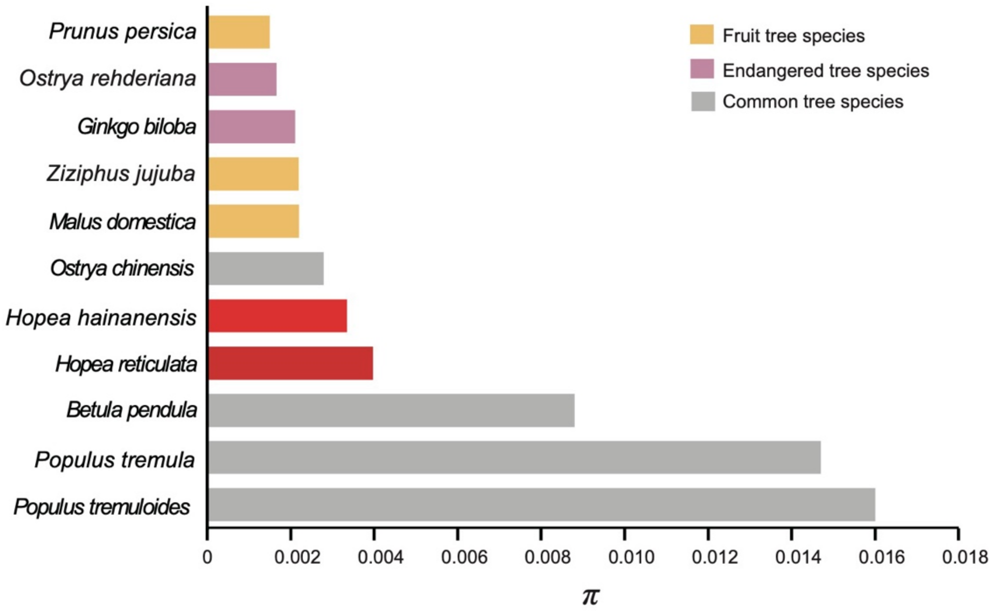

Supplementary Figure 9. Nucleotide diversity (π) of *Hopea hainanensis* and *H. reticulata* populations at the genomic level, with further comparisons with π values from the genomes of other nine tree species[6,7,8,9,10,11,12]. Source data are provided as a Source Data file.

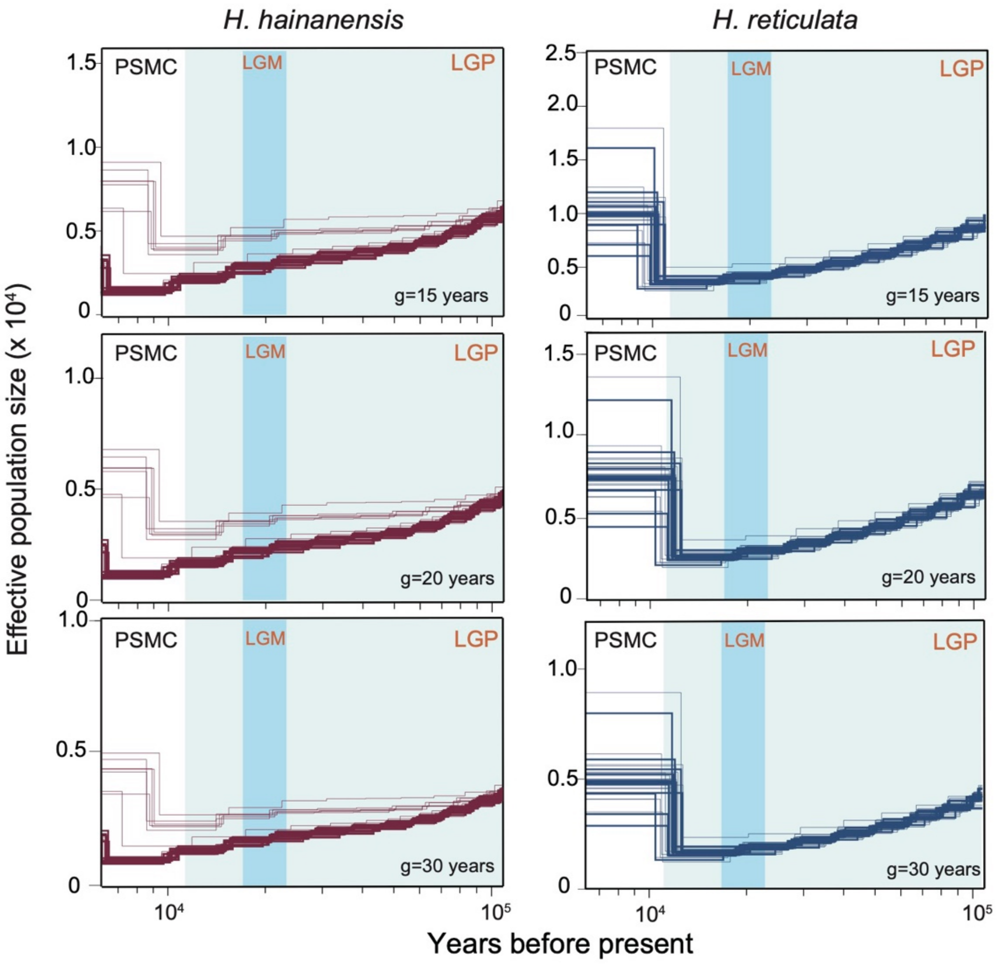

Supplementary Figure 10. Demographic dynamics of *H. hainanensis* (n=30) and *H. reticulata* (n=32) inferred using PSMC.

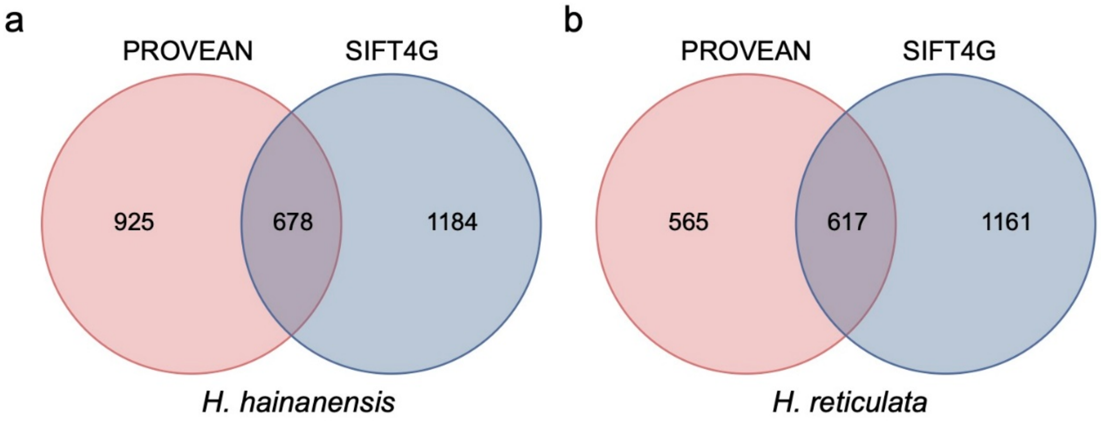

Supplementary Figure 11. Venn diagrams of the deleterious mutations (DDMs) detected within *H. hainanensis* (n=30) and *H. reticulata* (n=32) populations based on the results from analyses using PROVEAN and SIFT4G.

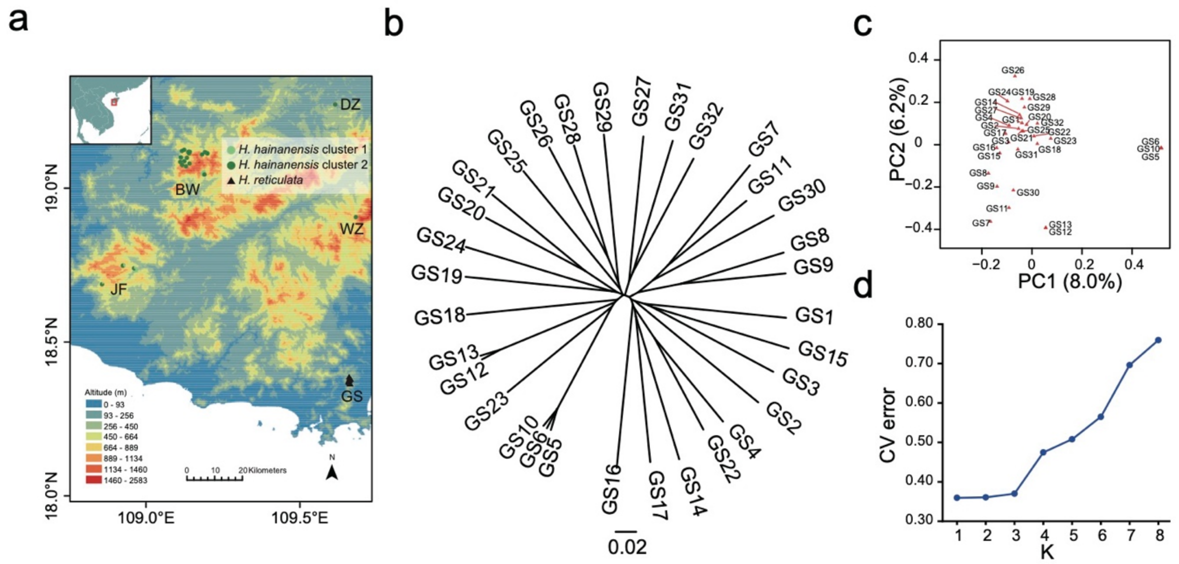

Supplementary Figure 12. The sampling locations of wild *H. hainanensis* and *H. reticulata* trees for resequencing and analysis of population genomics (a), neighbour-joining phylogenetic tree (b), principal component analysis (PCA) (c) and results of genetic structure analysis for the *H. reticulata* (n=32) population using genetic clustering (d). The sampling map was drawn by Chao-Nan Liu. All sampled trees are located on Hainan Island, China. BW: Bawangling, DZ: Danzhou city, JF: Jianfengling, WZ: Wuzhi mountain, and GS: Ganshiling. Cross-validation error (CV error) of each assumed genetic group number (K) was estimated, and assignment of sampled trees were performed according to the first two principal components from PCA analysis. Source data of Supplementary Fig. 12d are provided as a Source Data file.

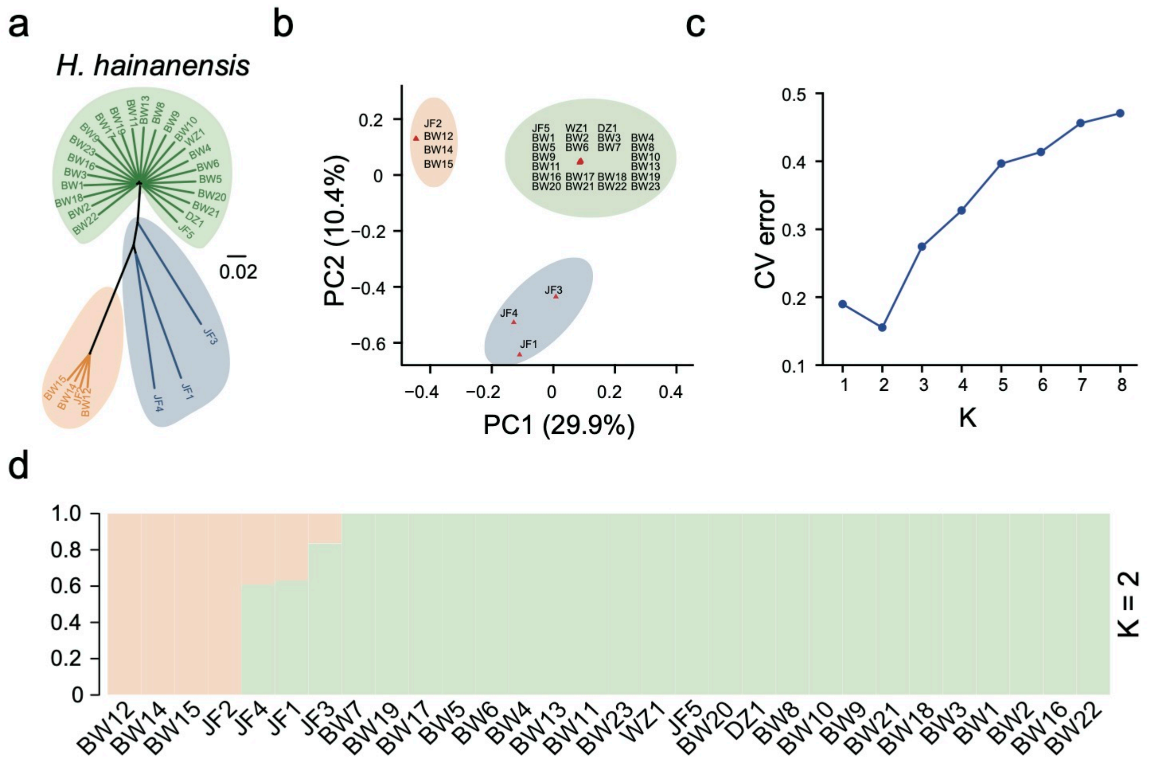

Supplementary Figure 13. Results of neighbour-joining phylogenetic tree (a), principal component analysis (PCA) (b), and genetic clustering (c, d) for the sampled trees of *H. hainanensis* (n=30). Cross-validation error (CV error) for each assumed genetic group number (*K*) was estimated, and assignment of sampled trees were performed according to the first two principal components from PCA analysis. Source data of Supplementary Fig. 13c are provided as a Source Data file.

Supplementary Tables

Supplementary Table 1. The results of genome size estimation using flow cytometry setting the genome of *Solanum lycopersicum* (genome size: 2.07 Gb; 2C = 2.12) as the reference.

Sample	Fuorescence intensity	CV error (%)	2C (pg)	Genome size (Gb)	Ploidy
Hopea chinensis	18.20	4.30	0.81	0.79	2X
Solanum lycopersicum	47.40	1.76	2.12	2.07	
Hopea hainanensis	29.20	4.71	1.60	1.56	4X
Solanum lycopersicum	38.80	3.61	2.12	2.07	
Hopea reticulata	26.90	4.88	1.64	1.60	4X
Solanum lycopersicum	34.70	3.29	2.12	2.07	

Supplementary Table 2. Statistics of clean sequencing data. Pacbio CLR (continuous long-read) reads were generated for *H. hainanensis*, *H. reticulata*, *H. chinensis* and *P. chinensis*, and Pacbio HiFi (high fidelity) reads were generated for *S. robusta*, *D. turbinatus* and *V. mangachapoi*. Depth of sequencing was calculated via clean sequencing data divided by the genome size estimated by *k*-mer analysis (see Table 1).

Species	Abbreviation	Illumina reads		Pacbio reads		Hi-C reads	
		Data (Gb)	Depth (X)	Data (Gb)	Depth (X)	Data (Gb)	Depth (X)
H. hainanensis	Hhai	263.53	168.53	97.17	62.14	77.32	49.45
H. reticulata	Hret	77.13	51.40	87.34	58.21	100.29	66.84
H. chinensis	Hchi	51.16	147.66	42.63	123.04	45.19	130.43
P. chinensis	Pchi	79.65	254.36	124.88	398.80	47.11	136.00
S. robusta	Srob	80.59	244.98	6.64	20.18	50.29	152.87
D. turbinatus	Dtur	82.38	220.76	20.72	55.52	57.42	153.88
V. mangachapoi	Vman	69.98	154.55	8.38	18.51	47.56	105.01

Supplementary Table 3. Proportions of highly homologous regions in each chromosome of *H. hainanensis* and *H. reticulata* according to the coverage depth of Illumina pair-end reads for *k*-mer analysis. The overall sequencing depth was 144X (223.69 Gb/1.56 Gb) for *H. hainanensis* and 51X (77.13 Gb/1.50 Gb) for *H. reticulata*. Regions with coverage depths of 216X – 360X, 360X– 504X and larger than 504X for *H. hainanensis* and of 75.5X – 127.5X, 127.5X – 178.5X and larger than 178.5X for *H. reticulata* (i.e., 1.5 – 2.5, 2.5 – 3.5 and larger than 3.5 folds of overall coverage depth) were thought to be the overlaps of two, three and four sets chromosomes, respectively. The total length of overlapped region was estimated to be 361.86 Mb for *H. hainanensis* and 245.66 Mb for *H. reticulata*, accounting for most (77.9% for *H. hainanensis* and 93.6% for *H. reticulata*) of the difference between the assembled and the estimated genome size (see Methods).

Chromosome_ID	*H. hainanensis* Chromosome length (bp)	216X–360X	360X–504X	>504X	*H. reticulata* Chromosome length (bp)	76.5X–127.5X	127.5X–178.5X	>178.5X
Chr1	56,060,860	0.2368	0.0389	0.0296	66,518,347	0.2481	0.0207	0.0054
Chr2	50,862,877	0.2322	0.0083	0.0022	62,617,095	0.2431	0.0179	0.0040
Chr3	47,502,918	0.1823	0.0187	0.0120	52,787,304	0.2071	0.0125	0.0030
Chr4	25,558,352	0.0630	0.0055	0.0000	29,144,642	0.3376	0.0477	0.0079
Chr5	52,060,348	0.2239	0.0035	0.0012	69,171,435	0.1840	0.0250	0.0039
Chr6	51,918,603	0.2779	0.0270	0.0252	61,919,302	0.1649	0.0241	0.0058
Chr7	45,882,048	0.2183	0.0179	0.0111	44,322,556	0.1633	0.0244	0.0020
Chr8	29,356,050	0.0651	0.0020	0.0003	26,589,551	0.1835	0.0267	0.0034
Chr9	53,961,569	0.3009	0.0445	0.0752	61,289,770	0.2457	0.0238	0.0073
Chr10	52,201,287	0.2773	0.0063	0.0019	54,261,871	0.2230	0.0140	0.0024
Chr11	38,126,576	0.1828	0.0121	0.0100	46,031,668	0.1853	0.0130	0.0059
Chr12	24,842,783	0.0535	0.0008	0.0000	25,649,789	0.1930	0.0304	0.0062
Chr13	44,572,804	0.3358	0.0121	0.0011	54,107,632	0.2173	0.0181	0.0031
Chr14	44,276,399	0.3243	0.0486	0.0788	46,457,576	0.2561	0.0198	0.0045
Chr15	36,124,573	0.2729	0.0246	0.0075	44,011,096	0.2493	0.0195	0.0052
Chr16	22,986,945	0.0622	0.0004	0.0000	41,307,522	0.2041	0.0136	0.0019
Chr17	51,368,458	0.3222	0.0444	0.0779	49,638,002	0.2877	0.0234	0.0068

Chr18	43,707,180	0.3276	0.0078	0.0014	48,129,990	0.3171	0.0322	0.0087
Chr19	28,019,024	0.2591	0.0300	0.0232	44,406,130	0.3137	0.0277	0.0068
Chr20	22,359,377	0.0894	0.0009	0.0009	31,106,232	0.2845	0.0267	0.0045
Chr21	42,884,734	0.2733	0.0581	0.0212	50,897,133	0.1819	0.0173	0.0067
Chr22	41,336,008	0.2288	0.0068	0.0010	46,730,095	0.2382	0.0201	0.0077
Chr23	33,386,394	0.1836	0.0210	0.0099	43,902,429	0.2237	0.0141	0.0023
Chr24	22,647,172	0.0645	0.0013	0.0000	16,233,104	0.1694	0.0074	0.0031
Chr25	30,718,104	0.3955	0.0557	0.1045	34,780,442	0.3108	0.0210	0.0052
Chr26	27,906,433	0.3973	0.0097	0.0018	31,998,611	0.3194	0.0441	0.0181
Chr27	17,551,768	0.2813	0.0177	0.0188	25,311,145	0.2947	0.0245	0.0008
Chr28	14,580,805	0.1385	0.0014	0.0007	20,671,113	0.3952	0.0556	0.0227
Total	1,052,760,449	-	-	-	1,299,991,582	-	-	-

Supplementary Table 4. Lengths of assembled chromosomes for each focal species. Note that only monoploid genome was assembled for all species except the two autotetraploid species *H. hainanensis* and *H. reticulata*.

Chromosome ID	Chromosome length (bp)						
	H. hainanensis	*H. reticulata*	*H. chinensis*	*P. chinensis*	*S. robusta*	*D. turbinatus*	*V. mangachapoi*
Chr1	56,060,860	66,518,347	58,999,814	47,060,898	52,458,056	40,721,971	35,234,553
Chr2	50,862,877	62,617,095	50,750,874	43,239,849	50,588,708	26,050,962	56,851,045
Chr3	47,502,918	52,787,304	53,466,247	51,893,975	54,130,500	35,475,413	39,502,375
Chr4	25,558,352	29,144,642	51,441,790	46,059,081	48,473,062	32,353,911	37,717,841
Chr5	52,060,348	69,171,435	44,472,741	39,227,045	42,867,569	45,090,292	40,985,457
Chr6	51,918,603	61,919,302	46,293,359	29,970,300	46,457,883	29,150,171	49,753,518
Chr7	45,882,048	44,322,556	32,845,991	51,556,864	31,767,639	26,610,888	43,556,076
Chr8	29,356,050	26,589,551	-	-	-	29,516,284	36,579,020
Chr9	53,961,569	61,289,770	-	-	-	42,617,143	33,201,765
Chr10	52,201,287	54,261,871	-	-	-	28,594,060	29,296,269
Chr11	38,126,576	46,031,668	-	-	-	32,794,201	36,846,320
Chr12	24,842,783	25,649,789	-	-	-	-	-
Chr13	44,572,804	54,107,632	-	-	-	-	-
Chr14	44,276,399	46,457,576	-	-	-	-	-
Chr15	36,124,573	44,011,096	-	-	-	-	-
Chr16	22,986,945	41,307,522	-	-	-	-	-
Chr17	51,368,458	49,638,002	-	-	-	-	-
Chr18	43,707,180	48,129,990	-	-	-	-	-
Chr19	28,019,024	44,406,130	-	-	-	-	-
Chr20	22,359,377	31,106,232	-	-	-	-	-
Chr21	42,884,734	50,897,133	-	-	-	-	-
Chr22	41,336,008	46,730,095	-	-	-	-	-
Chr23	33,386,394	43,902,429	-	-	-	-	-

Chr24	22,647,172	16,233,104	-	-	-	-	-
Chr25	30,718,104	34,780,442	-	-	-	-	-
Chr26	27,906,433	31,998,611	-	-	-	-	-
Chr27	17,551,768	25,311,145	-	-	-	-	-
Chr28	14,580,805	20,671,113	-	-	-	-	-
Total (bp)	1,052,760,449	1,229,991,582	338,270,816	309,008,012	326,743,417	368,975,296	439,524,239

Supplementary Table 5. Genome completeness assessments using CEGMA, BUSCO and Illumina sequencing data for *k*-mer analysis (see Supplementary Table 2). A total of 248 core eukaryotic genes (CEGs) and 1,614 conserved embryophyta proteins (CEPs) were used in CEGMA and BUSCO assessments, respectively. LMG: the longest monoploid genome.

	**CEGMA**			**BUSCO**			**Illumina sequencing data**	
Species	Type	Number of CEGs	Percentage (%)	Type	Number of CEPs	Percentage (%)	Mapping ratio (%)	Genome coverage (%)
H. hainanensis (four sets chromosomes)	Fully mapped CEGs	244	96.77	Complete	1,598	99.0	97.38	98.42
	Fully + partially mapped CEGs	245	98.79	Fragmented	6	0.4		
				Missing	10	0.6		
H. hainanensis (LMG)	Fully mapped CEGs	226	91.13	Complete	1,398	86.6	86.21	99.81
	Fully + partially mapped CEGs	232	93.55	Fragmented	26	1.6		
				Missing	190	11.8		
H. reticulata (four sets chromosomes)	Fully mapped CEGs	236	95.16	Complete	1,580	97.9	96.08	92.38
	Fully + partially mapped CEGs	242	97.58	Fragmented	3	0.2		
				Missing	31	1.9		
H. reticulata (LMG)	Fully mapped CEGs	223	89.92	Complete	1,319	81.7	84.21	97.12
	Fully + partially mapped CEGs	227	91.53	Fragmented	34	2.1		
				Missing	261	16.2		
H. chinensis	Fully mapped CEGs	236	95.16	Complete	1,533	95.0	94.45	99.91
	Fully + partially mapped CEGs	240	98.39	Fragmented	10	0.6		
				Missing	71	4.4		
P. chinensis	Fully mapped CEGs	236	95.16	Complete	1,583	98.1	95.41	99.84
	Fully + partially mapped CEGs	239	96.37	Fragmented	7	0.4		
				Missing	24	1.5		
S. robusta	Fully mapped CEGs	236	95.16	Complete	1,590	98.5	96.44	99.99
	Fully + partially mapped CEGs	239	96.37	Fragmented	7	0.4		
				Missing	17	1.1		

D. turbinatus	Fully mapped CEGs	245	98.79	Complete	1,598	99.0	98.69	99.85
	Fully + partially mapped CEGs	248	100.00	Fragmented	3	0.2		
				Missing	13	0.8		
V. mangachapoi	Fully mapped CEGs	229	92.34	Complete	1,567	97.1	96.86	99.90
	Fully + partially mapped CEGs	234	94.35	Fragmented	3	0.2		
				Missing	44	2.7		

Supplementary Table 6. Allele annotation among homologous chromosomes in *H. hainanensis* and *H. reticulata* genomes. Number of allele-identified genes was calculated as four times of the total number of genes with four alleles across all the seven chromosome sets, three times of the total number of genes with three alleles, two times of the total number of genes with two alleles, and the total number of genes with one allele. Chromosome set: a group of clustered chromosomes containing four homologous chromosomes.

	H. hainanensis				***H. reticulata***			
	Genes with four alleles	Genes with three alleles	Genes with two alleles	Genes with one allele	Genes with four alleles	Genes with three alleles	Genes with two alleles	Genes with one allele
Chromosome set 1	612	1,681	1,762	3,007	345	1,630	2,211	4,341
Chromosome set 2	503	1,712	1,663	2,613	248	1,418	1,954	3,903
Chromosome set 3	498	1,985	1,939	2,806	371	1,356	2,005	3,865
Chromosome set 4	391	1,649	1,809	2,158	336	1,386	1,855	3,485
Chromosome set 5	365	1,690	1,822	2,370	165	1,178	2,200	3,079
Chromosome set 6	440	1,517	1,478	2,231	193	1,203	1,719	3,467
Chromosome set 7	169	964	1,207	1,467	245	749	1,216	1,918
Allele-identified genes	2,978	11,198	11,680	16,652	1,903	8,920	13,160	24,058

Supplementary Table 7. Annotation results of protein-coding genes and BUSCO assessments. A total of 1,614 conserved embryophyta proteins (CEPs) were used in BUSCO assessments. CDS: coding sequence.

		H. hainanensis	*H. reticulata*	*H. chinensis*	*P. chinensis*	*S. robusta*	*D. turbinatus*	*V. mangachapoi*
Gene structure	Gene number	88,703	85,031	29,372	29,651	29,104	31,744	31,006
	Average gene length (bp)	2,690	2,781	2,773	2,872	2,974	2,824	2,910
	Average CDS length (bp)	1,127	1,171	1,132	1,163	1,184	1,181	1,163
	Average exons per gene	5.1	5.3	5.2	5.3	5.4	5.2	5.2
	Average exon length (bp)	221	220	217	219	220	229	226
	Average intron length (bp)	382	373	389	397	409	395	421
BUSCO assessment	Complete CEPs	1,512 (93.7%)	1,513 (93.7%)	1,515 (93.9%)	1,483 (91.9%)	1,500 (92.9%)	1,513 (93.7%)	1,473 (91.3%)
	Single-copy CEPs	175 (10.9%)	162 (10.0%)	1,306 (80.9%)	1,293 (80.1%)	1,330 (82.4%)	1,286 (79.7%)	1,306 (80.9%)
	Duplicated CEPs	1,337 (82.8%)	1,351 (83.7%)	209 (13.0%)	190 (11.8%)	170 (10.5%)	227 (14.0%)	167 (10.4%)
	Fragmented CEPs	39 (2.4%)	34 (2.1%)	45 (2.8%)	54 (3.3%)	47 (2.9%)	34 (2.1%)	75 (4.6%)
	Missing CEPs	63 (3.9%)	67 (4.2%)	54 (3.3%)	77 (4.8%)	67 (4.2%)	67 (4.2%)	66 (4.1%)
Functional annotation	Swiss-Prot	70,086 (79.0%)	69,960 (82.3%)	23,213 (79.0%)	23,148 (78.1%)	22,747 (78.2%)	24,173 (76.2%)	23,833 (76.9%)
	NR	85,925 (97.9%)	84,422 (99.3%)	28,391 (96.7%)	28,427 (95.9%)	27,821 (95.6%)	30,641 (96.5%)	29,436 (94.9%)
	KEGG	65,391 (73.7%)	65,126 (76.6%)	21,789 (74.2%)	21,638 (73.0%)	21,295 (73.2%)	22,092 (69.6%)	22,475 (72.5%)
	GO	80,762 (91.0%)	77,970 (91.7%)	26,742 (91.1%)	27,022 (91.1%)	26,564 (91.3%)	28,531 (89.9%)	28,206 (91.0%)
	Pfam	68,287 (77.0%)	68,799 (80.9%)	22,664 (77.2%)	22,728 (76.7%)	22,455 (77.2%)	23,370 (73.6%)	23,451 (75.6%)
	Overall	88,341 (99.6%)	84,932 (99.9%)	29,234 (99.5%)	29,498 (99.5%)	28,950 (99.5%)	31,604 (99.6%)	30,807 (99.4%)

Supplementary Table 8. Annotation results of repeat sequences (using Tandem Repeats Finder (TRF), RepeatMasker and RepeatProteinMask) and non-coding RNAs. LTR-RT: long terminal repeat retrotransposon.

	**Repeat sequences**			**Non-coding RNA**				
Species	Type	Size (bp)	% of genome	Type	Number	Average length (bp)	Total length (bp)	% of genome
H. hainanensis	TRF	43,630,344	3.97	miRNA	786	120.48	94,696	0.0086
	RepeatMasker	524,298,168	47.70	tRNA	1,857	74.94	139,162	0.0127
	RepeatProteinMask	81,936,177	7.45	rRNA	300	196.98	59,093	0.0054
	**Total**	**542,963,148**	**49.39**	snRNA	1,526	107.82	164,529	0.0150
	LTR-RT	345,420,890	31.42					
H. reticulata	TRF	50,220,382	4.06	miRNA	1,149	115.33	132,512	0.0107
	RepeatMasker	560,712,793	45.29	tRNA	2,213	75.10	166,187	0.0134
	RepeatProteinMask	107,112,585	8.65	rRNA	550	236.08	129,845	0.0105
	**Total**	**587,772,479**	**47.48**	snRNA	1,772	110.15	195,182	0.0158
	LTR-RT	401,299,478	32.42					
H. chinensis	TRF	12,880,263	3.79	miRNA	381	121.51	46,296	0.0136
	RepeatMasker	143,083,785	42.15	tRNA	655	74.99	49,118	0.0145
	RepeatProteinMask	30,960,981	9.12	rRNA	336	240.52	80,814	0.0238
	**Total**	**150,108,579**	**44.22**	snRNA	528	108.33	57,198	0.0169
	LTR-RT	90,401,075	26.63					
P. chinensis	TRF	10,767,710	3.43	miRNA	338	115.04	38,882	0.0124
	RepeatMasker	132,085,261	42.08	tRNA	623	75.25	46,880	0.0149
	RepeatProteinMask	20,963,852	6.68	rRNA	125	380.54	47,567	0.0152
	**Total**	**136,971,552**	**43.64**	snRNA	471	109.80	51,715	0.0165
	LTR-RT	62,301,462	19.85					
S. robusta	TRF	11,405,760	3.49	miRNA	419	119.87	50,227	0.0153
	RepeatMasker	143,206,917	43.76	tRNA	631	75.36	47,551	0.0145
	RepeatProteinMask	25,266,318	7.72	rRNA	136	187.62	25,517	0.0078

	**Total**	**148,596,107**	**45.40**	snRNA	427	107.40	45,858	0.0140
	LTR-RT	79,262,279	24.22					
D. turbinatus	TRF	15,795,432	4.08	miRNA	1,052	141.58	148,939	0.0385
	RepeatMasker	188,535,626	48.69	tRNA	2,187	75.52	165,168	0.0427
	RepeatProteinMask	27,975,591	7.22	rRNA	7,057	400.77	2,828,206	0.7300
	**Total**	**195,643,900**	**50.53**	snRNA	660	112.92	74,529	0.0192
	LTR-RT	108,354,887	27.98					
V. mangachapoi	TRF	23,606,357	5.27	miRNA	445	114.46	50,936	0.0114
	RepeatMasker	216,261,711	48.29	tRNA	583	75.10	43,783	0.0098
	RepeatProteinMask	57,223,275	12.78	rRNA	3,447	175.89	606,284	0.1400
	**Total**	**230,664,898**	**51.51**	snRNA	460	115.99	53,357	0.0119
	LTR-RT	178,277,869	39.81					

Supplementary Table 9. Results of gene family clustering by comparing genomes of our focal species with those of additional 12 Dipterocarpoideae species and other six species, five temperate tree species.

Species	Gene number	Genes in families	Family number	Unique families	Reference
Dipterocarpus alatus	29,203	37,119	24,002	215	13
Dipterocarpus gracilis	27,310	34,063	23,009	106	13
Dipterocarpus intricatus	26,797	33,843	22,753	86	13
Dipterocarpus turbinatus	42,423	29,587	18,183	45	This study
Dipterocarpus zeylanicus	38,375	33,869	23,062	93	13
Gossypium raimondii	37,824	30,040	14,976	371	14
Hopea chinensis	31,744	26,501	17,976	23	This study
Hopea reticulata	37,356	41,351	18,619	432	This study
Hopea hainanensis	33,724	35,651	19,643	342	This study
Hopea mollissima	29,296	31,750	21,327	129	13
Hopea odorata	48,040	36,665	21,647	417	13
Parashorea chinensis	42,508	26,348	17,757	60	This study
Shorea henryana	35,939	29,946	20,042	89	13
Shorea leprosula	44,078	30,259	19,965	188	5
Shorea robusta	35,359	26,475	18,046	51	This study
Shorea roxburghii	29,650	30,441	20,048	99	13
Theobroma cacao	33,950	20,170	14,531	119	15
Vatica mangachapoi	34,469	27,774	18,593	54	This study
Vatica odorata	29,104	32,945	21,821	179	13
Vatica rassak	34,473	32,266	21,637	276	13
Vatica xishuangbannaensis	21,109	31,889	21,692	103	13
Oryza sativa	31,006	23,717	12,779	1,818	16
Arabidopsis thaliana	39,825	23,561	13,131	801	17
Aquilaria sinensis	39,603	22,261	13,899	799	18
Amborella trichopoda	36,861	19,088	12,084	1,034	19
**Clustered with 5 temperate tree species**					
Betula pendula	24,629	19,530	13,776	395	9
Dipterocarpus turbinatus	31,744	27,801	16,529	143	This study
Fraxinus excelsior	38,948	31,146	14,180	974	20
Fagus sylvatica	61,548	55,954	16,355	2,168	21
Hopea chinensis	29,296	25,584	17,351	54	This study
Hopea exalata	48,040	40,630	18,170	611	This study
Hopea hainanensis	42,508	34,501	18,765	484	This study
Ostrya rehderiana	27,767	22,146	15,060	700	6
Parashorea chinensis	29,650	25,634	17,191	87	This study
Quercus robur	25,808	21,616	12,218	585	22
Shorea robusta	29,104	25,378	17,148	83	This study
Vatica mangachapoi	31,006	25,745	16,651	205	This study

Supplementary Table 10. Results of quota tests by comparing the genomes of our focal species with that of *Vitis vinifera*. The genome coverage reached to a plateau when the quota statistic was 2: 1 (in bold). Vvin = *Vitis vinifera*; and other abbreviations of species are shown in Supplementary Table 2.

Quota	Genome coverage	Hhai (X) vs Vvin (Y)	Hret (X) vs Vvin (Y)	Hchi (X) vs Vvin (Y)	Pchi (X) vs Vvin (Y)	Srob (X) vs Vvin (Y)	Dtur (X) vs Vvin (Y)	Vman (X) vs Vvin (Y)
1:1	genome X coverage (%)		68.9	58.5	57.5	58.3	57.4	57.8
	genome Y coverage (%)	95.0	94.5	95.0	95.2	96.4	95.9	96.3
**2:1**	**genome X coverage (%)**	**97.5**	**96.9**	**97.7**	**97.8**	**97.9**	**97.9**	**98.4**
	**genome Y coverage (%)**	**97.3**	**96.9**	**97.2**	**98.0**	**98.2**	**97.8**	**98.4**
3:1	genome X coverage (%)	97.6	97.1	97.9	98.0	98.2	98.2	98.5
	genome Y coverage (%)	97.1	96.7	97.2	98.0	98.2	97.8	98.3
4:1	genome X coverage (%)	97.6	97.1	97.9	98.1	98.2	98.2	98.5
	genome Y coverage (%)	97.1	96.7	97.2	98.0	98.2	97.8	98.3
6:1	genome X coverage (%)	97.6	97.1	97.9	98.1	98.2	98.2	98.5
	genome Y coverage (%)	97.1	96.7	97.2	98.0	98.2	97.8	98.3
8:1	genome X coverage (%)	97.6	97.1	97.9	98.1	98.2	98.2	98.5
	genome Y coverage (%)	97.1	96.7	97.2	98.0	98.2	97.8	98.3
3:3	genome X coverage (%)	100.0	100.0	100.0	99.9	99.9	99.9	100.0
	genome Y coverage (%)	100.0	100.0	100.0	100.0	100.0	100.0	100.0

Supplementary Table 11. KEGG categories for the positively selected genes detected in the genomes of our focal species compared with those of the five temperate tree species.

KEGG ID	Map title	N genes	KEGG ID	Map title	N genes
map03440	Homologous recombination	2	map00565	Ether lipid metabolism	1
map00053	Ascorbate and aldarate metabolism	2	map00220	Arginine biosynthesis	1
map00740	Riboflavin metabolism	1	map00073	Cutin, suberine and wax biosynthesis	1
map00232	Caffeine metabolism	1	map00400	Phenylalanine, tyrosine and tryptophan biosynthesis	1
map03018	RNA degradation	3	map00195	Photosynthesis	1
map00902	Monoterpenoid biosynthesis	1	map00010	Glycolysis / Gluconeogenesis	2
map00920	Sulfur metabolism	1	map00350	Tyrosine metabolism	1
map00071	Fatty acid degradation	1	map00240	Pyrimidine metabolism	2
map00900	Terpenoid backbone biosynthesis	1	map00360	Phenylalanine metabolism	1
map03030	DNA replication	1	map03050	Proteasome	1
map00860	Porphyrin and chlorophyll metabolism	1	map02010	ABC transporters	1
map04141	Protein processing in endoplasmic reticulum	3	map01212	Fatty acid metabolism	1
map03015	mRNA surveillance pathway	2	map01210	2-Oxocarboxylic acid metabolism	1
map00230	Purine metabolism	2	map00630	Glyoxylate and dicarboxylate metabolism	1
map03420	Nucleotide excision repair	1	map00260	Glycine, serine and threonine metabolism	1
map00760	Nicotinate and nicotinamide metabolism	1	map01230	Biosynthesis of amino acids	3
map00480	Glutathione metabolism	1	map00030	Pentose phosphate pathway	1
map00130	Ubiquinone and other terpenoid-quinone biosynthesis	2	map04146	Peroxisome	1
map00564	Glycerophospholipid metabolism	3	map00052	Galactose metabolism	1
map00730	Thiamine metabolism	1	map03008	Ribosome biogenesis in eukaryotes	1
map00650	Butanoate metabolism	1	map03040	Spliceosome	2
map00062	Fatty acid elongation	1	map00500	Starch and sucrose metabolism	3

Supplementary Table 12. Genes for *in vitro* functional assays from the genome of *H. chinensis* with substrates and expected reaction products.

Gene ID	Gene name	Substrate(s)	Expected product(s)
evm.model.000015F.76	UDP-sugar pyrophosphorylase (*USP*)	Glucose 1-phosphate, uridine triphosphate (UTP)	Diphosphate, (uridine diphosphate) UDP-glucose
evm.model.000011F.38	Pyridoxine 4-dehydrogenase (*PLR1*)	NADPH, pyridoxal	Pyridoxine, NADPH+

Supplementary Table 13. KEGG categories for the contracted gene families detected in the genomes of our focal species compared with those of the five temperate tree species.

KEGG ID	Map title	N gene families
map04626	Plant-pathogen interaction	5
map00903	Limonene and pinene degradation	1
map00909	Sesquiterpenoid and triterpenoid biosynthesis	1
map00730	Thiamine metabolism	1
map04122	Sulfur relay system	1
map00945	Stilbenoid, diarylheptanoid and gingerol biosynthesis	1
map00460	Cyanoamino acid metabolism	2
map00966	Glucosinolate biosynthesis	1
map00950	Isoquinoline alkaloid biosynthesis	2
map00960	Tropane, piperidine and pyridine alkaloid biosynthesis	2
map00360	Phenylalanine metabolism	2
map00350	Tyrosine metabolism	1
map00410	beta-Alanine metabolism	2
map00260	Glycine, serine and threonine metabolism	2
map00380	Tryptophan metabolism	1
map01210	2-Oxocarboxylic acid metabolism	1
map00940	Phenylpropanoid biosynthesis	2
map00040	Pentose and glucuronate interconversions	1

Supplementary Table 14. The significantly expanded/contracted gene families in the genomes of our focal species compared with those of five temperate tree species. NA: not available.

Gene family ID	Encoded protein	KEGG pathway/GO term	Functional category	Reference
30 (expanded)	Disease resistance proteins	GO:0006952 Defense response	Plant immunity	NA
29, 39, 65, 74, 245, 246, 257 (contracted)	Leucine-rich repeat receptor-like kinases (LRR-RLKs)	map04626 Plant-pathogen interaction	Plant immunity	23
22, 68 (contracted)	CC-NBS-LRR (NBS-LRR: nucleotide binding site-leucine rich repeat)	map04626 Plant-pathogen interaction	Plant immunity	24
5 (contracted)	TIR-NBS-LRR	map04627 Plant-pathogen interaction	Plant immunity	24

Supplementary Table 15. Expanded and contracted gene families related to plant immunity in the genomes of our focal Dipterocarpoideae species compared with those of the five temperate tree species. Abbreviations of annotated function of gene families are shown in Supplementary Table 14.

Family ID	*H. chinensis*	*P. chinensis*	*S. robusta*	*D. turbinatus*	*V. mangachapoi*	*B. pendula*	*F. excelsior*	*F. sylvatica*	*O. rehderiana*	*Q. robur*	Encoded protein
**Expanded gene families**											
30	0	13	3	256	73	0	0	0	0	0	Disease resistance proteins
**Contracted gene families**											
29	7	4	9	5	11	30	52	89	26	114	LRR-RLK-1
39	3	2	3	2	6	2	11	102	0	128	LRR-RLK-2
65	3	2	1	4	10	0	12	56	4	97	LRR-RLK-3
74	6	3	2	3	6	29	18	42	16	44	LRR-RLK-4
245	3	2	1	1	3	15	7	26	1	6	LRR-RLK-5
246	3	0	2	3	0	27	5	19	0	6	LRR-RLK-6
257	1	0	2	2	5	13	7	9	0	25	LRR-RLK-7
22	6	2	1	2	16	2	23	149	12	193	CC-NBS-LRR-1
68	1	2	0	2	0	11	9	54	25	73	CC-NBS-LRR-2
5	0	1	1	1	1	73	10	158	4	361	TIR-NBS-LRR-1

Supplementary Table 16. Statistics of re-sequencing data (using Illumina NovaSeq 6000 platform) for *H. hainanensis* and *H. reticulata*.

Species	Sample	Sampling site	Total data (Gb)	The longest monoploid genome (LMG)		The longest two monoploid genomes	
				Mapping rate (%)	Average depth (X)	Mapping rate (%)	Average depth (X)
H. hainanensis	BW1	Bawangling	25.00	85.95	55.57	94.60	33.28
	BW2	Bawangling	27.02	87.10	60.81	95.79	36.19
	BW3	Bawangling	29.31	84.13	61.82	92.64	36.86
	BW4	Bawangling	26.79	86.10	58.90	94.51	34.93
	BW5	Bawangling	26.28	76.57	49.62	84.49	29.76
	BW6	Bawangling	30.58	60.06	47.29	66.16	28.23
	BW7	Bawangling	24.61	73.76	45.23	81.40	27.09
	BW8	Bawangling	21.28	79.04	44.54	86.78	26.41
	BW9	Bawangling	24.21	84.91	54.15	93.36	32.17
	BW10	Bawangling	26.34	81.40	54.34	89.87	32.50
	BW11	Bawangling	25.30	71.53	47.34	78.71	28.29
	BW12	Bawangling	36.09	63.08	59.62	69.28	36.75
	BW13	Bawangling	25.70	66.40	44.45	73.04	26.71
	BW14	Bawangling	36.06	81.14	75.24	89.02	46.30
	BW15	Bawangling	27.22	85.75	59.35	92.82	34.70
	BW16	Bawangling	36.76	87.14	79.51	95.68	47.30
	BW17	Bawangling	27.22	84.14	58.40	94.32	37.24
	BW18	Bawangling	22.85	84.25	48.11	92.70	29.09
	BW19	Bawangling	29.27	84.90	64.70	93.44	38.34
	BW20	Bawangling	25.91	84.34	55.44	92.68	33.62
	BW21	Bawangling	27.79	79.03	55.90	86.87	33.28
	BW22	Bawangling	26.37	85.71	57.78	94.23	34.41
	BW23	Bawangling	22.80	85.57	50.94	93.82	30.20

	DZ1	Danzhou	26.71	85.31	58.42	93.65	34.68
	JF1	Jianfengling	26.25	86.31	56.46	94.74	35.05
	JF2	Jianfengling	29.80	68.13	52.58	74.79	32.57
	JF3	Jianfengling	30.61	85.05	64.52	93.64	39.72
	JF4	Jiangfengling	33.99	60.47	53.32	66.40	33.02
	JF5	Jianfengling	26.73	76.68	52.78	84.53	31.42
	WZ1	Wuzhi	25.35	63.56	41.86	69.87	25.05
H. reticulata	GS1	Ganshiling	27.19	0.81	47.95	89.81	30.36
	GS2	Ganshiling	43.68	0.82	80.63	90.81	50.61
	GS3	Ganshiling	38.15	0.83	66.90	91.66	41.99
	GS4	Ganshiling	33.22	0.78	56.43	85.81	35.70
	GS5	Ganshiling	28.25	0.73	45.49	80.72	28.79
	GS6	Ganshiling	26.84	0.84	49.34	93.25	31.24
	GS7	Ganshiling	35.30	0.83	62.42	92.14	39.39
	GS8	Ganshiling	30.05	0.85	55.68	93.40	35.13
	GS9	Ganshiling	31.42	0.83	55.94	91.27	35.35
	GS10	Ganshiling	30.09	0.84	54.71	92.39	34.49
	GS11	Ganshiling	28.64	0.84	51.09	92.55	32.40
	GS12	Ganshiling	26.96	0.84	49.77	92.68	31.49
	GS13	Ganshiling	29.85	0.82	53.06	90.98	33.52
	GS14	Ganshiling	27.72	0.80	48.44	87.96	30.94
	GS15	Ganshiling	29.33	0.80	50.09	88.73	31.54
	GS16	Ganshiling	30.27	0.84	55.23	92.84	35.05
	GS17	Ganshiling	26.92	0.80	46.91	88.56	29.79
	GS18	Ganshiling	31.38	0.83	55.45	91.93	35.12
	GS19	Ganshiling	26.47	0.82	47.31	90.96	30.05
	GS20	Ganshiling	28.18	0.73	44.93	81.05	28.72

GS21	Ganshiling	44.62	0.82	77.39	90.66	48.58
GS22	Ganshiling	36.48	0.76	58.45	84.21	36.91
GS23	Ganshiling	25.22	0.79	44.50	87.57	28.32
GS24	Ganshiling	26.04	0.81	45.80	89.36	29.20
GS25	Ganshiling	27.35	0.78	44.52	85.90	28.50
GS26	Ganshiling	25.55	0.77	44.09	85.57	28.07
GS27	Ganshiling	26.06	0.66	37.87	73.10	24.34
GS28	Ganshiling	28.91	0.84	52.65	92.91	33.36
GS29	Ganshiling	29.15	0.80	50.70	88.88	32.23
GS30	Ganshiling	25.65	0.84	46.43	92.56	29.45
GS31	Ganshiling	26.03	0.80	45.47	88.65	28.84
GS32	Ganshiling	27.82	0.69	42.22	76.14	26.95

Supplementary Table 17. Categories of SNPs based on genome annotation. Upstream: SNP located within the 1Kb upstream region of a gene. Exonic: SNP located in an exon. Synonymous: SNP that did not caused change of encoded amino acid. Stop gain: SNP that caused encoded amino acid to become a stop codon. Stop loss: SNP that caused loss of a stop codon. Start loss: SNP that caused loss of a start codon. Non-synonymous: SNP that caused change of encoded amino acid. Intronic: SNP located in an intron. Splicing: SNP located in an alternative splicing site. Downstream: SNP located within the 1Kb downstream region of a gene. Upstream/Downstream: SNP that located within the 1Kb upstream region of a gene, at the same time, located within the 1Kb downstream region of another gene. Intergenic: SNP located in an intergenic region. Total: total number of SNPs.

Category		Number of SNPs in *H. hainanensis*	Number of SNPs in *H. reticulata*
**The longest monoploid genome**			
Upstream		207,657	299,880
Exonic	Stop gain	1,672	2,346
Exonic	Stop loss	351	361
Exonic	Start loss	272	332
Exonic	Non-synonymous	109,666	168,449
Intronic		362,658	563,231
Splicing		838	1,372
Downstream		186,002	274,106
Upstream/Downstream		22,165	27,259
Intergenic		1,942,417	2,995,481
Total		2,922,653	4,477,756
**The longest two monoploid genomes**			
	Upstream	102,324	179,170
Exonic	Stop gain	959	1,601
Exonic	Stop loss	198	235
Exonic	Synonymous	48,434	89,220
Exonic	Non-synonymous	60,174	107,962
Intronic		214,885	369,200
Splicing		518	944
Downstream		92,121	166,974
upstream/downstream		15,342	17,970
Intergenic		1,006,070	2,076,211
Total		1,582,562	3,028,610

Supplementary Table 18. Estimation of mutation rate for the genomes of *H. hainanensis* and *H. reticulata*.

	H. hainanensis vs. *H. reticulata*	*H. hainanensis* vs. *H. chinensis*	*H. reticulata* vs. *H. chinensis*
All fourfold degenerate sites	562034	920297	710925
Diverged fourfold degenerate sites	21841	33788	17475
Mutation rate (/site/generation)	1.06×10^{-8} (generation time setting as 15 years)		
	1.41×10^{-8} (generation time setting as 20 years)		
	2.11×10^{-8} (generation time setting as 30 years)		

Supplementary Table 19. Results of KEGG enrichment analysis for the 647 (in *H. hainanensis*) and 581 genes (in *H. reticulata*) with derived deleterious mutations (DDMs) and for the 407 (in *H. hainanensis*) and 300 genes (in *H. reticulata*) with derived major-effect mutations (DMEMs). Fisher's Chi-Square test was used to identify pathways with significant enrichment results. We only present the pathways with significant results in KEGG enrichment analysis.

**Genes with DDMs**				**Genes with DMEMs**			
KEGG pathway	Description	Gene number	*P* value	KEGG pathway	Description	Gene number	*P* value
H. hainanensis							
map00592	alpha-Linolenic acid metabolism	8	0.0035	map04145	Phagosome	6	0.0297
map00944	Flavone and flavonol biosynthesis	3	0.0097	map03410	Base excision repair	4	0.0397
map00020	Citrate cycle (TCA cycle)	6	0.0322	map00730	Thiamine metabolism	2	0.0484
map00591	Linoleic acid metabolism	3	0.0365				
map00051	Fructose and mannose metabolism	9	0.0457				
map00564	Glycerophospholipid metabolism	12	0.0484				
H. reticulata							
map01502	Vancomycin resistance	2	0.0015	map00944	Flavone and flavonol biosynthesis	2	0.0078
map00052	Galactose metabolism	10	0.0047	map03008	Ribosome biogenesis in eukaryotes	6	0.0203
map00970	Aminoacyl-tRNA biosynthesis	10	0.0063	map01502	Vancomycin resistance	1	0.0335
map01210	2-Oxocarboxylic acid metabolism	7	0.0196	map00052	Galactose metabolism	5	0.0419
map00053	Ascorbate and aldarate metabolism	5	0.0242				
map00565	Ether lipid metabolism	4	0.0350				

Supplementary Table 20. Results of KEGG enrichment analysis for the 195 (in *H. hainanensis*) and 195 genes (in *H. reticulata*) with the DDMs that were homozygous in at least one sampled tree and for the 175 (in *H. hainanensis*) and 150 genes (in *H. reticulata*) with DMEMs that were homozygous in at least one sampled tree. Fisher's Chi-Square test was used to identify pathways with significant enrichment results. We only present the pathways with significant results in KEGG enrichment analysis.

**Genes with homozygous DDMs**				**Genes with homozygous DMEMs**			
KEGG pathway	Description	Gene number	*P* value	KEGG pathway	Description	Gene number	*P* value
H. hainanensis							
map00500	Starch and sucrose metabolism	11	0.0044	map00950	Isoquinoline alkaloid biosynthesis	2	0.0307
map00591	Linoleic acid metabolism	2	0.0181	map00960	Tropane, piperidine and pyridine alkaloid biosynthesis	2	0.0370
map00592	alpha-Linolenic acid metabolism	3	0.0332	map00260	Glycine, serine and threonine metabolism	3	0.0395
map00940	Phenylpropanoid biosynthesis	5	0.0374	map00565	Ether lipid metabolism	2	0.0464
				map00562	Inositol phosphate metabolism	3	0.0476
H. reticulata							
map00052	Galactose metabolism	6	0.0012	map00944	Flavone and flavonol biosynthesis	2	0.0019
map00500	Starch and sucrose metabolism	10	0.0044	map00230	Purine metabolism	5	0.0228
map01502	Vancomycin resistance	1	0.0210	map00232	Caffeine metabolism	1	0.0375
map00942	Anthocyanin biosynthesis	1	0.0484				
map00460	Cyanoamino acid metabolism	3	0.0486				

Supplementary Table 21. Sampled tissues and types and data size of transcriptome sequencing for the prediction of gene models.

Species	Sampled tissue	Sequencing type	Sequencing data (Gb)
H. hainanensis	Roots, stems, leaves from seedlings; leaves, flowers of 3 different development stages and fruits of 5 different development stages from mature trees	Pacbio Iso-seq	46.26
	Flowers of 3 different development stages and fruits of 4 different development stages	Illumina RNA-seq	63.19
H. reticulata	Roots, stems, leaves from seedlings	Pacbio Iso-seq	34.54
H. chinensis	Leaves from one mature individual	Illumina RNA-seq	6.80
P. chinensis	Stems and leaves from one sapling	Illumina RNA-seq	20.76
S. robusta	Stems and leaves from one mature individual; stems and Leaves from several seedlings	Illumina RNA-seq	42.73
D. turbinatus	Leaves from one sapling	Illumina RNA-seq	10.62
V. mangachapoi	Stems and leaves from one mature individual	Illumina RNA-seq	21.47

References

1. Ashton PS. In *Flora Malesiana* (ed. van Steenis, CGGJ) 237–552 (Martinus Nijhoff, Dordrecht, 1982).
2. Bell CD, Soltis DE, Soltis PS. The age and diversification of the angiosperms re-revisited. *American Journal of Botany* **97**, 1296–1303 (2010).
3. Vega FJ, García-Barrera P, Perrilliat MdC, Coutiño MA, Mariño-Pérez R. El Espinal, a new plattenkalk facies locality from the Lower Cretaceous Sierra Madre Formation, Chiapas, southeastern Mexico. *Revista Mexicana de Ciencias Geológicas* **23**, 323–333 (2006).
4. Bansal M*, et al.* Southeast Asian Dipterocarp origin and diversification driven by Africa-India floristic interchange. *Science* **375**, 455–460 (2022).
5. Ng KKS*, et al.* The genome of *Shorea leprosula* (Dipterocarpaceae) highlights the ecological relevance of drought in aseasonal tropical rainforests. *Communications Biology* **4**, 1166 (2021).
6. Yang YZ*, et al.* Genomic effects of population collapse in a critically endangered ironwood tree *Ostrya rehderiana*. *Nature Communications* **9**, 5449 (2018).
7. Duan NB*, et al.* Genome re-sequencing reveals the history of apple and supports a two-stage model for fruit enlargement. *Nature Communications* **8**, 249 (2017).
8. Huang J*, et al.* The jujube genome provides insights into genome evolution and the domestication of sweetness/acidity taste in fruit trees. *PLoS Genetics* **12**, e1006433 (2016).
9. Salojärvi J*, et al.* Genome sequencing and population genomic analyses provide insights into the adaptive landscape of silver birch. *Nature Genetics* **49**, 904–912 (2017).
10. Verde I*, et al.* The high-quality draft genome of peach (*Prunus persica*) identifies unique patterns of genetic diversity, domestication and genome evolution. *Nature Genetics* **45**, 487–494 (2013).
11. Wang J, Street NR, Scofield DG, Ingvarsson PK. Variation in linked selection and recombination drive genomic divergence during allopatric speciation of European and American aspens. *Molecular Biology and Evolution* **33**, 1754–1767 (2016).
12. Zhao YP*, et al.* Resequencing 545 ginkgo genomes across the world reveals the evolutionary history of the living fossil. *Nature Communications* **10**, 4201 (2019).
13. Tian ZZ*, et al.* Thirteen Dipterocarpoideae genomes provide insights into their evolution and borneol biosynthesis. *Plant Communications* **3**, 100464 (2022).
14. Wang MJ, *et al*. Genomic innovation and regulatory rewiring during evolution of the cotton genus *Gossypium*. *Nature Genetics* **54**, 1959–1971 (2022).
15. Argout X*, et al.* The genome of *Theobroma cacao*. *Nature Genetics* **43**, 101–108 (2011).
16. Goff SA*, et al.* A draft sequence of the rice genome (*Oryza sativa* L. ssp. *japonica*). *Science* **296**, 92–100 (2002).
17. Kaul S*, et al.* Analysis of the genome sequence of the flowering plant *Arabidopsis thaliana*. *Nature* **408**, 796–815 (2000).
18. Ding X*, et al.* Genome sequence of the agarwood tree *Aquilaria sinensis* (Lour.) Spreng: the first chromosome-level draft genome in the Thymelaeceae family. *GigaScience* **9**, giaa013 (2020).
19. Albert VA*, et al.* The *Amborella* genome and the evolution of flowering plants. *Science* **342**, 1241089 (2013).

20. Sollars ESA*, et al.* Genome sequence and genetic diversity of European ash trees. *Nature* **541**, 212–216 (2017).
21. Mishra B*, et al.* A reference genome of the European beech (*Fagus sylvatica* L.). *GigaScience* **7**, giy063 (2018).
22. Plomion C*, et al.* Oak genome reveals facets of long lifespan. *Nature Plants* **4**, 440–452 (2018).
23. Beck M, Heard W, Mbengue M, Robatzek S. The INs and OUTs of pattern recognition receptors at the cell surface. *Current Opinion in Plant Biology* **15**, 367–374 (2012).
24. Cui HT, Tsuda K, Parker JE. Effector-triggered immunity: from pathogen perception to robust defense. *Annual Review of Plant Biology* **66**, 487–511 (2015).
