# Supplementary material for: Dipterocarpoidae genomics reveal their demography and adaptations to Asian rainforests
Source: Nat Commun. 2024 Feb 23;15:1683. doi: 10.1038/s41467-024-45836-5 (PMC10891123; doi:10.1038/s41467-024-45836-5)
Supplement: Supplementary file 3 — Description of Additional Supplementary Files [file 41467_2024_45836_MOESM3_ESM.pdf]

Description of Additional Supplementary Files

File Name: Supplementary Data 1

Description: GO categories for the positively selected genes detected in the genomes of our focal species compared with those of the five temperate tree species.

File Name: Supplementary Data 2

Description: The positively selected genes supported by the comparisons with temperate trees. The gene IDs of positively selected genes are the gene IDs of the orthologs in *H. chinensis* genome. The positively selected genes associated with plants' adaptation to environmental stresses were identified by checking the published literatures, and thus their functional categories, potentially associated stress and related references were listed. NA: not available.
