# Supplementary material for: Dipterocarpoidae genomics reveal their demography and adaptations to Asian rainforests
Source: Nat Commun. 2024 Feb 23;15:1683. doi: 10.1038/s41467-024-45836-5 (PMC10891123; doi:10.1038/s41467-024-45836-5)
Supplement: Supplementary file 4 — Supplementary Data 1 [file 41467_2024_45836_MOESM4_ESM.pdf]

Supplementary Data 1. GO categories for the positively selected genes detected in the genomes of our focal species compared with those of the five temperate tree species.

GO ID	GO term	N genes	GO ID	GO term	N genes
GO:0006281	DNA repair	3	GO:0009060	Aerobic respiration	1
GO:0000724	Double-strand break repair via homologous recombination	1	GO:2000028	Regulation of photoperiodism, flowering	1
GO:0006289	Nucleotide-excision repair	1	GO:0006662	Glycerol ether metabolic process	1
GO:0006979	Response to oxidative stress	3	GO:0048523	Negative regulation of cellular process	2
GO:0045454	Cell redox homeostasis	2	GO:0019062	Virion attachment to host cell	2
GO:0019725	Cellular homeostasis	3	GO:0030833	Regulation of actin filament polymerization	1
GO:0009451	RNA modification	3	GO:0051128	Regulation of cellular component organization	2
GO:0006011	UDP-glucose metabolic process	3	GO:0016998	Cell wall macromolecule catabolic process	1
GO:0033567	DNA replication, Okazaki fragment processing	1	GO:0006259	DNA metabolic process	14
GO:0038032	Termination of G-protein coupled receptor signaling pathway	1	GO:0016049	Cell growth	2
GO:0000162	Tryptophan biosynthetic process	1	GO:0019835	Cytolysis	1
GO:0001522	Pseudouridine synthesis	2	GO:0009617	Response to bacterium	1
GO:0006631	Fatty acid metabolic process	4	GO:0001503	Ossification	1
GO:0045132	Meiotic chromosome segregation	1	GO:0007010	Cytoskeleton organization	4
GO:0008616	Queuosine biosynthetic process	1	GO:0030100	Regulation of endocytosis	1
GO:0006438	Valyl-tRNA aminoacylation	1	GO:0071822	Protein complex subunit organization	7
GO:0009405	Pathogenesis	10	GO:0006002	Fructose 6-phosphate metabolic process	1
GO:0051704	Multi-organism process	13	GO:0007194	Negative regulation of adenylate cyclase activity	1
GO:0000280	Nuclear division	2	GO:0006265	DNA topological change	4

GO:0022402	Cell cycle process	4	GO:0006399	tRNA metabolic process	2
GO:0000003	Reproduction	2	GO:0006996	Organelle organization	9
GO:0006584	Catecholamine metabolic process	1	GO:0016539	Intein-mediated protein splicing	3
GO:0006788	Heme oxidation	1	GO:0044710	Single-organism metabolic process	39
GO:1901615	Organic hydroxy compound metabolic process	2	GO:0055114	Oxidation-reduction process	20
GO:0032787	Monocarboxylic acid metabolic process	6	GO:0006461	Protein complex assembly	6
GO:0006691	Leukotriene metabolic process	1	GO:0044780	Bacterial-type flagellum assembly	1
GO:2000026	Regulation of multicellular organismal development	2	GO:0046394	Carboxylic acid biosynthetic process	3
GO:0006275	Regulation of DNA replication	1	GO:0044281	Small molecule metabolic process	16
GO:0044255	Cellular lipid metabolic process	5	GO:0007275	Multicellular organismal development	3
GO:0032940	Secretion by cell	4	GO:0015886	Heme transport	1
GO:0016525	Negative regulation of angiogenesis	1	GO:0090066	Regulation of anatomical structure size	3
GO:0007342	Fusion of sperm to egg plasma membrane	1	GO:0017004	Cytochrome complex assembly	1
GO:0008154	Actin polymerization or depolymerization	2	GO:0016043	Cellular component organization	12
GO:0006396	RNA processing	4	GO:0016055	Wnt signaling pathway	1
GO:0009306	Protein secretion	3	GO:0009401	Phosphoenolpyruvate-dependent sugar phosphotransferase system	1
GO:0006904	Vesicle docking involved in exocytosis	1	GO:0006091	Generation of precursor metabolites and energy	4
GO:0030042	Actin filament depolymerization	1	GO:0070493	Thrombin receptor signaling pathway	1
GO:0001505	Regulation of neurotransmitter levels	2	GO:0015672	Monovalent inorganic cation transport	13
GO:0032259	Methylation	2	GO:0006352	DNA-templated transcription, initiation	1
GO:0016180	snRNA processing	1	GO:0006470	Protein dephosphorylation	1
GO:0034470	ncRNA processing	2	GO:0006915	Apoptotic process	1
GO:0006629	Lipid metabolic process	7	GO:0005975	Carbohydrate metabolic process	10
GO:0006457	Protein folding	3	GO:0015031	Protein transport	5

GO:0048519	Negative regulation of biological process	3	GO:0030001	Metal ion transport	16
GO:0055117	Regulation of cardiac muscle contraction	1	GO:0022607	Cellular component assembly	7
GO:0051225	Spindle assembly	2	GO:0044262	Cellular carbohydrate metabolic process	2
GO:0007067	Mitotic nuclear division	1	GO:0043623	Cellular protein complex assembly	2
GO:0070925	Organelle assembly	3	GO:0048583	Regulation of response to stimulus	2
GO:0051297	Centrosome organization	2	GO:0019752	Carboxylic acid metabolic process	9
GO:0051239	Regulation of multicellular organismal process	4	GO:0006096	Glycolytic process	2
GO:0006260	DNA replication	4	GO:0016192	Vesicle-mediated transport	2
GO:0005978	Glycogen biosynthetic process	1	GO:0070588	Calcium ion transmembrane transport	2
GO:0019229	Regulation of vasoconstriction	2	GO:0034622	Cellular macromolecular complex assembly	4
GO:0006813	Potassium ion transport	12	GO:0051649	Establishment of localization in cell	5
GO:0006633	Fatty acid biosynthetic process	2	GO:0055086	Nucleobase-containing small molecule metabolic process	5
GO:0022414	Reproductive process	2	GO:0071103	DNA conformation change	6
GO:0042119	Neutrophil activation	1	GO:0044711	Single-organism biosynthetic process	7
GO:0006306	DNA methylation	1	GO:0065008	Regulation of biological quality	10
GO:0007217	Tachykinin receptor signaling pathway	1	GO:0006812	Cation transport	17
GO:0015979	Photosynthesis	2	GO:0006811	Ion transport	23
GO:0008610	Lipid biosynthetic process	3	GO:0044763	Single-organism cellular process	71
GO:0034660	ncRNA metabolic process	3	GO:1901135	Carbohydrate derivative metabolic process	6
GO:0006066	Alcohol metabolic process	1	GO:0007204	Positive regulation of cytosolic calcium ion concentration	1
GO:0008299	Isoprenoid biosynthetic process	1	GO:0048731	System development	2
GO:1902589	Single-organism organelle organization	5	GO:0015980	Energy derivation by oxidation of organic compounds	2
GO:0006836	Neurotransmitter transport	1			
