# Supplementary material for: Dipterocarpoidae genomics reveal their demography and adaptations to Asian rainforests
Source: Nat Commun. 2024 Feb 23;15:1683. doi: 10.1038/s41467-024-45836-5 (PMC10891123; doi:10.1038/s41467-024-45836-5)
Supplement: Supplementary file 5 — Supplementary Data 2 [file 41467_2024_45836_MOESM5_ESM.pdf]

Supplementary Data 2. The positively selected genes supported by the comparisons with temperate trees. The gene IDs of positively selected genes are the gene IDs of the orthologs in *H. chinensis* genome. The positively selected genes associated with plants' adaptation to environmental stresses were identified by checking the published literatures, and thus their functional categories, potentially associated stress and related references were listed. NA: not available.

GENE_ID	Encoded protein	KEGG pathway /GO term	Functional category	Associated stress	Reference
evm.model.000008F.338	DNA repair protein RAD51 homolog 4 (RAD51D)	map03440 Homologous recombination	DNA repair	UV adaptation	1
evm.model.000183F.61	Structural maintenance of chromosomes protein 5 (SMC5)	GO:0006281 DNA repair	DNA repair	UV adaptation	2
evm.model.000264F.14	Carotenoid ε-hydroxylase (LUT1)	map00906 Carotenoid biosynthesis	DNA repair	Drought tolerance	3
evm.model.000060F.10	DNA replication ATP-dependent helicase Dna2 (DNA2)	map03030 DNA replication	DNA repair	Drought tolerance<br>Salt tolerance	4
evm.model.000223F.16	3'(2'), 5'-bisphosphate nucleotidase (cysQ)	map00920 Sulfur metabolism	DNA repair	Drought tolerance	5
evm.model.000026F.212	Bloom syndrome protein (BLM)	map03440 Homologous recombination<br>GO:0006281 DNA repair	DNA repair	UV adaptation	1
evm.model.000196F.15	Leucine-rich PPR motif-containing protein, mitochondrial (LRPPRC)	GO:0006281 DNA repair	DNA repair	UV adaptation	2
evm.model.000015F.398	Lysine-specific histone demethylase 1A (LSD1)		DNA repair	UV adaptation	6
evm.model.000014F.58	Serine/Threonine-protein kinase SMG1 (SMG1)	map03015 mRNA surveillance pathway	Abnormal mRNA degradation	Drought tolerance	7

evm.model.000015F.420.3	Serine/threonine-protein phosphatase 2A regulatory subunit B' (PPP2R5)	map03015 mRNA surveillance pathway	Abnormal mRNA degradation	Heat tolerance	8
evm.model.000293F.15.5	Superkiller protein 3 (SKI3)	map03018 RNA degradation	Abnormal mRNA degradation	Drought tolerance	9
evm.model.000260F.28	Riboflavin kinase (RFK)	map00740 Riboflavin metabolism	Antioxidation	Drought tolerance	10
evm.model.000088F.155	L-ascorbate peroxidase (APX)	map00053 Ascorbate and aldarate metabolism	Antioxidation	Heat tolerance<br>Flooding tolerance<br>Salt tolerance<br>Drought tolerance	11
evm.model.000258F.15	Xanthine dehydrogenase/oxidase (XDH)	map00232 Caffeine metabolism<br>map00230 Purine metabolism	Antioxidation	UV adaptation	12
evm.model.000081F.77.3	Geranyl diphosphate synthase (GPS)	map00900 Terpenoid backbone biosynthesis	Antioxidation	UV adaptation	13
evm.model.000015F.76	UDP-sugar pyrophosphorylase (USP)	map00052 Galactose metabolism<br>map00053 Ascorbate and aldarate metabolism	Antioxidation	Drought tolerance<br>Heat tolerance<br>Salt tolerance	14
evm.model.000089F.29	Alcohol dehydrogenase (ADH)	map00071 Fatty acid degradation	Anaerobic respiration enhancement	Flooding tolerance	15
evm.model.000190F.19	Peptide-N4-(N-acetyl-beta-glucosaminyl)asparagine amidase (NGLY1)	map04141 Protein processing in endoplasmic reticulum	ERAD (Endoplasmic Reticulum Associated Degradation) pathway	Heat tolerance	16

evm.model.000059F.36	Neomenthol dehydrogenase (NDH)	map00902 Monoterpenoid biosynthesis	Pathogen resistance	Drought tolerance	17
evm.model.000072F.45	Molecular chaperone GrpE (GRPE)		Cellular energy homeostasis	Heat tolerance	18
evm.model.000237F.34	Glucan endo-1,3-beta-glucosidase 4 (GN4)	map00500 Starch and sucrose metabolism			
evm.model.000090F.132	Endoglucanase	map00500 Starch and sucrose metabolism			
evm.model.000007F.357	Glucan endo-1,3-beta-glucosidase 1/2/3	map00500 Starch and sucrose metabolism			
evm.model.000022F.77	U5 snRNP protein, DIM1 family	map03040 Spliceosome			
evm.model.000087F.26	Crooked neck (CRN)	map03040 Spliceosome			
evm.model.000014F.222	3-Hydroxybutyryl-CoA dehydrogenase	map00360 Phenylalanine metabolism<br>map00650 Butanoate metabolism			
evm.model.000002F.161	Fatty acid omega-hydroxylase	map00073 Cutin, suberine and wax biosynthesis			
evm.model.000172F.35	U3 small nucleolar RNA-associated protein MPP10 (MPP10)	map03008 Ribosome biogenesis in eukaryotes			
evm.model.000081F.131	Acyl-activating enzyme 14 (AAE14)	map00130 Ubiquinone and other terpenoid-quinone biosynthesis			
evm.model.000094F.123	NAD(P)H dehydrogenase (quinone) (wrbA)	map00130 Ubiquinone and other terpenoid-quinone biosynthesis			
evm.model.000013F.165	SEL1 protein (SEL1)	map04141 Protein processing in endoplasmic reticulum			
evm.model.000173F.72	Palmitoyl-protein thioesterase (PPT)	map00062 Fatty acid elongation			

evm.model.000045F.23	Monolysocardiolipin acyltransferase	map00564 Glycerophospholipid metabolism
evm.model.000024F.309	Uracil phosphoribosyltransferase	map00240 Pyrimidine metabolism
evm.model.000254F.36	ATP-binding cassette, subfamily G (WHITE), member 2	map02010 ABC transporters
evm.model.000107F.89.2	2-Dehydro-3-deoxyphosphogluconate aldolase / (4S)-4-hydroxy-2-oxoglutarate aldolase	map00630 Glyoxylate and dicarboxylate metabolism
evm.model.000008F.209	Enolase	map01230 Biosynthesis of amino acids<br>map00010 Glycolysis / Gluconeogenesis<br>map03018 RNA degradation
evm.model.000148F.47	5'-Nucleotidase	map00760 Nicotinate and nicotinamide metabolism<br>map00240 Pyrimidine metabolism
evm.model.000042F.132	Derlin-2/3	map04141 Protein processing in endoplasmic reticulum
evm.model.000012F.28	Phospholipase D3/4	map00565 Ether lipid metabolism<br>map00564 Glycerophospholipid metabolism
evm.model.000244F.55	photosystem I subunit III	map00195 Photosynthesis
evm.model.000019F.19	Aminoacylase	map00220 Arginine biosynthesis<br>map01230 Biosynthesis of amino acids
evm.model.000003F.372	Tryptophan synthase beta chain	map00400 Phenylalanine, tyrosine and tryptophan biosynthesis<br>map01230 Biosynthesis of amino acids

evm.model.000186F.18	Monolysocardiolipin acyltransferase	map00564 Glycerophospholipid metabolism
evm.model.000081F.76.1	26S proteasome regulatory subunit T4	map03050 Proteasome
evm.model.000294F.13	Thiamine pyrophosphokinase	map00730 Thiamine metabolism
evm.model.000049F.44	DNA excision repair protein ERCC-4	map03420 Nucleotide excision repair
evm.model.000125F.114	Heme oxygenase (biliverdin-producing) (HMOX1)	map00860 Porphyrin and chlorophyll metabolism
evm.model.000060F.241	BRCA1-A complex subunit BRE (BRE)	NA
evm.model.000001F.21	Histone deacetylase 10 (HDAC10)	NA
evm.model.000141F.13	PAX-interacting protein 1 (PAXIP1)	NA
evm.model.000119F.12	Disease resistance protein RPM1 (RPM1)	NA
evm.model.000048F.9	F-box protein GID2	NA
evm.model.000016F.397	Integrator complex subunit 9	NA
evm.model.000070F.185	Magnesium/proton exchanger	NA
evm.model.000116F.31	Rab3 GTPase-activating protein catalytic subunit (RAB3GAP1)	NA
evm.model.000008F.314	4-nitrophenyl phosphatase	NA
evm.model.000137F.78	Cyclin D5, plant (CYCD5)	NA
evm.model.000020F.115	FK506-binding nuclear protein (FPR3_4)	NA

evm.model.000264F.25	Peptidylprolyl isomerase	NA
evm.model.000084F.131	ATP-dependent DNA helicase HFM1/MER3	NA
evm.model.000264F.21	DnaJ homolog subfamily C member 28	NA
evm.model.000118F.96	ATP-dependent RNA helicase DDX43 (DDX43)	NA
evm.model.000108F.43	Palmitoyltransferase ZDHHC2/15/20 (ZDHHC2_15_20)	NA
evm.model.000185F.65	Gamma-glutamylaminecyclotransferase (GGACT)	NA
evm.model.000015F.73	Cohesin complex subunit SCC1 (SCC1)	NA
evm.model.000010F.29	Aminoacyl tRNA synthase complex-interacting multifunctional protein 1	NA
evm.model.000038F.139	Leucine-rich PPR motif-containing protein, mitochondrial (LRPPRC)	NA
evm.model.000075F.43	Sterol 3beta-glucosyltransferase	NA
evm.model.000030F.147.1	Pre-mRNA-processing factor 39	NA
evm.model.000075F.98	Proteasome assembly chaperone 2	NA
evm.model.000003F.141	Queuine tRNA-ribosyltransferase accessory subunit (QTRT2)	NA
evm.model.000154F.6	Protein N-terminal asparagine amidohydrolase	NA

evm.model.000118F.87	ATP-dependent NAD(P)H-hydrate dehydratase	NA
evm.model.000003F.336	GINS complex subunit 4 (GINS4)	NA
evm.model.000055F.138	E3 ubiquitin-protein ligase Topors	NA
evm.model.000049F.127	ATP-dependent RNA helicase DDX59	NA
evm.model.000132F.39	Pentatricopeptide repeat domain-containing protein 1	NA
evm.model.000091F.131	Endothelin-converting enzyme (ECE)	NA
evm.model.000194F.53	Interleukin-1 receptor-associated kinase 1	NA
evm.model.000179F.13	Uncharacterized protein	NA
evm.model.000188F.46	Chromosome transmission fidelity protein 8	NA
evm.model.000022F.124	Myotubularin-related protein 5/13	NA
evm.model.000120F.84	Urease accessory protein	NA
evm.model.000276F.21	Tatd dnase family protein	NA
evm.model.000221F.68	Endophilin-a	NA
evm.model.000102F.42	Monothiol glutaredoxin	NA
evm.model.000141F.89_evm.model.000141F.90	Histone-binding protein rbbp4	NA
evm.model.000410F.6	Calcium-binding protein cml	NA
evm.model.000122F.63	Rna-binding protein fus	NA
evm.model.000015F.363	Sperm-associated antigen 1	NA

evm.model.000267F.31	Solute carrier family 25, member 44	NA
evm.model.000125F.121	Acylaminoacyl-peptidase	NA
evm.model.000196F.4	Leucine-rich ppr motif-containing protein, mitochondrial	NA
evm.model.000022F.104	Tetraspanin-5	NA
evm.model.000200F.10	Thioredoxin 1	NA
evm.model.000003F.254	Atp-dependent clp protease, protease subunit	NA
evm.model.000063F.79	Uncharacterized protein	NA
evm.model.000172F.60	Rna-binding protein 5/10	NA
evm.model.000062F.6	Aarf domain-containing kinase	NA
evm.model.000277F.51	Inhibitor of nuclear factor kappa-b kinase subunit alpha	NA
evm.model.000194F.81	Protein phosphatase	NA
evm.model.000102F.73	Hiv tat-specific factor 1	NA
evm.model.000044F.132	Uncharacterized protein	NA
evm.model.000049F.87.1	Putative mfs transporter, agza family, xanthine/uracil permease	NA
evm.model.000041F.137	Transcription elongation factor s-ii	NA
evm.model.000306F.17	Uncharacterized protein	NA
evm.model.000170F.79	Nadh dehydrogenase (ubiquinone) 1 alpha/beta subcomplex 1	NA
evm.model.000100F.101_ evm.model.000100F.102	General transcription factor 3c polypeptide 5 (transcription factor c subunit 1)	NA

evm.model.000013F.88	Regulator of g-protein signaling	NA
evm.model.000016F.414	Pentatricopeptide repeat domain-containing protein 1	NA
evm.model.000212F.8	Selenium-binding protein 1	NA
evm.model.000282F.12	Elongator complex protein 3	NA
evm.model.000453F.9	Solute carrier family 32 (vesicular inhibitory amino acid transporter)	NA
evm.model.000015F.451	U3 small nucleolar rna-associated protein 7	NA
evm.model.000101F.17	General transcription factor 3c polypeptide 4	NA
evm.model.000166F.93	Nephrocystin-3	NA
evm.model.000022F.226	Atp-binding protein involved in chromosome partitioning	NA
evm.model.000067F.166	Saga-associated factor 29	NA
evm.model.000081F.119	Release factor glutamine methyltransferase	NA
evm.model.000317F.9	Peroxidase	NA
evm.model.000016F.344	Eri1 exoribonuclease 2	NA
evm.model.000173F.38	Taspase, threonine aspartase, 1	NA
evm.model.000152F.40	5'-amp-activated protein kinase, regulatory gamma subunit	NA
evm.model.000023F.72	Syntaxin-binding protein 5	NA
evm.model.000195F.42	Nadh dehydrogenase	NA
evm.model.000006F.4	Tubby-related protein 1	NA

evm.model.000018F.66.1	Trna wybutosine-synthesizing protein 3	NA
evm.model.000016F.232	Uncharacterized protein	NA
evm.model.000006F.162	Trna pseudouridine38-40 synthase	NA
evm.model.000003F.62	Trna pseudouridine38-40 synthase	NA
evm.model.000120F.33	Tubulin--tyrosine ligase-like protein 12	NA
evm.model.000072F.57	Tata-binding protein-associated factor	NA
evm.model.000054F.39	NA	NA
evm.model.000068F.63	NA	NA
evm.model.000254F.45	NA	NA
evm.model.000012F.309	NA	NA
evm.model.000172F.29	NA	NA
evm.model.000072F.54.1	NA	NA
evm.model.000079F.88	NA	NA
evm.model.000095F.60	NA	NA
evm.model.000026F.310	NA	NA
evm.model.000145F.20	NA	NA
evm.model.000023F.11	NA	NA
evm.model.000216F.21	NA	NA
evm.model.000160F.69	NA	NA
evm.model.000168F.125	NA	NA
evm.model.000151F.59	NA	NA
evm.model.000152F.10	NA	NA
evm.model.000065F.121	NA	NA

evm.model.000145F.26	NA	NA
evm.model.000108F.158	NA	NA
evm.model.000045F.102	NA	NA
evm.model.000016F.456	NA	NA
evm.model.000067F.36	NA	NA
evm.model.000057F.74	NA	NA
evm.model.000081F.41_evm.model.000081F.42	NA	NA
evm.model.000216F.51	NA	NA
evm.model.000013F.125	NA	NA
evm.model.000145F.82	NA	NA
evm.model.000092F.63	NA	NA
evm.model.000237F.46	NA	NA
evm.model.000595F.4	NA	NA
evm.model.000199F.67.1	NA	NA
evm.model.000013F.288	NA	NA
evm.model.000333F.19	NA	NA
evm.model.000021F.88	NA	NA
evm.model.000016F.255	NA	NA
evm.model.000154F.95	NA	NA
evm.model.000277F.29	NA	NA
evm.model.000015F.62.1	NA	NA
evm.model.000255F.12	NA	NA
evm.model.000282F.27	NA	NA
evm.model.000042F.14	NA	NA
evm.model.000081F.52	NA	NA

evm.model.000040F.126	NA	NA
evm.model.000411F.8.1	NA	NA
evm.model.000016F.338	NA	NA
evm.model.000105F.10.1	NA	NA
evm.model.000035F.53	NA	NA
evm.model.000026F.186	NA	NA
evm.model.000030F.146	NA	NA
evm.model.000141F.63	NA	NA
evm.model.000033F.81	NA	NA
evm.model.000333F.14	NA	NA
evm.model.000173F.100	NA	NA
evm.model.000233F.35.1	NA	NA
evm.model.000083F.74	NA	NA
evm.model.000069F.65	NA	NA
evm.model.000106F.116	NA	NA
evm.model.000224F.74	NA	NA
evm.model.000010F.124	NA	NA

References

1. Vallerga MB, Mansilla SF, Federico MB, Bertolin AP, Gottifredi V. Rad51 recombinase prevents Mre11 nuclease-dependent degradation and excessive PrimPol-mediated elongation of nascent DNA after UV irradiation. *Proceedings of the National Academy of Sciences* **112**, e6624–e6633 (2015).
2. Maria SRS, Gangavarapu V, Johnson RE, Prakash L, Prakash S. Requirement of Nse1, a subunit of the Smc5-Smc6 complex, for Rad52-dependent postreplication repair of UV-Damaged DNA in *Saccharomyces cerevisiae*. *Molecular and Cellular Biology* **27**, 8409–8418 (2007).
3. Wang SJ, Zhuang KY, Zhang S, Yang MM, Kong FY, Meng QW. Overexpression of a tomato carotenoid ε-hydroxylase gene (*SlLUT1*) improved the drought tolerance of transgenic tobacco. *Journal of Plant Physiology* **222**, 103–112 (2018).
4. Saleem B*, et al.* Genome-wide and molecular characterization of the DNA *replication helicase 2 (DNA2)* gene family in rice under drought and salt stress. *Frontiers in Genetics* **13**, 1039548 (2022).
5. Abdallah NA, Elsharawy H, Abulela HA, Thilmony R, Abdelhadi AA, Elarabi NI. Multiplex CRISPR/Cas9-mediated genome editing to address drought tolerance in wheat. *GM Crops & Food*, 1–17 (2022).
6. Hirakawa T*, et al.* LSD1-LIKE1-mediated H3K4me2 demethylation is required for homologous recombination repair. *Plant Physiology* **181**, 499–509 (2019).
7. Lloyd JPB, Lang D, Zimmer AD, Causier B, Reski R, Davies B. The loss of SMG1 causes defects in quality control pathways in *Physcomitrella patens*. *Nucleic Acids Research* **46**, 5822–5836 (2018).
8. Wang C*, et al.* Comparative proteomic and phosphoproteomic analysis reveals differential heat response mechanism in two congeneric oyster species. *Ecotoxicology and Environmental Safety* **263**, 115197 (2023).
9. Daszkowska-Golec A, Karcz J, Plociniczak T, Sitko K, Szarejko I. Cuticular waxes—a shield of barley mutant in *CBP20* (*Cap-Binding Protein 20*) gene when struggling with drought stress. *Plant Science (Limerick)* **300**, 110593 (2020).
10. Guhr A, Horn MA, Weig AR. Vitamin B(2) (riboflavin) increases drought tolerance of *Agaricus bisporus*. *Mycologia* **109**, 860–873 (2017).
11. Venkatesh J, Park SW. Role of L-ascorbate in alleviating abiotic stresses in crop plants. *Botanical Studies* **55**, 38 (2014).
12. Soltabayeva A*, et al.* Ureides are accumulated similarly in response to UV-C irradiation and wounding in *Arabidopsis* leaves but are remobilized differently during recovery. *Journal of Experimental Botany* **73**, 1016–1032 (2022).
13. Xie YJ, Xu DK, Cui WT, Shen WB. Mutation of *Arabidopsis HY1* causes UV-C hypersensitivity by impairing carotenoid and flavonoid biosynthesis and the down-regulation of antioxidant defence. *Journal of Experimental Botany* **63**, 3869–3883 (2012).
14. Gou LM, Zhuo CL, Lu SY, Guo ZF. A Universal Stress Protein from *Medicago falcata (MfUSP1)* confers multiple stress tolerance by regulating antioxidant defense and proline accumulation. *Environmental and Experimental Botany* **178**, 104168 (2020).
15. Komatsu S*, et al.* Characterization of a novel flooding stress-responsive alcohol dehydrogenase

expressed in soybean roots. *Plant Molecular Biology* **77**, 309–322 (2011).

16. Bracher A, Verghese J. The nucleotide exchange factors of Hsp70 molecular chaperones. *Frontiers in Molecular Biosciences* **2**, 10 (2015).

17. Abdeen A, Schnell J, Miki B. Transcriptome analysis reveals absence of unintended effects in drought-tolerant transgenic plants overexpressing the transcription factor *ABF3*. *BMC Genomics* **11**, 69 (2010).

18. Su PH, Lin HY, Lai YH. Two Arabidopsis chloroplast GrpE homologues exhibit distinct biological activities and can form homo-and hetero-oligomers. *Frontiers in Plant Science* **10**, 1719 (2020).
