# Supplementary material for: Dipterocarpoidae genomics reveal their demography and adaptations to Asian rainforests
Source: Nat Commun. 2024 Feb 23;15:1683. doi: 10.1038/s41467-024-45836-5 (PMC10891123; doi:10.1038/s41467-024-45836-5)
Supplement: Supplementary file 6 — Reporting Summary [file 41467_2024_45836_MOESM6_ESM.pdf]

nature portfolio

Corresponding author(s): Rong Wang, Yuan-Ye Zhang, Xiao-Yong Chen

Last updated by author(s): Jan 9, 2024

Reporting Summary

Nature Portfolio wishes to improve the reproducibility of the work that we publish. This form provides structure for consistency and transparency in reporting. For further information on Nature Portfolio policies, see our Editorial Policies and the Editorial Policy Checklist.

Statistics

For all statistical analyses, confirm that the following items are present in the figure legend, table legend, main text, or Methods section.

n/a	Confirmed	
☐	☒	The exact sample size (*n*) for each experimental group/condition, given as a discrete number and unit of measurement
☐	☒	A statement on whether measurements were taken from distinct samples or whether the same sample was measured repeatedly
☐	☒	The statistical test(s) used AND whether they are one- or two-sided<br>*Only common tests should be described solely by name; describe more complex techniques in the Methods section.*
☐	☒	A description of all covariates tested
☐	☒	A description of any assumptions or corrections, such as tests of normality and adjustment for multiple comparisons
☐	☒	A full description of the statistical parameters including central tendency (e.g. means) or other basic estimates (e.g. regression coefficient) AND variation (e.g. standard deviation) or associated estimates of uncertainty (e.g. confidence intervals)
☐	☒	For null hypothesis testing, the test statistic (e.g. *F*, *t*, *r*) with confidence intervals, effect sizes, degrees of freedom and *P* value noted<br>*Give P values as exact values whenever suitable.*
☐	☒	For Bayesian analysis, information on the choice of priors and Markov chain Monte Carlo settings
☒	☐	For hierarchical and complex designs, identification of the appropriate level for tests and full reporting of outcomes
☒	☐	Estimates of effect sizes (e.g. Cohen's *d*, Pearson's *r*), indicating how they were calculated

Our web collection on statistics for biologists contains articles on many of the points above.

Software and code

Policy information about availability of computer code

Data collection	No software was used to collect data, and all data were obtained from our experiments and previous genomic studies (see our declaration of data availability for details).
Data analysis	All data analyses were conducted using programmes that have already been published, including Jellyfish version 2.1.3, Falcon and Falcon Unzip version 0.4.0, Smrtlink version 6.0.1, Pilon version 1.22, Hifiasm, Purge_haplogs version 1.1.0, BWA version 0.7.8, ALLHIC version 0.9.8, Lachesis version 201701, CEGMA version 2.5, BUSCO version 5.1.2, MCscanX, RepeatModeler version 1.0.5, LTR_FINDER version 1.0.7, RepeatScout version 1.0.5, RepeatMasker version 4.0.5, RepeatProteinMask version 4.0.5, TRF version 4.07b, tRNAscan-SE version 1.4, INFERNAL version 1.1.3, PASA version 2.3.3, Tophat version 2.0.13, Cufflinks version 2.1.1, Augustus version 3.0.2, Genscan version 1.0,.1 GlimmerHMM version 3.0.2, Geneid version 1.4, SNAP v11-29-2013, Solar version 0.9.6, GeneWise version 2.2.0, EvidenceModeler version 1.1.1, InterProScan version 4.7, HMMER version 3.1b2, InterPro version 29.0, OrthoMCL version 2.0.9, MUSCLE version 3.8.31, RAxML version 8.0.19, PAML version 4.9, KaKs_Calculator version 2, QUOTA-ALIGN, CAFE version 2.1, RGAugury, paircoil2, HMMsearch version 3.0, fastp version 0.19.7, SAMTOOLS version 1.16, GATK version 4.0.4.0, ANNOVAR, VCFtools version 0.1.14, SweeD version 3.2.1, Selscan version 1.2.0, InParanoid, SMC++ version 1.15.5, PSMC version 0.6.4-r49, PROVEAN version 1.1.5, SIFT4G version 2.0.0, PLINK version 1.07, ADMIXTURE version 1.23, TreeBeST version 1.9.2, Figtree version 1.4, and GCTA version 1.24.2. The in-house analysis scripts have been deposited in Github (https://github.com/Molecology /Dipterocarpoideae-genome).

For manuscripts utilizing custom algorithms or software that are central to the research but not yet described in published literature, software must be made available to editors and reviewers. We strongly encourage code deposition in a community repository (e.g. GitHub). See the Nature Portfolio guidelines for submitting code & software for further information.

Data

Policy information about availability of data

All manuscripts must include a data availability statement. This statement should provide the following information, where applicable:

- Accession codes, unique identifiers, or web links for publicly available datasets
- A description of any restrictions on data availability
- For clinical datasets or third party data, please ensure that the statement adheres to our policy

The whole-genome sequences (WGS) of our seven studied Dipterocarpoideae species and the 62 individuals used in the population genomic analysis have been deposited in the NCBI GenBank database, under the BioProject accession number PRJNA1056647 (https://www.ncbi.nlm.nih.gov/ bioproject/PRJNA1056647). The annotation files of the seven Dipterocarpoideae species have been deposited in the Figshare (https://figshare.com /articles/dataset/_/24936075). WGS of other species used in our analyses were downloaded from the sources as below: CNGBdb: CNP0002104 (13 Dipterocarpoideae species); NCBI: PRJNA788082 (Gossypium raimondii); DDBJ: PRJDB8161 and PRJDB8182 (Shorea leprosula); EMBL, GenBank, DDBJ: CACC01000001–CACC01025912 (Theobroma cacao); http://ftp. ensemblgenomes.org/pub/ plants/release-45/genbank/oryza_sativa (Oryza sativa); www.arabidopsis.org/cereon (Arabidopsis thaliana); iProX: PXD015597 (Aquilaria sinensis); NCBI: PRJNA212863 (Amborella trichopoda); ENA: PRJEB4958 (Fraxinus excelsior); ENA: PRJEB24056 (Fagus sylvatica); NCBI: PRJNA428013 (Ostrya rehderiana); ENA: PRJEB19898 (Quercus robur).

Research involving human participants, their data, or biological material

Policy information about studies with human participants or human data. See also policy information about sex, gender (identity/presentation), and sexual orientation and race, ethnicity and racism.

Reporting on sex and gender	N/A
Reporting on race, ethnicity, or other socially relevant groupings	N/A
Population characteristics	N/A
Recruitment	N/A
Ethics oversight	N/A

Note that full information on the approval of the study protocol must also be provided in the manuscript.

Field-specific reporting

Please select the one below that is the best fit for your research. If you are not sure, read the appropriate sections before making your selection.

☐ Life sciences ☐ Behavioural & social sciences ☒ Ecological, evolutionary & environmental sciences

For a reference copy of the document with all sections, see nature.com/documents/nr-reporting-summary-flat.pdf

Ecological, evolutionary & environmental sciences study design

All studies must disclose on these points even when the disclosure is negative.

Study description	Most Dipterocarpoideae species are endangered, leaving the question that how they established dominance but subsequently declined. In this study, we combined the de novo assemblies of seven Dipterocarpoideae species and the population genomic information from two seriously endangered Dipterocarpoideae species , to reveal the molecular footprints associated with their adaptation to tropical environments and the factors causing their endangerment. In the study, we first generated DNA sequencing data (Illumina and PacBio sequencing data, which were quantitative data) and assembled the genomes of the studied Dipterocarpoideae species (only one tree of each species was used in genome assembly). We then conducted phylogenomic analysis using our assembled genomes and 12 genomes of Dipterocarpoideae species from another study, and six genomes of species from other families were also used as the outgroup. The analysis testing genome duplication events was performed using our assembled genomes to detect the potential historical events relevant to the diversification and the autotetraploidization of two Hopea species. To reveal the positively selected genes that are likely to associated with adaptation of Dipterocarpoideae, we conducted comparative genomic analysis by setting the genomes of five temperate tree species as the control and our assembled genomes as the treatment. We then focused on population genomic analyses via sequencing the genomes of different individuals of the two endangered species (30 and 32 wild individuals were sampled for these two species, i.e., the nature number of units/replicates were 30 and 32 for all the following analyses), and the sequencing data were quantitative. After revealing patterns of genetic variation at the genome level by SNP (single nucleotide polymorphism loci) calling, the SNPs across the genome were obtained for each sampled individual. For both species, we performed population demographic analysis to uncover the population dynamics across different historical stages, these results can help infer historical events events likely contributing to the endangered status of these two species. Moreover, using SNP data, we also identified the derived deleterious mutations to assess the genetic load within each of the two species by comparing with the genome of a related species to detected the derived mutations. The genetic structure and coefficient of inbreeding at the genome level were analyzed using SNP data for evaluating the genetic consequences of recently occurred population decline.

Research sample	We selected seven species from five major genera of Dipterocarpoideae to typify this subfamily, of which the two species used in population genomic study are typical endangered species listed by IUCN. In our study, we collected fresh leaves from one morphologically well-identified tree for each selected species, to achieve high-quality de novo genome assembly. To perform evolutionary and comparative genomic analysis, we included many published plant genomes (see Supplementary Table 8 for details of species and the data source). As to the population genomic study, we focused on the remnant populations of Hopea hainanensis and Hopea reticulata on Hainan Island, China (the records of population locations are only available on Hainan Island, and thus the distribution of these two species is highly likely to be restricted on this island). Fresh leaves were sampled from 30 and 32 wild trees for either species to meet the requirement for sample size in each statistical analysis. These sampled trees were located in all areas where the two species were recorded (see Supplementary Fig. 12a and Supplementary Table 18 for details of sampling locations), and thus our samples can be the representative for the two species. Moreover, for the in vitro functional validation of positively selected genes, we carried out three replications to confirm the enzyme activity of each candidate gene.
Sampling strategy	Based on the morphological records and specimens in Xishuangbanna Tropical Botanical Garden and the Research Institute of Tropical Forestry (Chinese Academy of Forestry), we selected one morphologically well-identified tree for each selected species for genome assembly. Then, with the guidance of staff in local forestry stations, we conducted field surveys throughout the known distributional ranges of H. hainanensis and H. reticulata on Hainan Island, and located each observed wild tree of these two species. About 100 wild trees of H. hainanensis were found in four areas (sampling sites), and c. 200 wild trees of H. reticulata were found, but it is only distributed in one area (see Supplementary Table 18 for details). Given the small population size and limited distribution ranges of these two species, we considered that there was only one population in each species. To ensure the genetic independence between samples and that our sample size must meet the minimum sample size required by population genomic studies (which is generally thought as 30 sampled individuals/population), we decided to collect leaf samples from trees with a minimum interval of 100 m. Finally, our sample size reached to 30 and 32 for H. hainanensis and H. reticulata, respectively, which met the required standard for statistical analyses in population genomics.
Data collection	Sample collection for de novo genome assembly and population genomic study was conducted by Chao-Nan Liu, Xing-Hua Hu, Simon Segar, Shan Chen, Rong Wang, Yuan-Ye Zhang, Xiao-Yong Chen,Yuan-Yuan Ding, Yuan-Yuan Li, Gang Wang, Lu-Fan Chen, Stephen G Compton, Fang K Du, Run-Guo Zang, Dong-Hai Li, Ling Lu, Liang Tang, and Yang Yang. Experiments of the in vitro validation of enzyme activity was conducted by Kai Jiang, Jun-Yin Deng, Yu-Ting Jiang, Xin Tong and Rong Wang. Sequencing was carried out using Illumina and Pacbio platforms, and Kai-Jian Zhang, Chao-Nan Liu, Rong Wang, Xiao-Di Hu, Ling Kang, Wei-Wei Xu, and Zhuo-Xin Zu recorded the data.
Timing and spatial scale	Data collection in this study started from June 2018 and finished in May 2023. In June-December 2018, we selected the trees for de novo genome assembly, and conducted the field survey to determine the wild trees for population genomic study and collected leaves from these trees. From November 2018 to December 2020, genomes and transcriptomes of the seven Dipterocarpoideae species were sequenced and assembled. From June to July 2022, we performed in vitro validation of enzyme activity for the positively-selected genes associated with species' adaptation to tropical environments. To ensure the representativeness of our sampled trees, samples for the population genomic study covered the entire ranges of species distribution (c. 1000 km2 and 50 km2 for H. hainanensis and H. reticulata, respectively) reported by previous studies (the sampling locations are shown in Fig. 4d). From February to May 2023, we collected leaf tissues from the trees used for de novo genome assembly of our target species to conduct flow cytometry experiments to estimate their genome size and ploidy.
Data exclusions	No data were excluded.
Reproducibility	We sampled one individual tree from each species for de novo genome assembly and 30 and 32 wild trees of H. hainanensis and H. reticulata respectively for population genomic study. For de novo genome assembly, the generally accepted method is to only use the genomic DNA from one individual to ensure the quality of assembled genome. We obtained high depth of sequencing data for both Illumina and PacBio sequencing (see Supplementary Table 2) to assemble the high-quality reference genomes that can be reproduced by other independent studies if necessary. In addition to sufficient biological replicates (i.e., our sample size) for population genomic study, we sequenced the genome of each sample at a very high sequencing depth (> 40X; see Supplementary Table 18) to ensure that most regions of samples' genomes have been sequenced multiple times. Thus, incorrect genomic sequences were less likely to exist in our study, and the results of sequence mapping is expected to be reproducible. For the in vitro functional validation of positively selected genes, all the three replications of each candidate gene showed the presence of enzyme activity. Overall, all attempts to repeat experiments were successful.
Randomization	In this study, we chose the plants for population genomic study haphazardly.
Blinding	Different groups of researchers and postgraduates conducted the experiments, without being informed of the results obtained from other experiments in this study. Analyses were carried out using open source programmes, and therefore the algorithms were not specifically designed for our data.

Did the study involve field work? ☒ Yes ☐ No

Field work, collection and transport

Field conditions	The area for field work was mainly covered by tropical lowland rainforests and was dominated by monsoon climate with the average temperature of c. 24 degrees Celsius and the mean precipitation of 1600 mm. As the dominant tree species, the studied Dipterocarpoideae species mainly grows in the valleys and on the mountain sides covered by tropical forests. During the field work period, we collected fresh leaves from sampled trees for genome assembly.
Location	All field work was conducted in the reported distribution ranges of H. hainanensis and H. reticulata (E 108°15’-110°30’, N 18°36’-19° 46’, the locations of sampled trees are shown in Fig. 4d).

Access & import/export	The field works involved our study are permitted and assisted by local forestry stations.
Disturbance	No serious disturbance was caused in this study because we only sampled fresh leaves from trees, without destroying any of the sampled plants and their crops.

Reporting for specific materials, systems and methods

We require information from authors about some types of materials, experimental systems and methods used in many studies. Here, indicate whether each material, system or method listed is relevant to your study. If you are not sure if a list item applies to your research, read the appropriate section before selecting a response.

Materials & experimental systems

n/a	Involved in the study	
☒	☐	Antibodies
☒	☐	Eukaryotic cell lines
☒	☐	Palaeontology and archaeology
☒	☐	Animals and other organisms
☒	☐	Clinical data
☒	☐	Dual use research of concern
☐	☒	Plants

Methods

n/a	Involved in the study	
☒	☐	ChIP-seq
☐	☒	Flow cytometry
☒	☐	MRI-based neuroimaging

Dual use research of concern

Policy information about dual use research of concern

Hazards

Could the accidental, deliberate or reckless misuse of agents or technologies generated in the work, or the application of information presented in the manuscript, pose a threat to:

No	Yes	
☒	☐	Public health
☒	☐	National security
☒	☐	Crops and/or livestock
☒	☐	Ecosystems
☒	☐	Any other significant area

Experiments of concern

Does the work involve any of these experiments of concern:

No	Yes	
☒	☐	Demonstrate how to render a vaccine ineffective
☒	☐	Confer resistance to therapeutically useful antibiotics or antiviral agents
☒	☐	Enhance the virulence of a pathogen or render a nonpathogen virulent
☒	☐	Increase transmissibility of a pathogen
☒	☐	Alter the host range of a pathogen
☒	☐	Enable evasion of diagnostic/detection modalities
☒	☐	Enable the weaponization of a biological agent or toxin
☒	☐	Any other potentially harmful combination of experiments and agents

Flow Cytometry

Plots

Confirm that:

- [ ] The axis labels state the marker and fluorochrome used (e.g. CD4-FITC).
- [x] The axis scales are clearly visible. Include numbers along axes only for bottom left plot of group (a 'group' is an analysis of identical markers).
- [ ] All plots are contour plots with outliers or pseudocolor plots.
- [ ] A numerical value for number of cells or percentage (with statistics) is provided.

Methodology

Sample preparation	We declare that in this study we only used flow cytometry to estimate the genome size and ploidy of our target species, based on the approach proposed by Kron et al. (2012) and Ng et al. (2016) (see Methods). Therefore, these experiments are not involved in other aspects in cell biology, and plots, cell population abundance and gating strategy are not applicable for our experiments. When preparing samples, we selected 0.5 cm2 fresh young leaves from the sampled trees and finely chopped them into tissue debris.
Instrument	Sysmex CyFlow Cube6 (Sysmex-Partec, Germany)
Software	FCS Express 7plus (De Novo software, USA)
Cell population abundance	Not applicable
Gating strategy	Not applicable

- [ ] Tick this box to confirm that a figure exemplifying the gating strategy is provided in the Supplementary Information.
